# LncRNA MALAT1 mediates osteogenic differentiation in osteoporosis by regulating the miR-485-5p/WNT7B axis

**DOI:** 10.3389/fendo.2022.922560

**Published:** 2023-01-24

**Authors:** Yuan Zhou, Zhuo Xu, Yuanyi Wang, Qiang Song, Ruofeng Yin

**Affiliations:** ^1^ Department of Clinical Laboratory, the First Hospital of Jilin University, Changchun, Jilin, China; ^2^ Department of Rehabilitation, China-Japan Union Hospital of Jilin University, Changchun, Jilin, China; ^3^ Department of Spine Surgery, the First Hospital of Jilin University, Changchun, Jilin, China; ^4^ Department of Orthopedics, China-Japan Union Hospital of Jilin University, Changchun, Jilin, China

**Keywords:** osteoporosis, MALAT1, WNT7B, osteogenesis differentiation, MiR-485-5p

## Abstract

**Introduction:**

Accumulating evidence demonstrates that long non-coding RNAs (lncRNAs) are associated with the development of osteoporosis.

**Methods:**

This study aimed to investigate the effects of MALAT1 on osteogenic differentiation and cell apoptosis in osteoporosis. MALAT1 level, detected by RT-qPCR, was downregulated in hindlimb unloading (HU) mice and simulated microgravity (MG)-treated MC3T3-E1 cells. Moreover, osteogenic differentiation-related factor (Bmp4, Col1a1, and Spp1) levels were measured by RT-qPCR and Western blot. ALP activity was detected, and ALP staining was performed. Cell apoptosis was assessed by flow cytometry.

**Results:**

The results revealed that MALAT1 upregulated the expression of Bmp4, Col1a1, and Spp1, and enhanced ALP activity. Knockdown of MALAT1 suppressed their expression and ALP activity, suggesting that MALAT1 promoted osteogenic differentiation. Additionally, MALAT1 inhibited apoptosis, increased Bax and caspase-3 levels, and decreased Bcl-2 level. However, knockdown of MALAT1 had opposite results. In MG cells, MALAT1 facilitated osteogenic differentiation and suppressed apoptosis. Furthermore, miR-485-5p was identified as a target of MALAT1, and WNT7B was verified as a target of miR-485-5p. Overexpression of miR-485-5p rescued the promotion of osteogenic differentiation and the inhibition of apoptosis induced by MALAT1. Knockdown of WNT7B abolished the facilitation of osteogenic differentiation and the suppression of apoptosis induced by downregulation of miR-485-5p.

**Discussion:**

In conclusion, MALAT1 promoted osteogenic differentiation and inhibited cell apoptosis through the miR-485-5p/WNT7B axis, which suggested that MALAT1 is a potential target to alleviate osteoporosis.

## Introduction

Osteoporosis is a common systemic bone disease, which is characterized by bone loss and bone microstructure disorders (1, 2). The incidence of osteoporosis with aging reaches as high as 13.2%, which seriously affects the health and quality of life of the elderly (3). High glucose, anorexia nervosa, aging, and osteoblast dysfunction are the direct causes of osteoporosis (4). In normal bone tissue, osteoblasts and osteoclasts maintain a dynamic balance; however, this imbalance may lead to various bone diseases, including osteoporosis (5). Osteoblasts play a key role in bone formation or bone repair (6, 7). Therefore, promoting osteogenic differentiation of osteoblasts may help alleviate osteoporosis.

LncRNAs are a group of non-coding RNAs longer than 200 nt, which were considered as by-products produced by RNA polymerase II during transcription (8, 9). However, numerous studies have confirmed that lncRNAs exert multiple functions, such as direct or indirect transcriptional regulation, transcription factor isolation, and protein or RNA regulation (10). Metastasis-associated lung adenocarcinoma transcript 1 (MALAT1) located at human chromosome 11q13 and mouse chromosome 19q with a length of 8.5 kb (11) is a well-studied lncRNA in human diseases including osteoporosis (12). A previous report has revealed that MALAT1 enhances osteoblast activity to alleviate osteoporosis. Moreover, MALAT1 promotes osteoporosis progression through inhibiting osteogenic differentiation of bone marrow mesenchymal stem cells (13). However, the roles of MALAT1 in preosteoblast cells were still largely unknown.

WNT7B is a member of the WNT family, which is involved in various biological processes, such as proliferation, apoptosis, inflammatory response, and metabolism (14). WNT signaling is classified into canonical and noncanonical WNT pathways (15). The canonical WNT pathway is highly evolutionarily conserved and affects β-catenin. Previous studies evidence that WNT7B initiates β-catenin to regulate the occurrence and development of bone disorders (16, 17). For instance, Chen et al. (18) reveal that WNT7B rescues glucocorticoid-induced bone loss and suppresses secondary cause for osteoporosis. Moreover, WNT7B regulates glucose metabolism and promotes bone formation (19). Herein, hindlimb unloading (HU) and microgravity (MG) assays were performed to establish osteoporosis model *in vivo* and *in vitro*. We found that MALAT1 functioned as a ceRNA to upregulate the expression of WNT7B *via* sponging miR-485-5p. These findings extended our understanding of the function and mechanism of MALAT1 in osteoporosis development and may provide a new target for the treatment of osteoporosis.

## Materials and methods

### Establish osteoporosis mice model

Male C57BL/6J mice (6 months old) were provided by VitalRiver Laboratory Animal Co. Ltd. (Beijing, China). These animals were maintained under the following conditions: 21°C at a 12-h light/12-h dark cycle. According to a previous study (20), to establish the unloading model, the mice were suspended by the tail at an angle of approximately 30° with their forelimbs touching the floor, which allowed them to move and access food and water for free. The angle of suspension was adjusted to confirm that when the mice were fully stretching, their hindlimbs were unable to touch the ground. The tail suspension lasted for 3 weeks. Finally, these mice were sacrificed using anesthesia overdose (100 mg/kg pentobarbital). The bilateral femurs were dissected for micro-computed tomography (µCT) analysis. Animal studies were all approved by the Ethics Committee of the China–Japan Union Hospital of Jilin University and were performed according to the approved guidelines. Then, the bone tissues were collected and analyzed using a Micro-CT scanner (Scanco Medical AG, Switzerland).

Lentiviruses that overexpress MALAT1 (METTL14) and corresponding negative control (vector) were synthesized by Genepharma (Shanghai) and intramuscularly vertically injected into the lateral thigh muscle of the mice. Three consecutive injections were performed every 2 days. After injecting for 7 days, the unloading model was established.

### Simulated microgravity treatment

A 2D Rotating Wall Vessel Bioreactor (RWVB) clinostat was used to simulate microgravity. A total of 1 × 10^5^ MC3T3-E1 cells were seeded on cell climbing pieces, and 1×10^5^ MC3T3-E1 cells were seeded on coverslips. After culturing for 24 h, the climbing pieces were placed in a box 12.5 mm away from the rotational axis. After the air bubbles were removed, the chambers were fixed in the clinostat and rotated around a horizontal axis at 28 rpm for 15 min. The vertical rotation groups were used as controls. The rotation process was taken at 37°C under 5% CO_2_.

### Cell culture

The mouse pre-osteoblast cell line MC3T3-E1 was purchased from the American Type Culture Collection (ATCC, USA). Cells were cultured in α-MEM medium supplemented with 10% fetal bovine serum (FBS, Gibco), 100 U/ml penicillin, 100 μg/ml streptomycin, and 110 mg/ml sodium pyruvate under 5% CO_2_ at 37°C in a humidified atmosphere. For osteogenic differentiation, a specific culture was purchased from the Cell Bank of the Chinese Academy of Sciences and cultured in DMEM (Gibco, USA) supplemented with 10% fetal bovine serum (FBS, Gibco), 100 U/ml penicillin, 100 μg/ml streptomycin, and 110 mg/ml sodium pyruvate under 5% CO_2_ at 37°C in a humidified atmosphere. For osteogenic differentiation, a specific culture medium containing 100 nM dexamethasone, 50 µM ascorbic acid (Sigma), and 10 mM β-glycerophosphate was used.

### Cell transfection

Empty vector (pcDNA3.1), MALAT1 (pcDNA3.1-MALAT1), siRNA (si)-nc, si-MALAT1, mimic negative control (nc), miR-485-5p mimic, inhibitor nc, miR-485-5p inhibitor, and si-WNT7B were synthesized by Hanbio (Shanghai, China). MC3T3-E1 cells were seeded in six-well plates and transiently transfected using Lipofectamine™ 2000 (Invitrogen, Carlsbad, CA, USA) according to the instructions. After 6 h of incubation, the medium was replaced by a complete medium.

### RT-qPCR

Total RNA was isolated *via* TRIzol reagent (Invitrogen, Carlsbad, CA, USA). After the concentration determined the absorbance (A)260/A280 ratio, RNA was reverse transcribed to cDNA using SuperScript reverse transcriptases (for mRNA; Thermo Fisher Scientific, Waltham, MA, USA) and the One-Step PrimeScript miRNA cDNA Synthesis Kit (for miRNA; Takara, Tokyo, Japan). qPCR for mRNA was conducted by SYBR PCR Master Mix (GenePharma, Shanghai, China) under the following conditions: 95°C for 3 min, 40 cycles of 95°C for 12 s and 62°C for 40 s. For miRNA, qPCR was performed using SYBR Premix Ex Taq II (Perfect Real Time) (Takara, Tokyo, Japan) under the following conditions: 95°C for 10 s, 40 cycles of 95°C for 5 s and 60°C for 20 s. All samples were repeated three times. The expression level of genes was calculated using the 2^−ΔΔCT^ method. GAPDH and U6 were used as the internal control for mRNA and miRNA, respectively.

### Luciferase reporter assay

The luciferase reporter vector pGL3.1 carrying wild-type binding site (MALAT1-wt or WNT7B-wt) or mutant-type binding site (MALAT1-mut or WNT7B-mut) was constructed by RiboBio (Guangzhou, China). 293T cells were transfected with the MALAT1-wt or MALAT1-mut along with miR-485-5p mimic or mimic control. Renilla luciferase plasmid was transfected as an internal control. After 48 h, according to the instructions of the dual luciferase detection kit, a microplate reader was used to assess the firefly and Renilla luciferase activity in each group. The ratio of firefly fluorescence intensity to Renilla fluorescence intensity reflects the relative fluorescence intensity of each group.

### RNA pull-down

The biotinylated probe of miR-485-5p and the control probe were synthesized by Shenggong Biotech (Shanghai, China). Then, the probe was incubated with streptavidin-coated beads (Invitrogen, Carlsbad, CA, USA) at 25°C for 2 h to generate the probe-coated beads. The streptomycin beads were capable of binding to biotin. Cells were lysed to extract total RNA. After pretreatment of magnetic beads, RNA and beads were mixed. After separation, qPCR was used to quantify the relative expression of MALAT1 or WNT7B.

### Western blot

The total proteins were extracted with lysis buffer containing protease inhibitor and phosphatase inhibitor cocktail (Sigma, CA, USA). Proteins (40 μg) were separated in 10% SDS-PAGE gel and then transferred onto polyvinylidene fluoride (PVDF) membranes (Millipore, USA). Subsequently, the membranes were blocked in 5% non-fat dry milk in TBST buffer for 1 h at room temperature. Thereafter, the blots were incubated with the primary antibodies 4°C overnight followed by incubating with the horseradish peroxidase-conjugated secondary antibody for another 2 h at room temperature. Finally, the blots were visualized using an enhanced chemiluminescence kit (Beyotime, Shanghai, China). The bands were quantified using ImageJ software.

### Alkaline phosphatase activity

The alkaline phosphatase (ALP) activity of cells was evaluated using an Alkaline Phosphatase Assay Kit (Beyotime, Shanghai, China) according to the manufacturer’s instructions. Briefly, lysed cells were incubated with test buffer at 37°C for 10 min. A reaction stopping solution (100 μl) was used to terminate the reaction. A microreader (Bio-Rad, USA) was used to assess the OD value at 490 nm.

### ALP staining

The ALP staining was conducted in the dark and assayed with the BCIP/NBT Alkaline Phosphatase Color Development according to the manufacturer’s instructions (Beyotime, Shanghai, China). The images were taken under a light microscope.

### Alizarin red staining

After transfection, cells were collected and fixed with 4% paraformaldehyde for 15 min. After washing thrice with PBS, cells were cultured with osteogenic induction medium for 3 weeks and then stained with alizarin red reagents for 30 min. Calcified nodules were captured using a microscope and calculated using a spectrophotometric wavelength of 570 nm.

### Flow cytometry

After transfection and treatment, cells were washed thrice with PBS. Then, the cells were stained with 2.5 μg/ml of Annexin V-FITC and propidium iodide and incubated at room temperature in the dark for 15 min. Finally, the cells were captured with a fluorescent microscope and evaluated by a FACSVerse flow cytometer system (BD, USA) with FlowJo software.

### Statistical analysis

All the data are presented as means ± standard deviation (SD). The statistical analyses were performed using SPSS version 17. Data were analyzed with one-way ANOVA and Student’s *t*-test. *p* < 0.05 was considered statistically significant. Pearson analysis was employed to evaluate the correlations between MALAT1 and miR-485-5p as well as between miR-485-5p and WNT7B.

## Results

### The expression of MALAT1 was decreased in MG-treated cells

**Figure 1:**
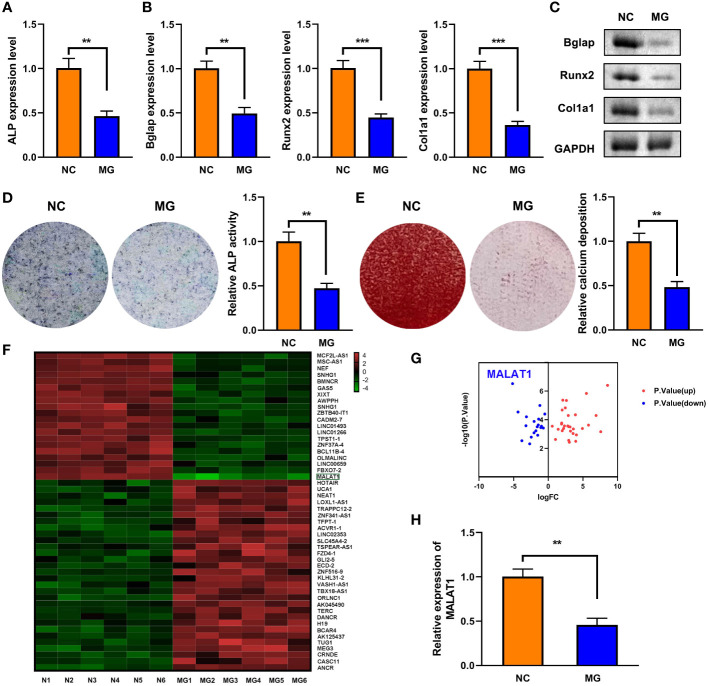
The expression of MALAT1 was downregulated MG-treated cells. **(A)** The expression of ALP in the MG-treated MC3T3-E1 cells was determined using RT-qPCR. **(B)** The mRNA expressions of Bglap, Runx2, and Col1a1 in the MG-treated MC3T3-E1 cells were measured by RT-qPCR. **(C)** The protein expressions of Bglap, Runx2, and Col1a1 in the MG-treated MC3T3-E1 cells were detected using Western blot. **(D)** ALP activity in the MG-treated MC3T3-E1 cells was detected using ALP staining. **(E)** Alizarin red staining was used to measure calcium deposit in the MG-treated MC3T3-E1 cells. **(F, G)** The differentially expressed lncRNAs in MG-treated MC3T3-E1 cells was expressed as heat maps and volcano maps. **(H)** The expression of MALAT1 in MG-treated MC3T3-E1 cells was detected using RT-qPCR. **p < 0.01, ***p < 0.001.

As shown in **Figure 1A**, MG exposure significantly deceased ALP expression. Moreover, MG treatment suppressed the mRNA and protein expression of Bglap, Runx2, and Col1a1 (**Figures 1B, C**). Additionally, MG significantly decreased ALP activity and calcium deposit in MC3T3-E1 cells (**Figures 1D, E**). LncRNAs play a vital role in the initiation and development of osteoporosis. Therefore, we determined the potentials of lncRNAs in MG-treated MC3T3-E1 cells. The results from cell sequencing showed that the expression of lncRNA MALAT1 was mostly downregulated (**Figures 1F**, **G**). To further verify the roles of MALAT1 in osteoporosis, we determined its expression in MG-treated MC3T3-E1 cells. As shown in **Figure 1H**, the expression of MALAT1 was significantly decreased in the MG group. Additionally, as shown in **Supplementary Figure 1**, the expression of MALAT1 was significantly increased in the MC3T3-E1 cells during osteogenic differentiation.

### MALAT1 promoted osteogenic differentiation and inhibited cell apoptosis in MC3T3-E1 cells

**Figure 2:**
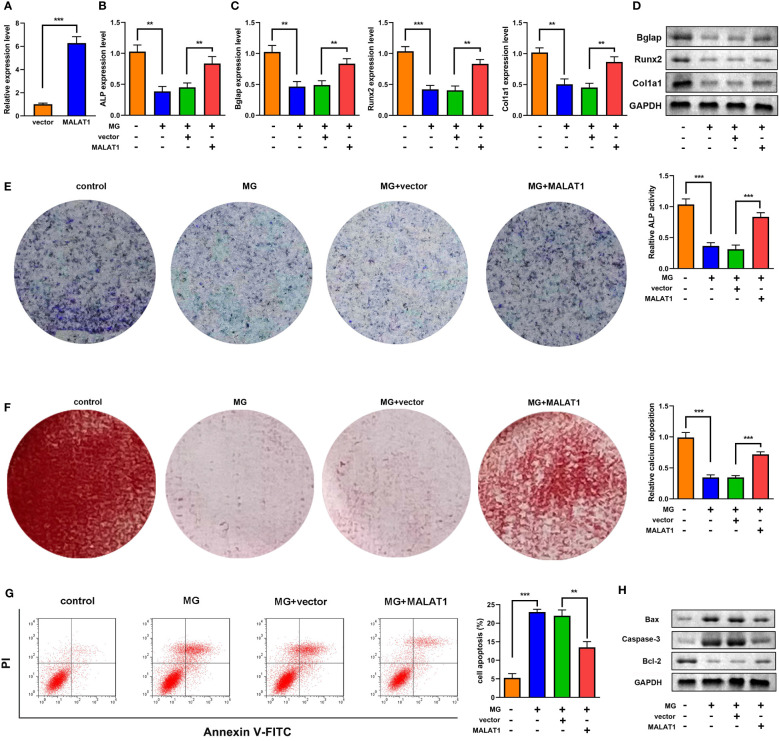
MALAT1 facilitated MC3T3-E1 cell osteogenic differentiation and suppressed apoptosis. **(A)** The transfection efficiency of MALAT1 was determined using RT-qPCR. Then, the MC3T3-E1 cells were treated with MG and transfected with MALAT1. **(B)** The expression of ALP was determined using RT-qPCR. **(C)** The mRNA expression of Bglap, Runx2, and Col1a1 was determined using RT-qPCR. **(D)** The protein expression of Bglap, Runx2, and Col1a1 was detected using Western blot. **(E)** The ALP activity of MC3T3-E1 cells. **(F)** Alizarin red staining was used to measure calcium deposit. **(G)** Cell apoptosis was analyzed by flow cytometry, and cell apoptosis rate was quantified. **(H)** The protein expression of Bax, caspase-3, and Bcl-2 was detected using Western blot. **P<0.01, ***P<0.001.

To explore the effects of MALAT1 on osteogenic differentiation, cells were transfected with MALAT1 overexpression plasmids. As shown in **Figure 2A**, the expression of MALAT1 was significantly increased in the MALAT1 group, suggesting that cells were successfully transfected. Overexpression of MALAT1 significantly increased the expression of ALP, Bglap, Runx2, and Col1a1 (**Figures 2B–D**). Quantification of protein bands is shown in **Supplementary Figure 4**. Moreover, overexpressed MALAT1 significantly increased ALP activity and calcified nodules (**Figures 2E, F**). To further verify the potential roles of MALAT1 in osteoporosis, we further determined the effects of MALAT1 on the cellular function of MC3T3-E1 cells. As shown in **Figure 2G**, the increase in the apoptosis of MC3T3-E1 cells induced by MG was alleviated by overexpressed MALAT1. Moreover, upregulation of MALAT1 increased the protein expression of the pro-proliferation gene Bcl-2 and decreased apoptosis-related genes such as Bax and Caspase-3 (**Figure 2H**).

### Overexpression of MALAT1 alleviated syndromes of osteoporosis

**Figure 3:**
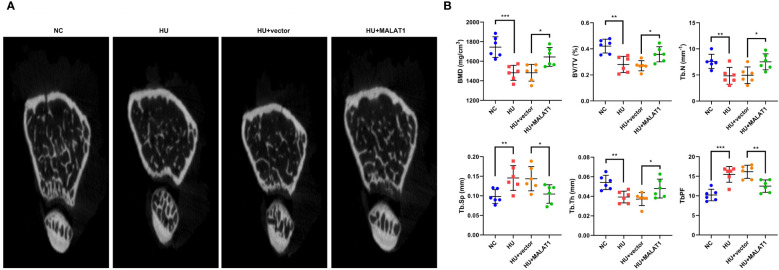
MALAT1 improved bone phenotype in HU mice. **(A)** Micro-CT images of distal femurs of mice. The **(B)** BMD, BV/TV, Tb.N, Tb.Sp, Tb.Th, and TbPF were analyzed by Micro-CT. *p < 0.05, **p < 0.01, ***p < 0.001.

To further clarify the roles of MALAT1 in osteoporosis, we detected the effects of MALAT1 overexpression on osteoporosis *in vivo*. As shown in **Figure 3A**, the results from micro-CT assays showed that the bone mineral density was decreased in the HU group; however, overexpressed MALAT1 increased the bone mineral density compared with the HU + vector group. Moreover, the decrease of the ratio of bone volume to total volume (BV/TV), trabecular bone number (Tb.N), and trabecular thickness (Tb.Th) and the increase in the values of the bone trabecula separation (Tb. Sp) and trabecular bone pattern factor (Tb.PF) induced by HU were alleviated by overexpressed MALAT1 (**Figure 3B**).

### MALAT1 served as a sponge to miR-485-5p

**Figure 4:**
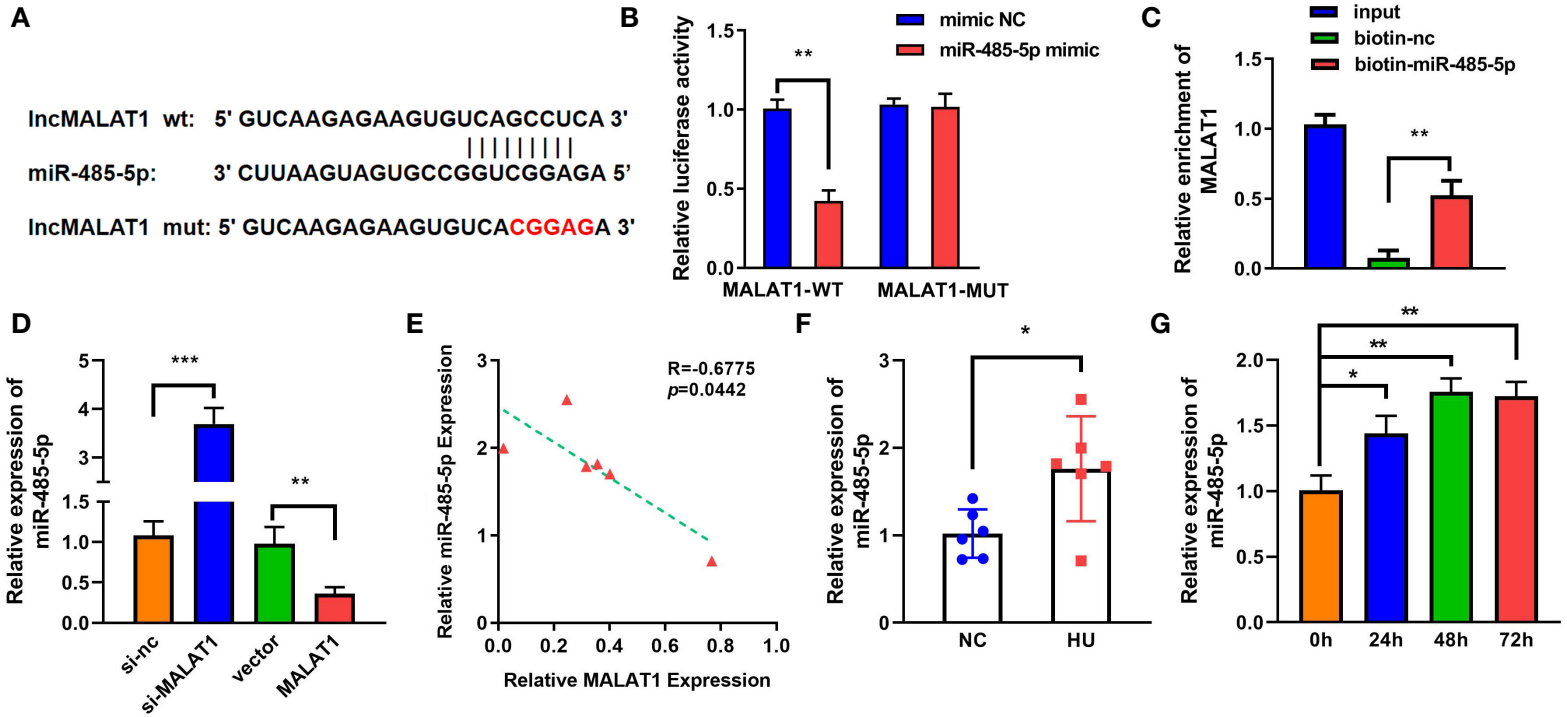
MALAT1 acted as a miRNA sponge of miR-485-5p. **(A)** The binding sites of wild-type (wt) MALAT1 and miR-485-5p were shown. Meanwhile, mutant (mut) MALAT1 sequences were also shown. **(B)** The targeting relationship between MALAT1 and miR-485-5p was confirmed by luciferase reporter assay. **(C)** The targeted relationship between MALAT1 and miR-485-5p was verified using RNA pull-down. **(D)** The expression of miR-485-5p was measured by RT-qPCR after overexpression or MALAT1 knockdown. **(E)** The Pearson method was applied for correlation analysis. **(F, G)** The expression of miR-485-5p in the HU mice and MG-treated MC3T3-E1 cell was detected using RT-qPCR. *p < 0.05, **p < 0.01, ***p < 0.001.

LncRNAs functions as a ceRNA *via* sponging miRNAs. **Figure 4A** shows the binding sites between MALAT1 and miR-485-5p. Moreover, miR-485-5p mimic significantly decreased the luciferase activity in the WT-MALAT1 group compared with mimic nc, but there was no significant difference in the MUT-MALAT1 group (**Figure 4B**). RNA pull-down further confirmed the interaction between MALAT1 and miR-485-5p (**Figure 4C**). The expression of miR-485-5p was significantly upregulated by silenced MALAT1 and downregulated by overexpressed MALAT1 (**Figure 4D**). Moreover, miR-485-5p expression was elevated and was negatively correlated with MALAT1 in HU mice (**Figures 4E, F**). Additionally, the expression of miR-485-5p was increased by MG in a time-dependent manner (**Figure 4G**). As shown in **Supplementary Figure 3**, the expression of miR-485-5p was significantly decreased in the MC3T3-E1 cells during osteogenic differentiation.

### MALAT1 promoted the osteogenic differentiation *via* sponging miR-485-5p

**Figure 5:**
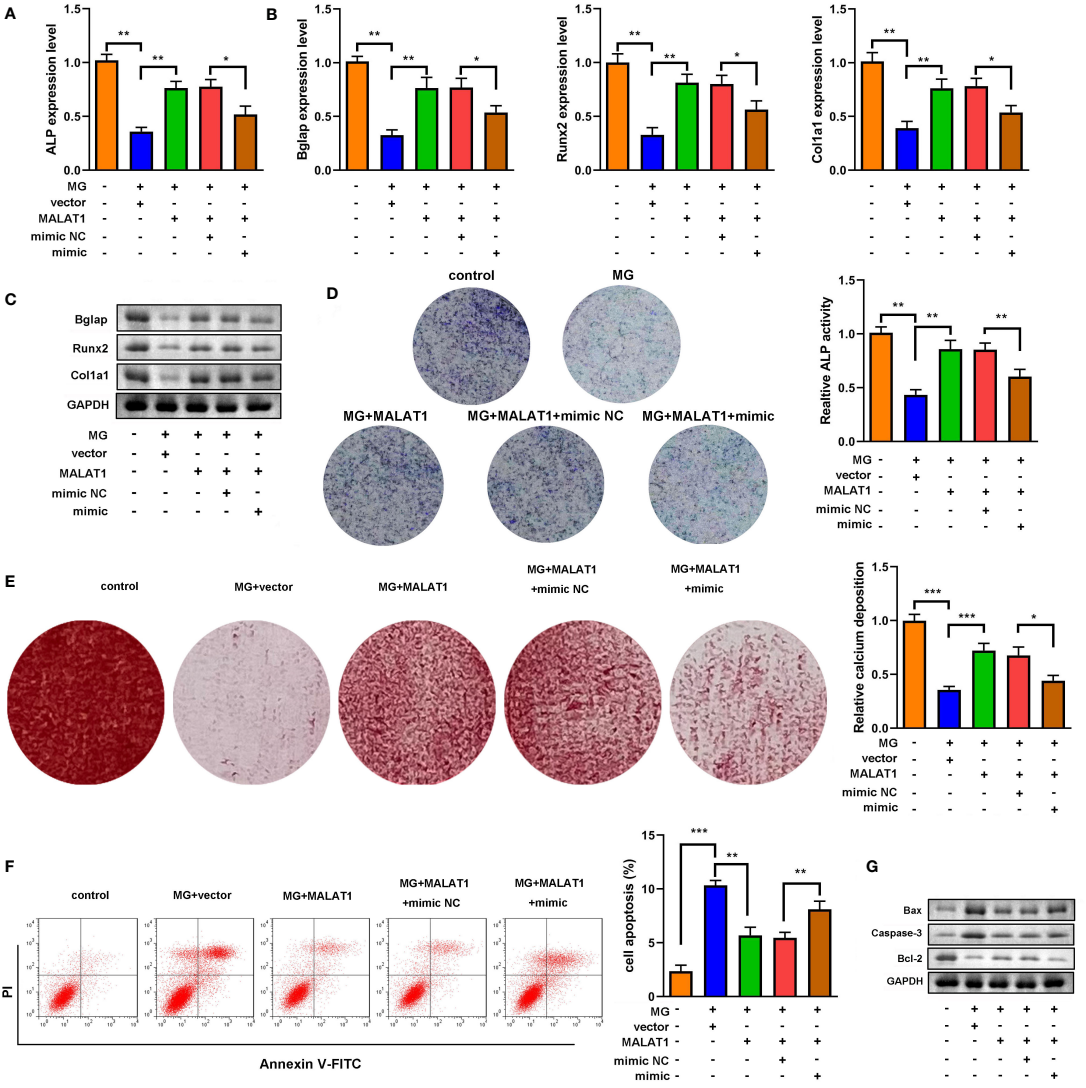
miR-485-5p attenuated osteogenic differentiation induced by MALAT1. **(A)** The MG-treated MC3T3-E1 cell was transfected with MALAT1 and miR-485-5p mimic. **(A)** The expression of ALP was detected by RT-qPCR. **(B)** The expression of Bglap, Runx2, and Col1a1 was detected by RT-qPCR. **(C)** The protein expression of Bglap, Runx2, and Col1a1 was detected by Western blot. **(D)** The ALP activity of MC3T3-E1 cells. **(E)** The calcium deposit detected using Alizarin red staining. **(F)** The apoptosis of MC3T3-E1 cells detected using flow cytometry. **(G)** The protein expression of Bax, Caspase-3, and Bcl-2 detected using Western blot. *p < 0.05, **p < 0.01, ***p < 0.001.

As shown in **Figures 5A–C**, overexpression of miR-485-5p decreased the expression of ALP, Bglap, Runx2, and Col1a1. As illustrated in **Figures 5B–E**, the expression of Bmp4, Col1a1, and Spp1 at both mRNA and protein levels was elevated by MALAT1, which were rescued by miR-485-5p. Moreover, the increase in ALP activity induced by MALAT1 was abolished by miR-485-5p mimic (**Figure 5D**). This was inconsistent with the results from Alizarin red staining. As shown in **Figure 5E**, overexpressed miR-485-5p significantly decreased the calcium deposit of MC3T3-E1 cells (**Figure 5E**). Moreover, miR-485-5p significantly increased the apoptosis rates of MC3T3-E1 cells as well as increased the expression of apoptosis-related genes, such as Bax and Caspase-3 and decreased Bcl-2 (**Figures 5F, G**). Additionally, we further explore the effects of miR-485-5p mimic alone on the MG-treated MC3T3-E1 cells. As shown in **Supplementary Figure 5**, we found that miR-485-5p overexpression aggravated the inhibitory effect of MG on osteoblastic differentiation and the promoting effect of MG on apoptosis of MC3T3-E1 cells.

### WNT7B was a target of miR-485-5p

**Figure 6:**
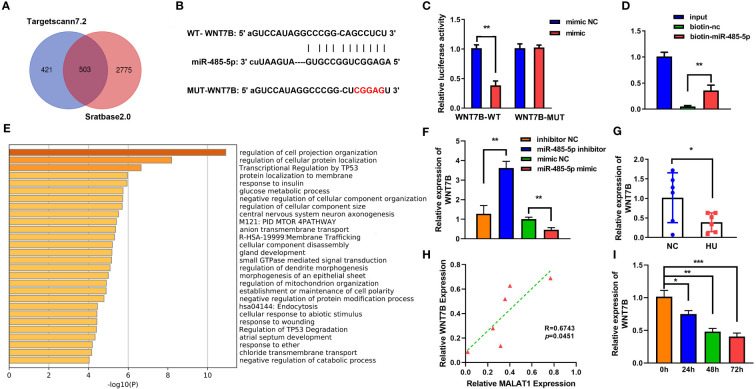
WNT7B is a direct target of miR-485-5p. **(A)** The potential target of miR-485-5p was predicted by Starbase2.0 and TargetScan7.2. **(B)** Potential binding sites for miR-485-5p to WNT7B were predicted by TargetScan. **(C)** The targeted relationship between miR-485-5p and WNT7B was confirmed by dual-luciferase reporter assay. **(D)** The targeted relationship between miR-485-5p and WNT7B was confirmed by RNA pull-down. **(E)** GO analysis of biological processes. **(F)** The expression of WNT7B was assessed by RT-qPCR after miR-485-5p overexpression and knockdown. **(G)** The expression of WNT7B *in vivo*. **(H)** The correlation between the expression of WNT7B and MALAT1 analyzed using Pearson analysis. **(I)** The expression of WNT7B detected using RT-qPCR. *p < 0.05. **p < 0.01. ***P<0.001.

To further explore the mechanism of miR-485-5p, Starbase (http://starbase.sysu.edu.cn/index.php) and TargetScan were applied to predict the target of miR-485-5p (**Figure 6A**). **Figure 6B** shows the binding sites between miR-485-5p and WNT7B. The interaction between miR-485-5p and WNT7B was further verified by luciferase and RNA pull-down assay (**Figures 6C, D**). GO analysis showed that WNT7B was a regulator of cellular signal transmission, cell growth, and metabolism (**Figure 6E**). Inhibition of miR-485-5p significantly upregulated the expression of WNT7B, while overexpression of miR-485-5p downregulated WNT7B level (**Figure 6F**). The expression of WNT7B was lower in HU mice than in the NC group (**Figure 6G**). Moreover, WNT7B expression was positively correlated with MALAT1 (**Figure 6H**). WNT7B expression was downregulated by MG in a time-dependent manner (**Figure 6I**). Additionally, as shown in **Supplementary Figure 3**, the expression of WNT7B was significantly increased in the MC3T3-E1 cells during osteogenic differentiation.

### Inhibition of miR-485-5p promoted osteogenic differentiation by targeting WNT7B

**Figure 7:**
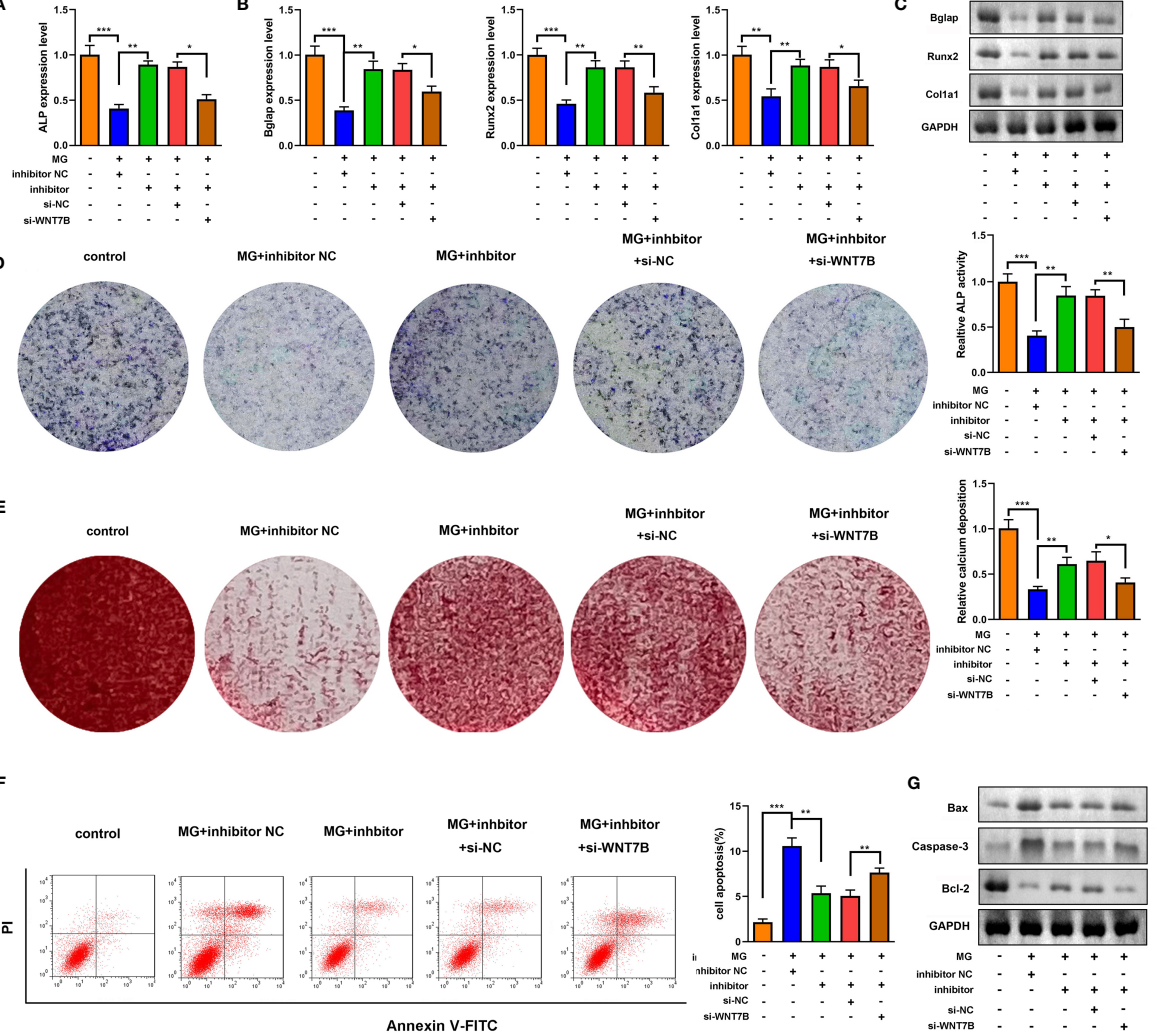
WNT7B was a negative regulator of miR-485-5p on the osteogenic differentiation. The MG-treated MC3T3-E1 cell was transfected with miR-485-5p inhibitor and si-WNT7B. **(A)** The expression of ALP was detected by RT-qPCR. **(B)** The expression of Bglap, Runx2, and Col1a1 was detected by RT-qPCR. **(C)** The protein expression of Bglap, Runx2, and Col1a1 was detected by Western blot. **(D)** The ALP activity of MC3T3-E1 cells. **(E)** The calcium deposit detected using Alizarin red staining. **(F)** The apoptosis of MC3T3-E1 cells detected using flow cytometry. **(G)** The protein expression of Bax, Caspase-3, and Bcl-2 detected using Western blot. *p < 0.05, **p < 0.01, ***p < 0.001.

Downregulated WNT7B alleviated the effects of miR-485-5p inhibitor on the expression of ALP, Bglap, Runx2, and Col1a1 (**Figures 7A–C**). Moreover, ALP staining and Alizarin red staining showed that WNT7B knockdown significantly suppressed ALP activity and calcium deposit of MC3T3-E1 cells (**Figures 7D, E**). Additionally, WNT7B knockdown suppressed the apoptosis of MC3T3-E1 cells as well as alleviated the effects on the protein expression of Bcl-2, Bax, and Caspase-3 (**Figures 7F, G**). Furthermore, we further explore the effects of si-WNT7B alone on the MG-treated MC3T3-E1 cells and the effect of si-wnt7b + miR-485-5p inhibitor on MC3T3-E1 cells. As shown in **Supplementary Figure 6**, we found that WNT7B knockdown aggravated the inhibitory effect of MG on osteoblastic differentiation and the promoting effect of MG on apoptosis of MC3T3-E1 cells. As shown in **Supplementary Figure 7**, we found that miR-485-5p knockdown promoted the osteogenic differentiation of MC3T3-E1 cells and showed no effects on the apoptosis rate of MC3T3-E1 cells, while WNT7B knockdown reversed the effects of miR-485-5p inhibitor on the osteogenic differentiation of MC3T3-E1 cells and enhanced the apoptosis rate of the miR-485-5p inhibitor-treated MC3T3-E1 cells.

## Discussion

These findings verified the protective roles of MALAT1 in osteoporosis. MALAT1 was downregulated in osteoporosis models *in vivo* and *in vitro*. Its downregulation was associated with the accumulation of ROS and mitochondrial damage, which suppressed the osteogenesis and promoted the apoptosis of MC3T3-E1 cells. This further exacerbated the development of osteoporosis, which was characterized by the decrease of MC3T3-E1 cells. However, overexpression of MALAT1 suppressed the release of ROS and promoted osteogenesis. This may be attributed to its ceRNA properties. MALAT1 sponged miR-485-5p to activate WNT7B. These results further identified that MALAT1 can be a therapeutic target for osteoporosis.

The bone-protective roles of MALAT1 seem surprising. Previous studies report that MALAT1 functions as an oncogene in orthopedic malignancies and exerts pro-inflammatory properties in bone disorders, including osteoarthritis, osteolysis and ossification (12; Gao et al., 2021; 21–23). However, emerging evidence reveals that MALAT1 protects against osteonecrosis of the femoral head and osteoporosis. This may be due to its positive roles in osteogenesis. For instance, mesenchymal stem cell-derived MALAT1 promotes human osteogenic differentiation and suppresses the development of osteoporosis. MALAT1 promotes osteoblast differentiation of adipose-derived mesenchymal stem cells, which further induces bone tissue repair and regeneration. The present study further verified the beneficial roles in treating osteoporosis. MALAT1 was downregulated in osteoporosis, and its downregulation was associated with the degradation of MC3T3-E1 cells. However, upregulated MALAT1 increased the expression of osteogenesis-related genes, such as ALP, RUNX2, and Col1a1, increased ALP activity and calcium deposition, and suppressed the apoptosis of MC3T3-E1 cells. These results suggested that MALAT1 exerted its protective roles in osteoporosis *via* promoting osteogenic differentiation of MC3T3-E1 cells. Stimulation of osteoblast and osteogenesis is pronounced in bone anabolic action (24), which plays a key role in developing bones. Osteogenesis imperfecta is deeply associated with the primary osteoporosis (25). Thus, osteogenesis may be the Achilles’ heel of osteoporosis.

LncRNAs function as ceRNA to regulate gene expression and biological processes *via* sponging microRNAs. Dysregulated lncRNAs modulate osteogenic differentiation, proliferation, apoptosis, adipogenesis, bone formation, and necrosis. In this study, dysregulated MALAT1 contributed to the degradation of MC3T3-E1 cells and the development of osteoporosis *via* sponging miR-485-5p. miR-485-5p was upregulated in osteoporosis patients (26). However, its downregulation promoted the osteogenic differentiation of human bone marrow-derived mesenchymal stem cells (27). Therefore, miR-485-5p exacerbated the development of osteoporosis. In this study, overexpression of miR-485-5p suppressed not only osteogenic differentiation but also the apoptosis of MC3T3-E1 cells. These results suggested that MALAT1 alleviated osteoporosis by sponging miR-485-5p.

WNT7B, a member of the WNT family, facilitates bone formation. Chen et al. (18) reveal that WNT7B promotes glucose consumption and osteoblast differentiation. WNT7B induces the mineralization of the subsequent bone callus and trabecular and endosteal bone formation. Moreover, WNT7B promotes self-renewal and osteogenic differentiation of bone marrow mesenchymal stem cells (28). These results dictate that WNT7B may play a beneficial role in osteoporosis *via* regulating metabolism processes. This bone anabolic function may be a promising therapy for osteoporosis. In this study, MALAT1 sponged miR-485-5p to activate WNT7B, which was downregulated in osteoporosis model *in vivo* and *in vitro*. WNT7B knockdown contributed to the increase of oxidative stress and suppressed osteogenic differentiation of bone marrow mesenchymal stem cells. These results further verified that WNT7B protected against osteoporosis, which is consistent with previous studies. WNT7B modulates glucose metabolism *via* upregulating Glut1 (19). Yu et al. (28) demonstrate that WNT7B exerts its bone anabolic function *via* activating Sox11. Therefore, we further investigated the underlying mechanisms. Further studies are needed to unveil the underlying molecular mechanisms.

In conclusion, the low expression of MALAT1 was closely associated with the development of osteoporosis. Overexpression of MALAT1 promoted osteogenic differentiation of bone marrow mesenchymal stem cells and suppressed oxidative stress *via* activating the miR-485-5p/WNT7B axis. This may be a promising therapy for osteoporosis.

## Data availability statement

The original contributions presented in the study are included in the article/**Supplementary Material**. Further inquiries can be directed to the corresponding author.

## Ethics statement

The animal study was reviewed and approved by the Ethics Committee of China-Japan Union Hospital of Jilin University. Written informed consent was obtained from the owners for the participation of their animals in this study.

## Author contributions

ZX and YW conceived the study; QS conducted the experiments; RY analyzed the data; YZ wrote the manuscript; all the authors read and approved the final version of the manuscript.

## Supplementary material

Supplementary Figure 1 MALAT1 was upregulated during osteogenic differentiation. **P<0.01.

Supplementary Figure 2 miR-485-5p was downregulated during osteogenic differentiation. **P<0.01.

Supplementary Figure 3 WNT7B was upregulated during osteogenic differentiation. **P<0.01.

Supplementary Figure 4 Quantification of the blots in . *P<0.05, **P<0.01.

Supplementary Figure 5 miR-485-5p aggravated inhibitory effects of MG on osteogenic differentiation. The MG treated MC3T3-E1 cell was transfected with miR-485-5p mimic. **(A)** The expression of ALP was detected by RT-qPCR. **(B)** The expression of Bglap, Runx2, and Col1a1 was detected by RT-qPCR. **(C)** The protein expression of Bglap, Runx2, and Col1a1 was detected by western blot. **(D)** The ALP activity of MC3T3-E1 cells. **(E)** The calcium deposit detected using Alizarin red staining. **(F)** The apoptosis of MC3T3-E1 cells detected using flow cytometry. **(G)** The protein expression of Bax, Caspase-3, and Bcl-2 detected using western blot. *P<0.05, **P<0.01.

Supplementary Figure 6 WNT7B knockdown aggravated inhibitory effects of MG on osteogenic differentiation. The MG treated MC3T3-E1 cell was transfected with si-WNT7B. **(A)** The expression of ALP was detected by RT-qPCR. **(B)** The expression of Bglap, Runx2, and Col1a1 was detected by RT-qPCR. **(C)** The protein expression of Bglap, Runx2, and Col1a1 was detected by western blot. **(D)** The ALP activity of MC3T3-E1 cells. **(E)** The calcium deposit detected using Alizarin red staining. **(F)** The apoptosis of MC3T3-E1 cells detected using flow cytometry. **(G)** The protein expression of Bax, Caspase-3, and Bcl-2 detected using western blot. *P<0.05, **P<0.01.

Supplementary Figure 7 WNT7B knockdown reversed the promoting effects of miR-485-5p inhibitor on osteogenic differentiation in MC3T3-E1 cells. The MC3T3-E1 cell was transfected with si-WNT7B and miR-485-5p inhibitor. **(A)** The expression of ALP was detected by RT-qPCR. **(B)** The expression of Bglap, Runx2, and Col1a1 was detected by RT-qPCR. **(C)** The protein expression of Bglap, Runx2, and Col1a1 was detected by western blot. **(D)** The ALP activity of MC3T3-E1 cells. **(E)** The calcium deposit detected using Alizarin red staining. **(F)** The apoptosis of MC3T3-E1 cells detected using flow cytometry. **(G)** The protein expression of Bax, Caspase-3, and Bcl-2 detected using western blot. *P<0.05, **P<0.01.
