# Supplementary figures and images for: LncRNA MALAT1 mediates osteogenic differentiation in osteoporosis by regulating the miR-485-5p/WNT7B axis
Source: Front Endocrinol (Lausanne). 2023 Jan 24;13:922560. doi: 10.3389/fendo.2022.922560 (PMC9904362; doi:10.3389/fendo.2022.922560)

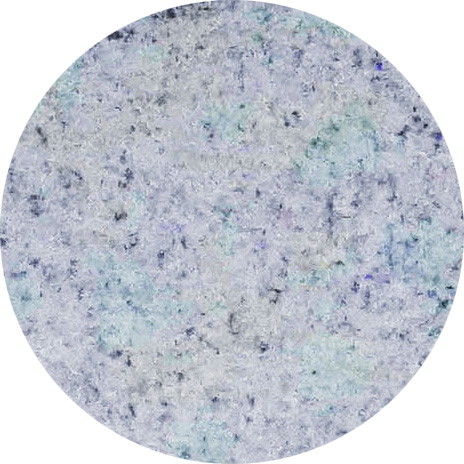

Supplement: Supplementary Figure 1 — MALAT1 was upregulated during osteogenic differentiation. **P<0.01. [file DataSheet_1.zip › MALAT1/ALP/Fig.1/MG.jpg]

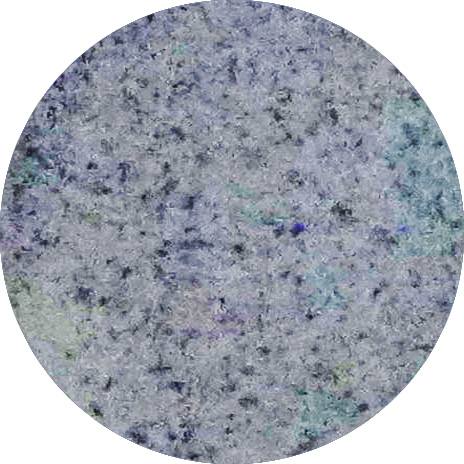

Supplement: Supplementary Figure 1 — MALAT1 was upregulated during osteogenic differentiation. **P<0.01. [file DataSheet_1.zip › MALAT1/ALP/Fig.1/NC.jpg]

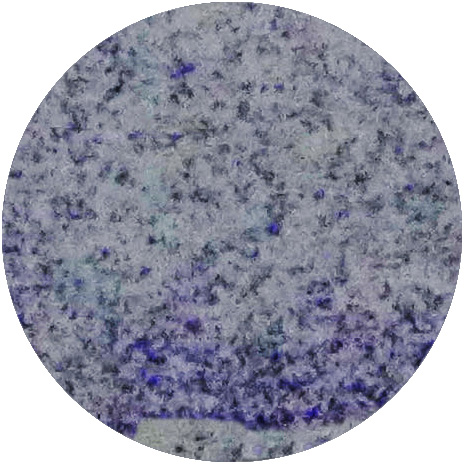

Supplement: Supplementary Figure 1 — MALAT1 was upregulated during osteogenic differentiation. **P<0.01. [file DataSheet_1.zip › MALAT1/ALP/Fig.2/control.jpg]

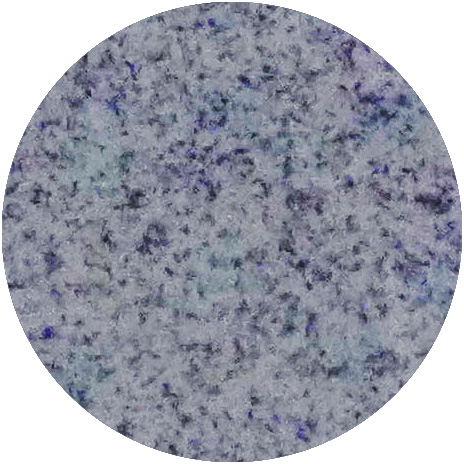

Supplement: Supplementary Figure 1 — MALAT1 was upregulated during osteogenic differentiation. **P<0.01. [file DataSheet_1.zip › MALAT1/ALP/Fig.2/MG+MALAT1.jpg]

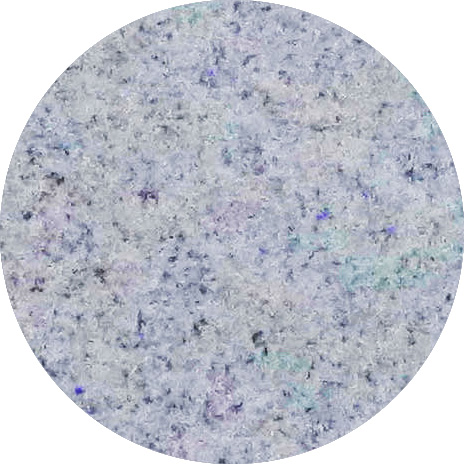

Supplement: Supplementary Figure 1 — MALAT1 was upregulated during osteogenic differentiation. **P<0.01. [file DataSheet_1.zip › MALAT1/ALP/Fig.2/MG+vector.jpg]

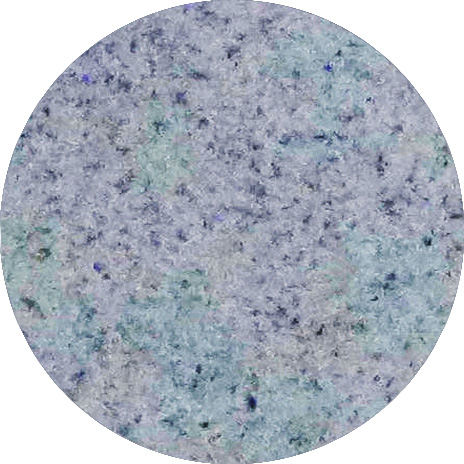

Supplement: Supplementary Figure 1 — MALAT1 was upregulated during osteogenic differentiation. **P<0.01. [file DataSheet_1.zip › MALAT1/ALP/Fig.2/MG.jpg]

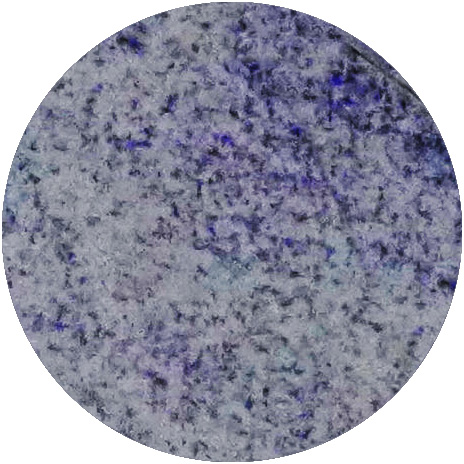

Supplement: Supplementary Figure 1 — MALAT1 was upregulated during osteogenic differentiation. **P<0.01. [file DataSheet_1.zip › MALAT1/ALP/Fig.5/control.jpg]

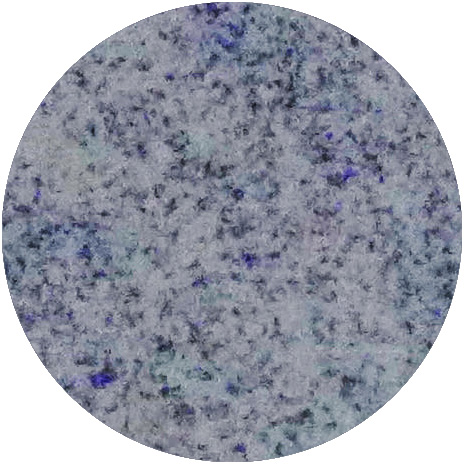

Supplement: Supplementary Figure 1 — MALAT1 was upregulated during osteogenic differentiation. **P<0.01. [file DataSheet_1.zip › MALAT1/ALP/Fig.5/MG+MALAT1+mimic NC.jpg]

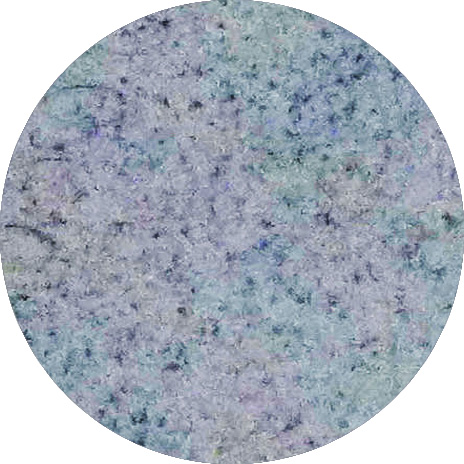

Supplement: Supplementary Figure 1 — MALAT1 was upregulated during osteogenic differentiation. **P<0.01. [file DataSheet_1.zip › MALAT1/ALP/Fig.5/MG+MALAT1+mimic.jpg]

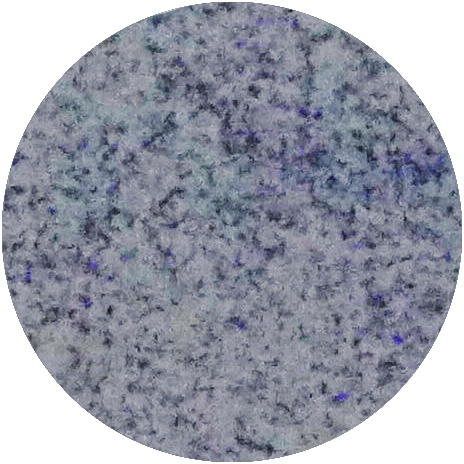

Supplement: Supplementary Figure 1 — MALAT1 was upregulated during osteogenic differentiation. **P<0.01. [file DataSheet_1.zip › MALAT1/ALP/Fig.5/MG+MALAT1.jpg]

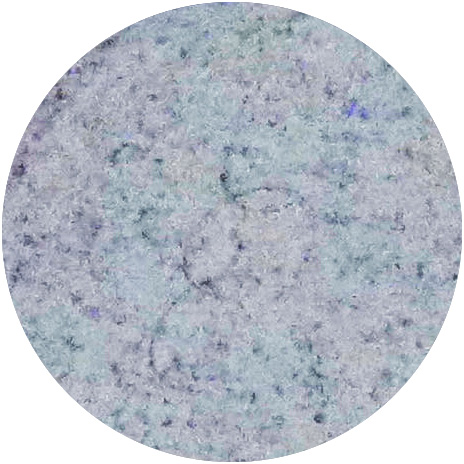

Supplement: Supplementary Figure 1 — MALAT1 was upregulated during osteogenic differentiation. **P<0.01. [file DataSheet_1.zip › MALAT1/ALP/Fig.5/MG.jpg]

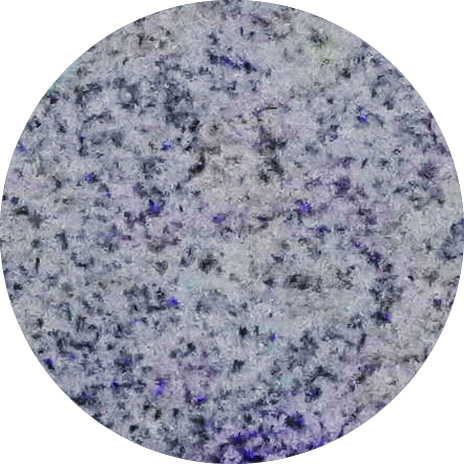

Supplement: Supplementary Figure 1 — MALAT1 was upregulated during osteogenic differentiation. **P<0.01. [file DataSheet_1.zip › MALAT1/ALP/Fig.7/control.jpg]

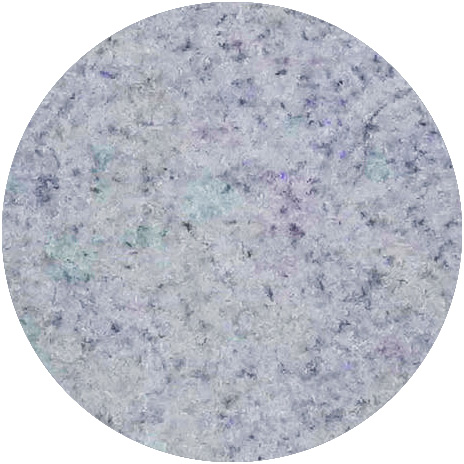

Supplement: Supplementary Figure 1 — MALAT1 was upregulated during osteogenic differentiation. **P<0.01. [file DataSheet_1.zip › MALAT1/ALP/Fig.7/MG+inhibitor NC.jpg]

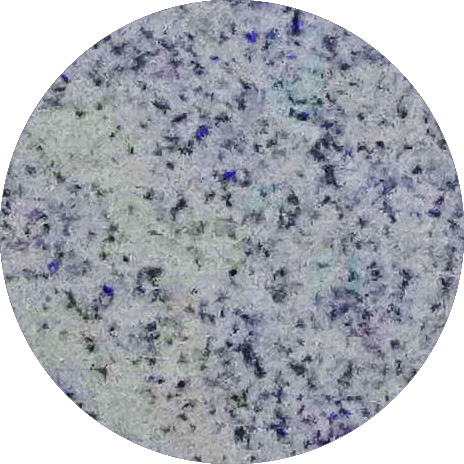

Supplement: Supplementary Figure 1 — MALAT1 was upregulated during osteogenic differentiation. **P<0.01. [file DataSheet_1.zip › MALAT1/ALP/Fig.7/MG+inhibitor+si-NC.jpg]

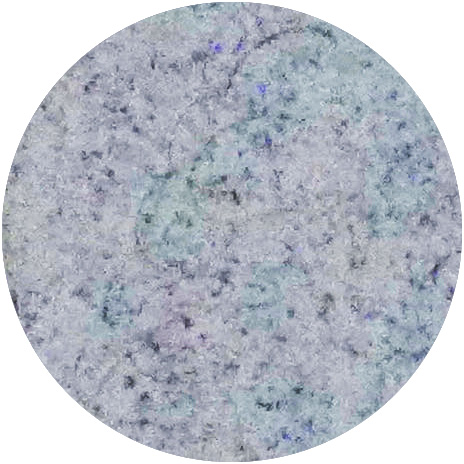

Supplement: Supplementary Figure 1 — MALAT1 was upregulated during osteogenic differentiation. **P<0.01. [file DataSheet_1.zip › MALAT1/ALP/Fig.7/MG+inhibitor+si-WNT7B.jpg]

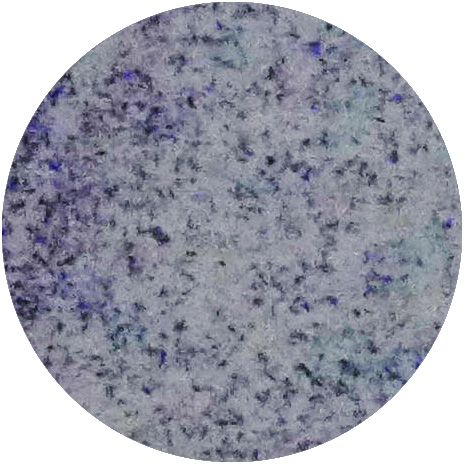

Supplement: Supplementary Figure 1 — MALAT1 was upregulated during osteogenic differentiation. **P<0.01. [file DataSheet_1.zip › MALAT1/ALP/Fig.7/MG+inhibitor.jpg]

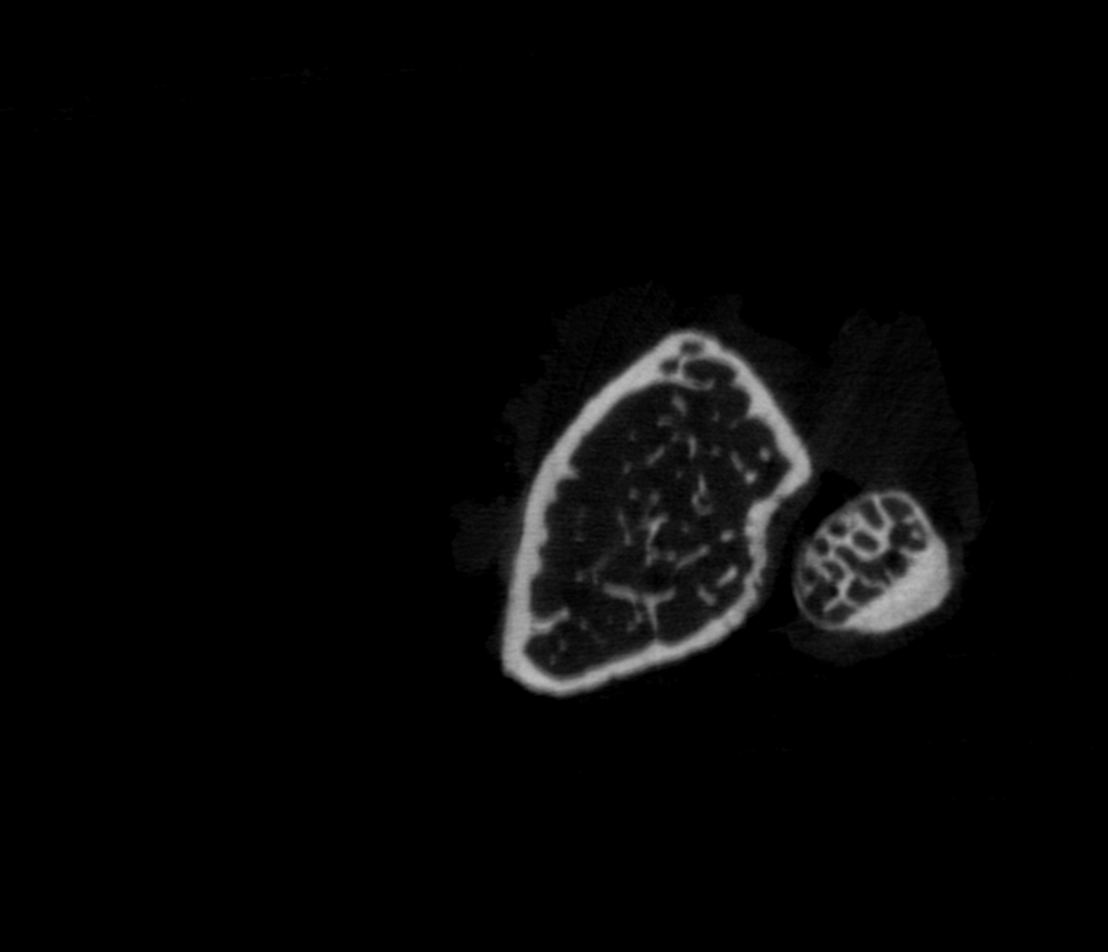

Supplement: Supplementary Figure 1 — MALAT1 was upregulated during osteogenic differentiation. **P<0.01. [file DataSheet_1.zip › MALAT1/CT/CT1.png]

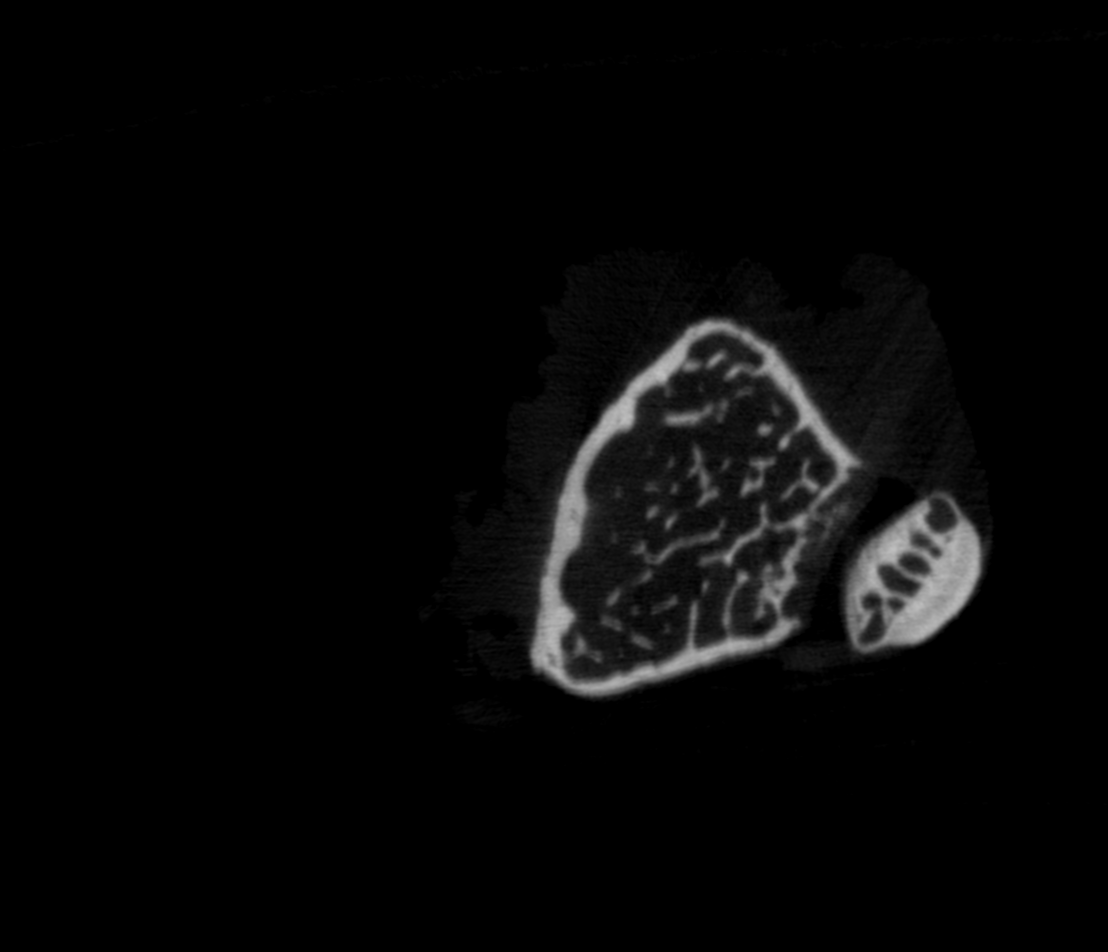

Supplement: Supplementary Figure 1 — MALAT1 was upregulated during osteogenic differentiation. **P<0.01. [file DataSheet_1.zip › MALAT1/CT/CT2.png]

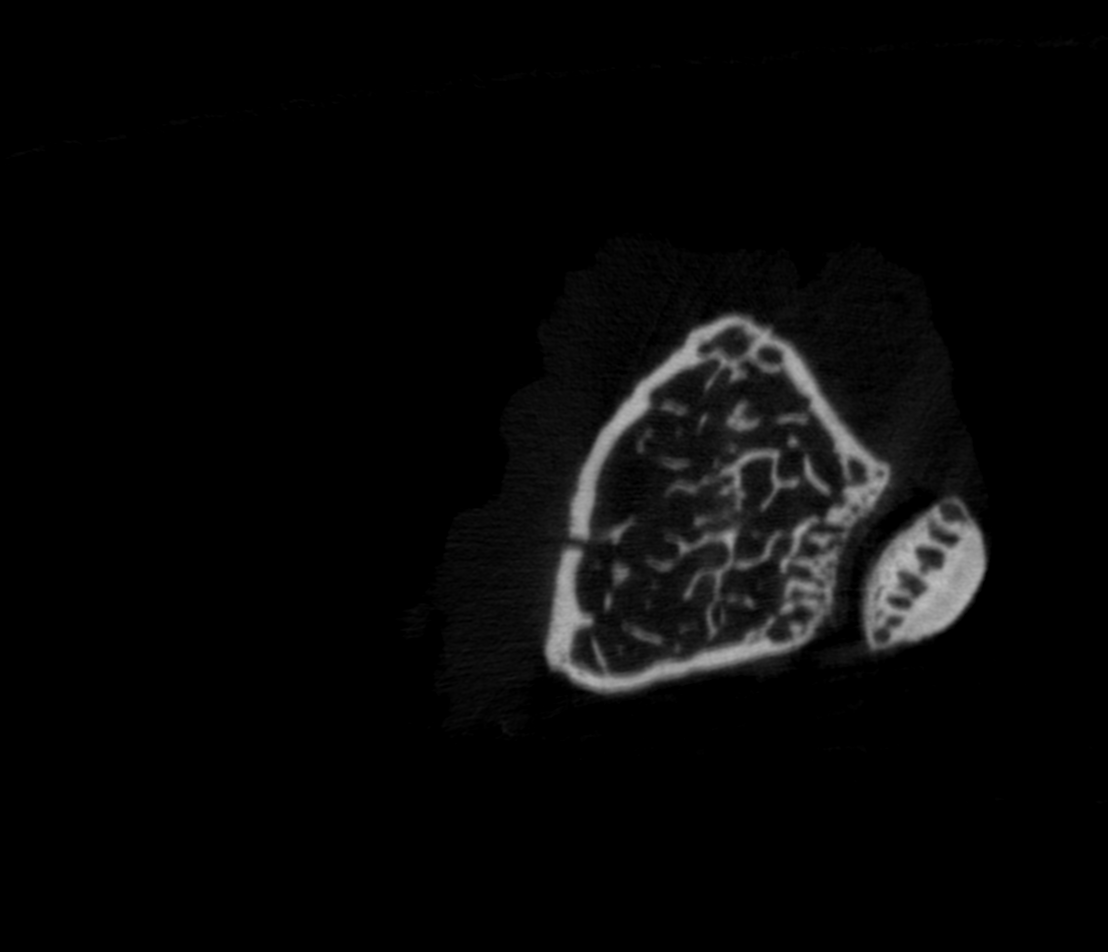

Supplement: Supplementary Figure 1 — MALAT1 was upregulated during osteogenic differentiation. **P<0.01. [file DataSheet_1.zip › MALAT1/CT/CT3.png]

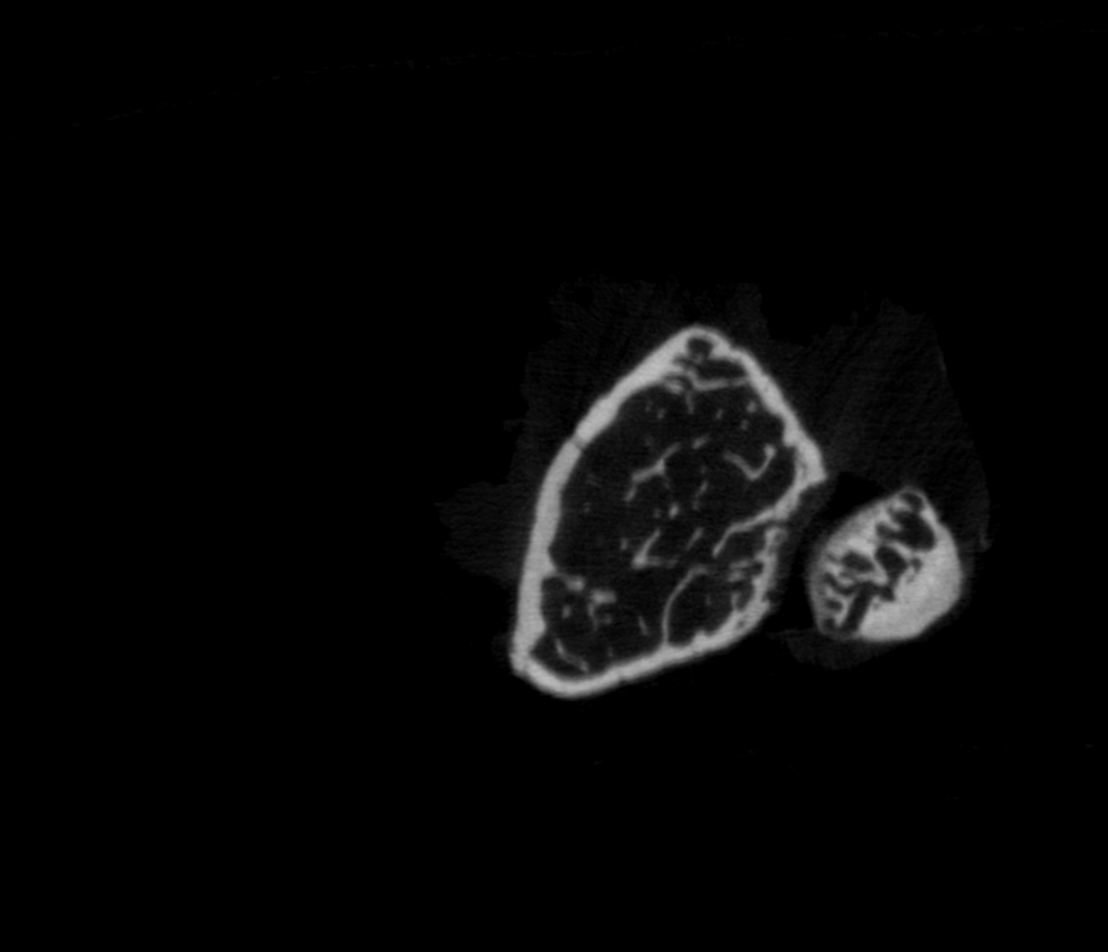

Supplement: Supplementary Figure 1 — MALAT1 was upregulated during osteogenic differentiation. **P<0.01. [file DataSheet_1.zip › MALAT1/CT/CT4.png]

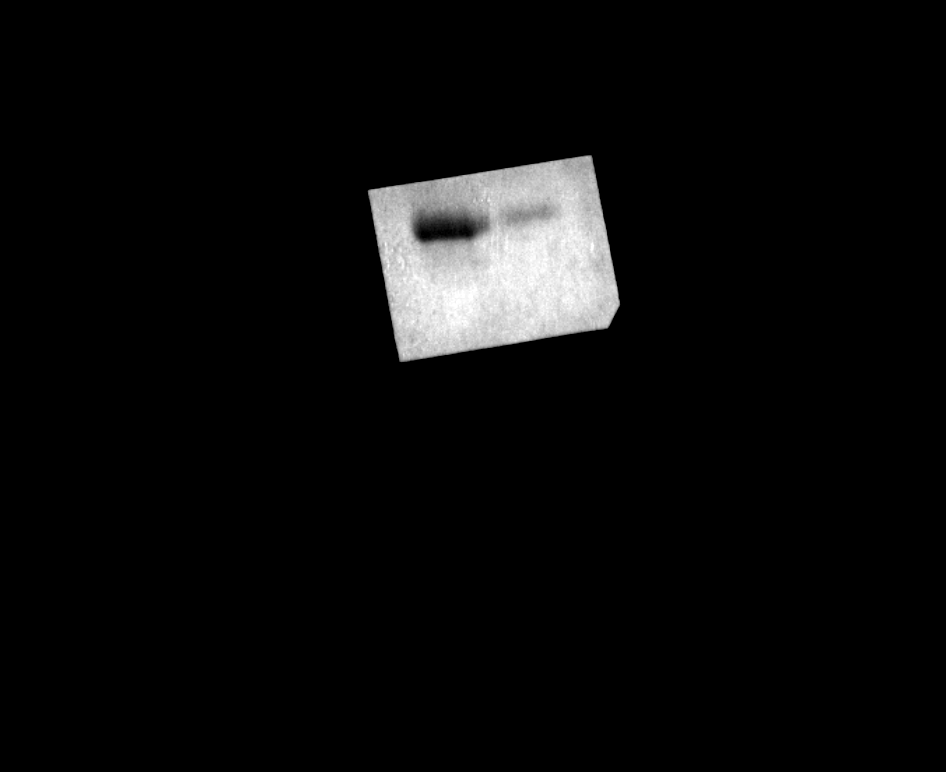

Supplement: Supplementary Figure 1 — MALAT1 was upregulated during osteogenic differentiation. **P<0.01. [file DataSheet_1.zip › MALAT1/WB/Fig.1/Bglap.tif]

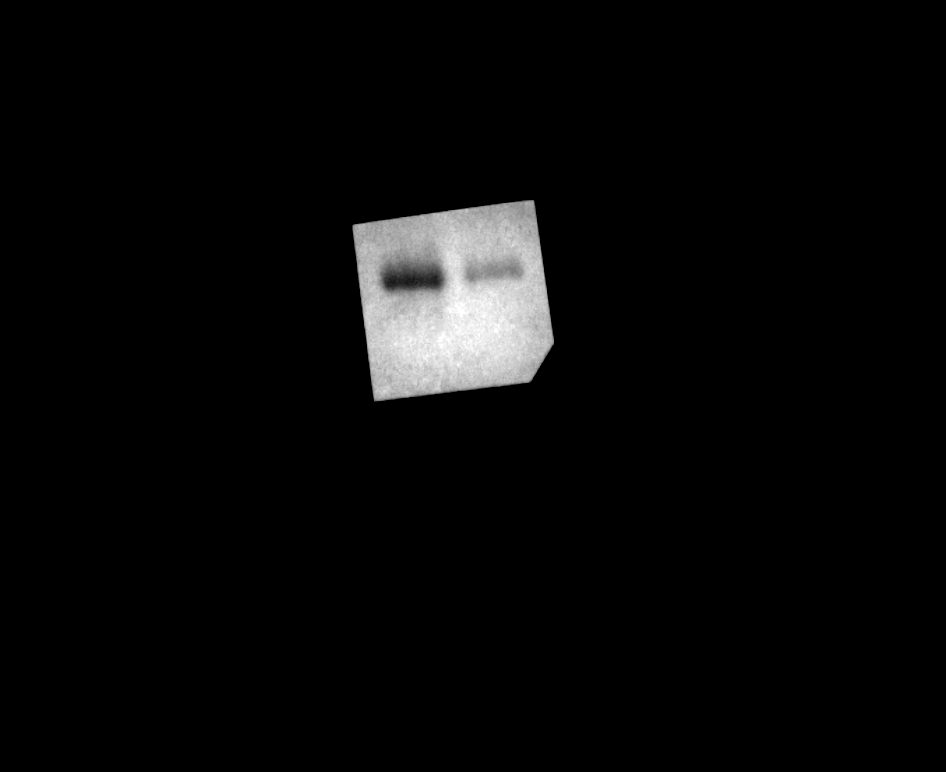

Supplement: Supplementary Figure 1 — MALAT1 was upregulated during osteogenic differentiation. **P<0.01. [file DataSheet_1.zip › MALAT1/WB/Fig.1/Col1a1.tif]

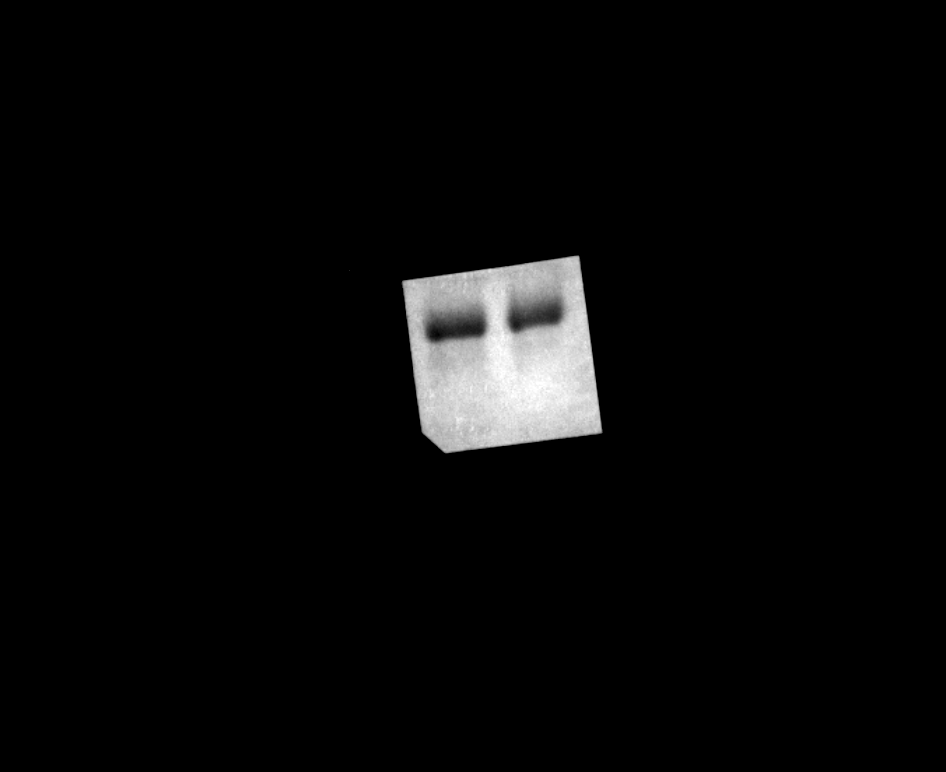

Supplement: Supplementary Figure 1 — MALAT1 was upregulated during osteogenic differentiation. **P<0.01. [file DataSheet_1.zip › MALAT1/WB/Fig.1/GAPDH.tif]

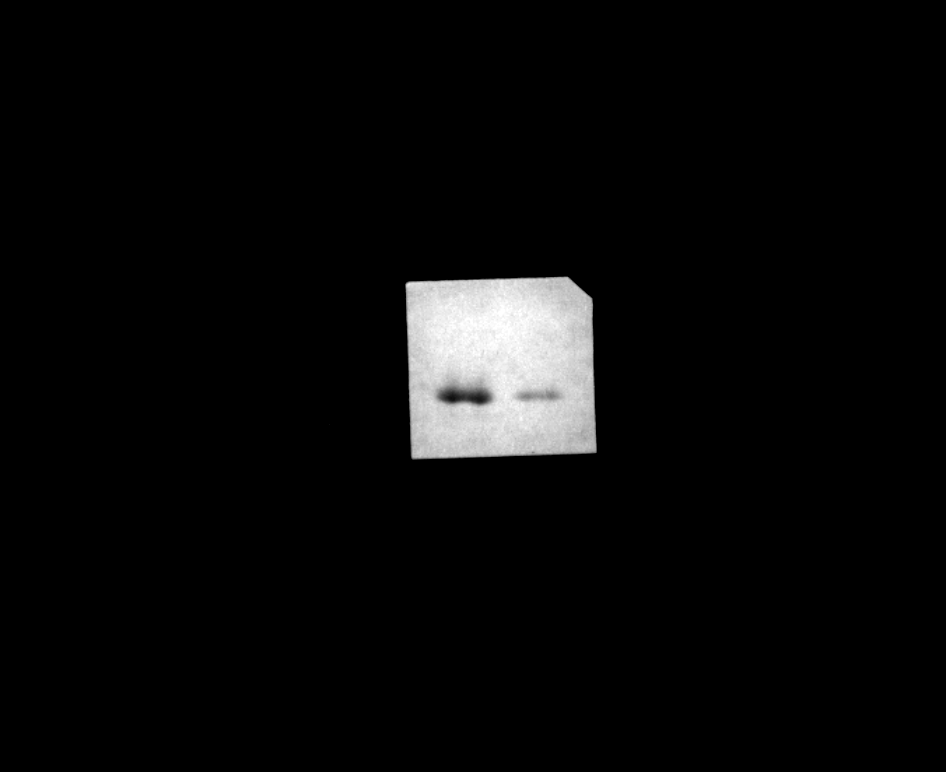

Supplement: Supplementary Figure 1 — MALAT1 was upregulated during osteogenic differentiation. **P<0.01. [file DataSheet_1.zip › MALAT1/WB/Fig.1/Runx2.tif]

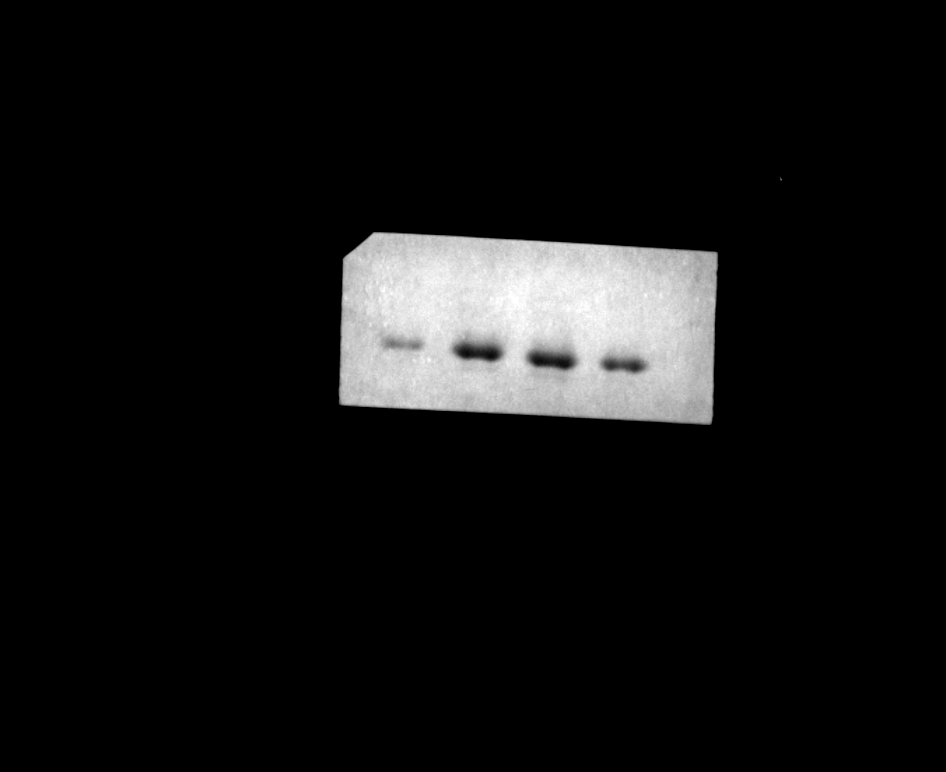

Supplement: Supplementary Figure 1 — MALAT1 was upregulated during osteogenic differentiation. **P<0.01. [file DataSheet_1.zip › MALAT1/WB/Fig.2/Bax.tif]

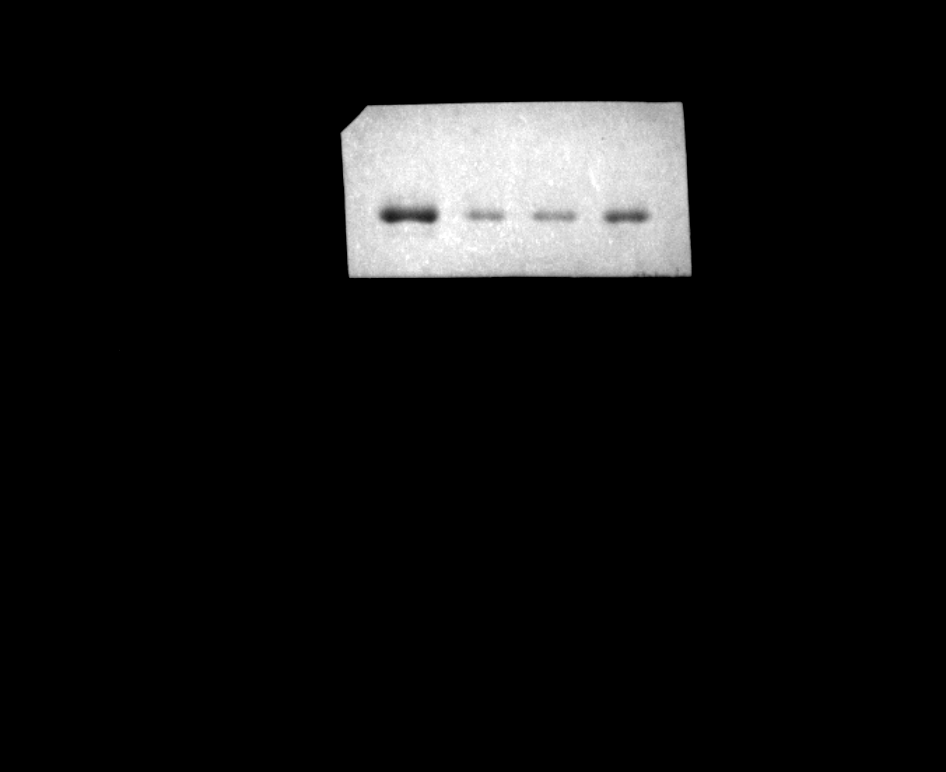

Supplement: Supplementary Figure 1 — MALAT1 was upregulated during osteogenic differentiation. **P<0.01. [file DataSheet_1.zip › MALAT1/WB/Fig.2/Bcl-2.tif]

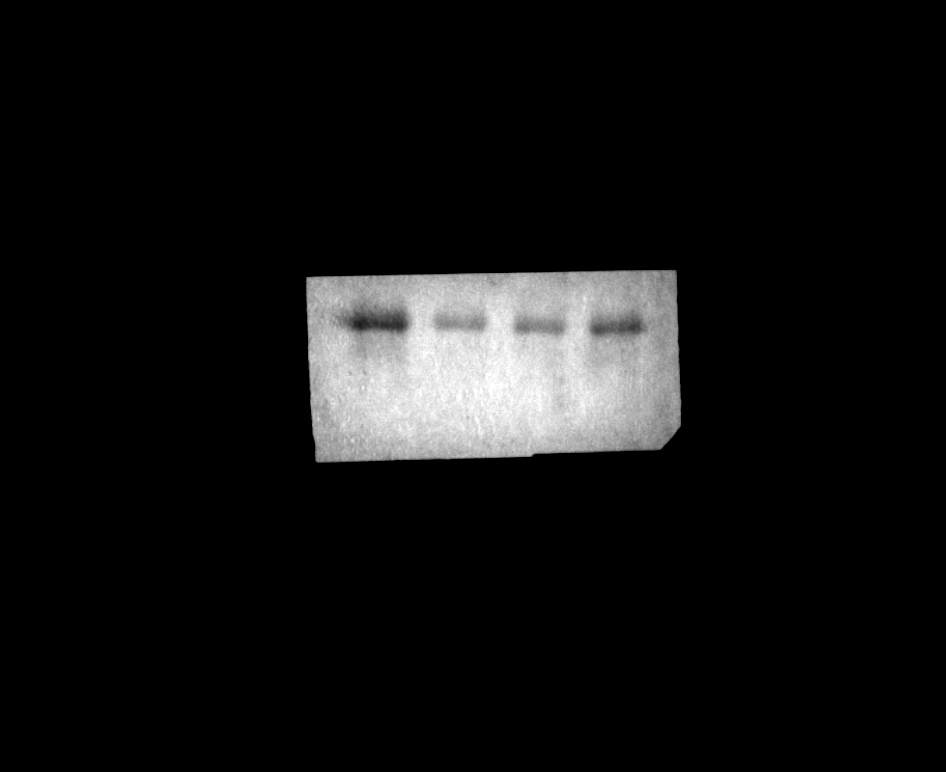

Supplement: Supplementary Figure 1 — MALAT1 was upregulated during osteogenic differentiation. **P<0.01. [file DataSheet_1.zip › MALAT1/WB/Fig.2/Bglap.tif]

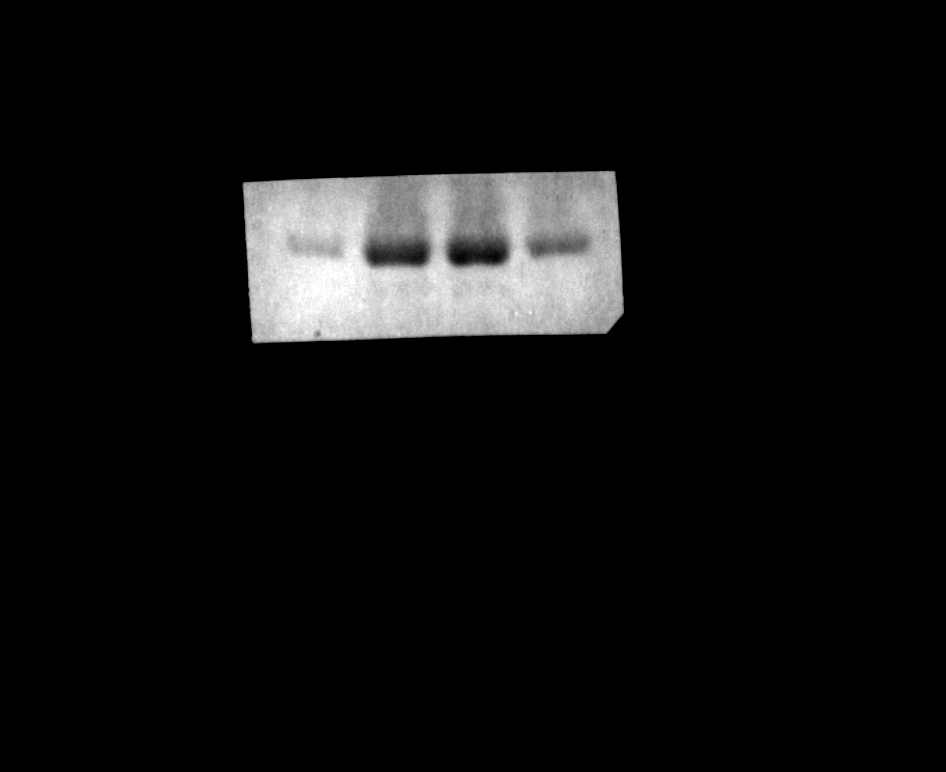

Supplement: Supplementary Figure 1 — MALAT1 was upregulated during osteogenic differentiation. **P<0.01. [file DataSheet_1.zip › MALAT1/WB/Fig.2/Caspase-3.tif]

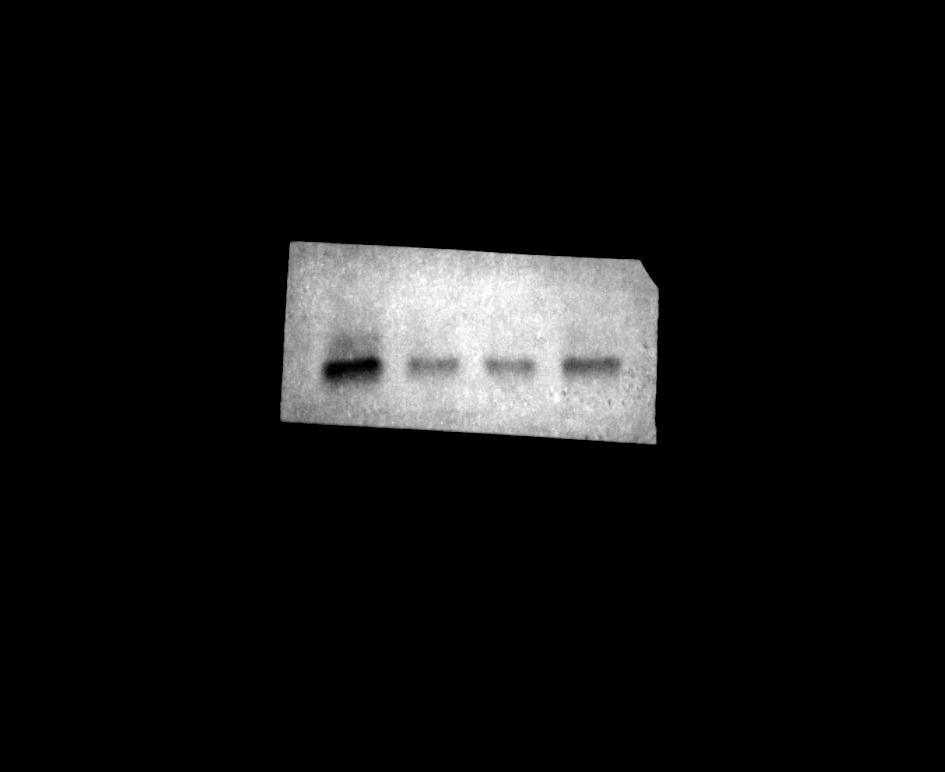

Supplement: Supplementary Figure 1 — MALAT1 was upregulated during osteogenic differentiation. **P<0.01. [file DataSheet_1.zip › MALAT1/WB/Fig.2/Col1a1.tif]

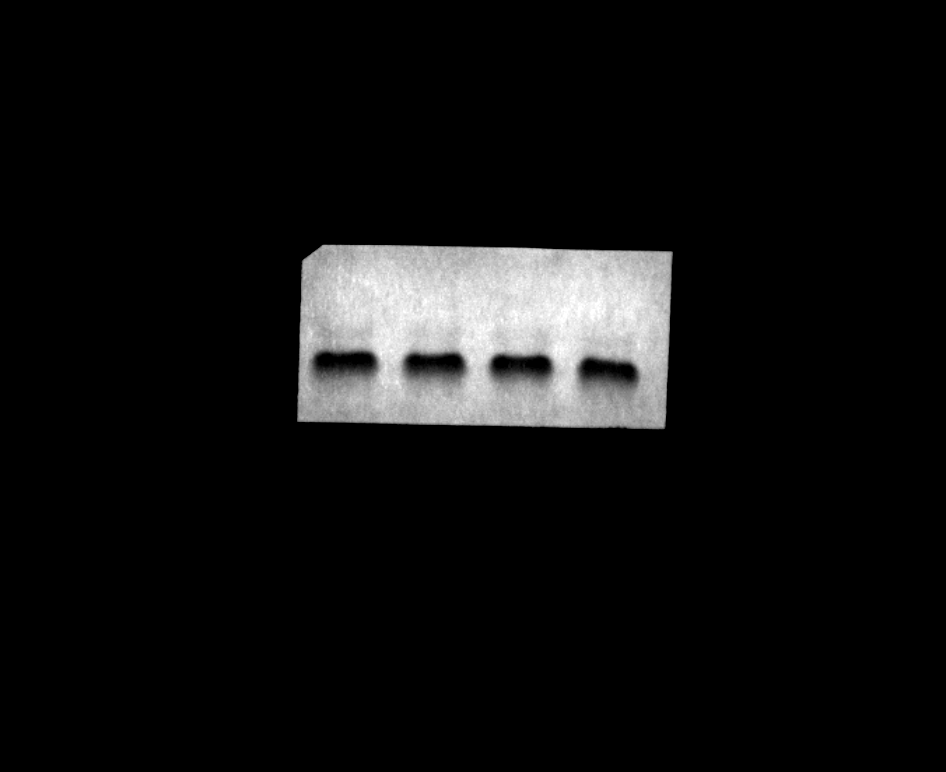

Supplement: Supplementary Figure 1 — MALAT1 was upregulated during osteogenic differentiation. **P<0.01. [file DataSheet_1.zip › MALAT1/WB/Fig.2/GAPDH (1).tif]

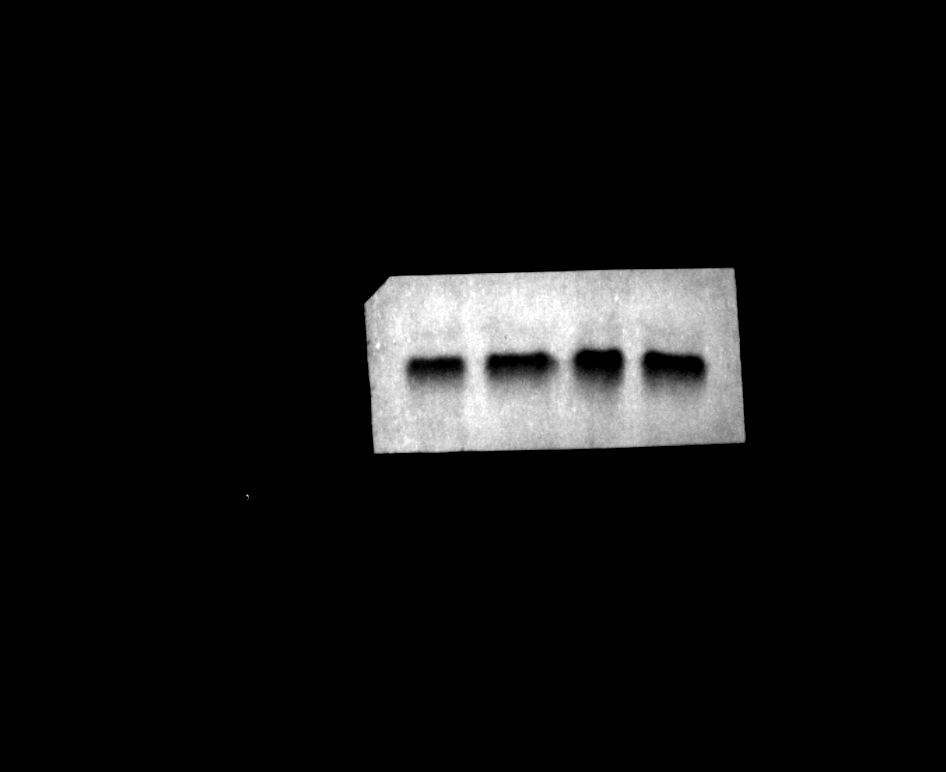

Supplement: Supplementary Figure 1 — MALAT1 was upregulated during osteogenic differentiation. **P<0.01. [file DataSheet_1.zip › MALAT1/WB/Fig.2/GAPDH (2).tif]

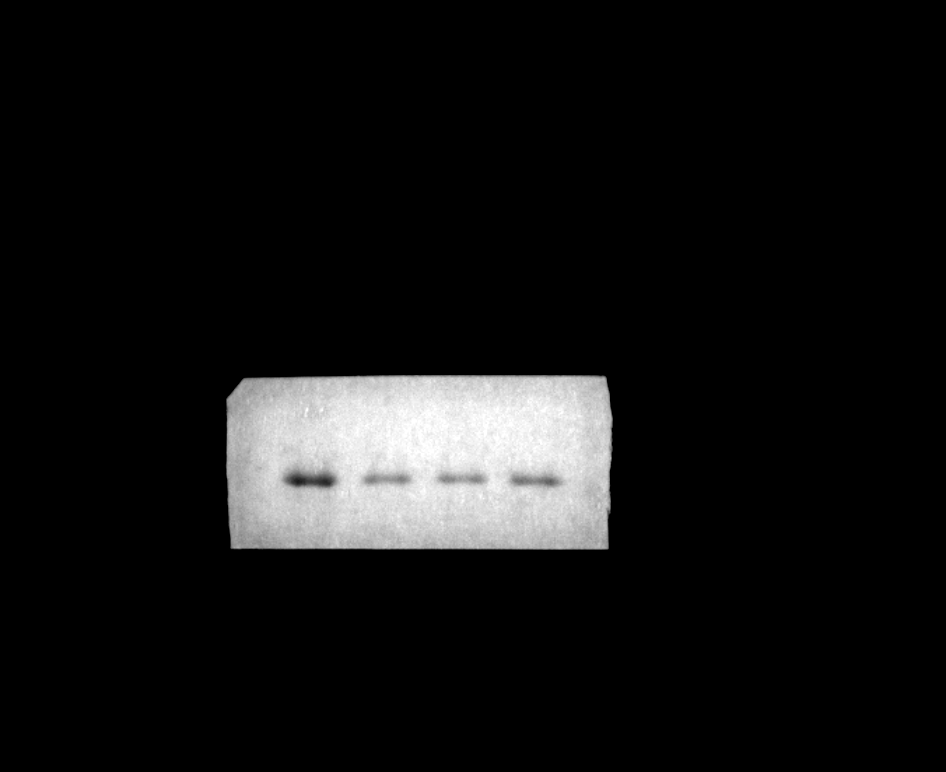

Supplement: Supplementary Figure 1 — MALAT1 was upregulated during osteogenic differentiation. **P<0.01. [file DataSheet_1.zip › MALAT1/WB/Fig.2/Runx2.tif]

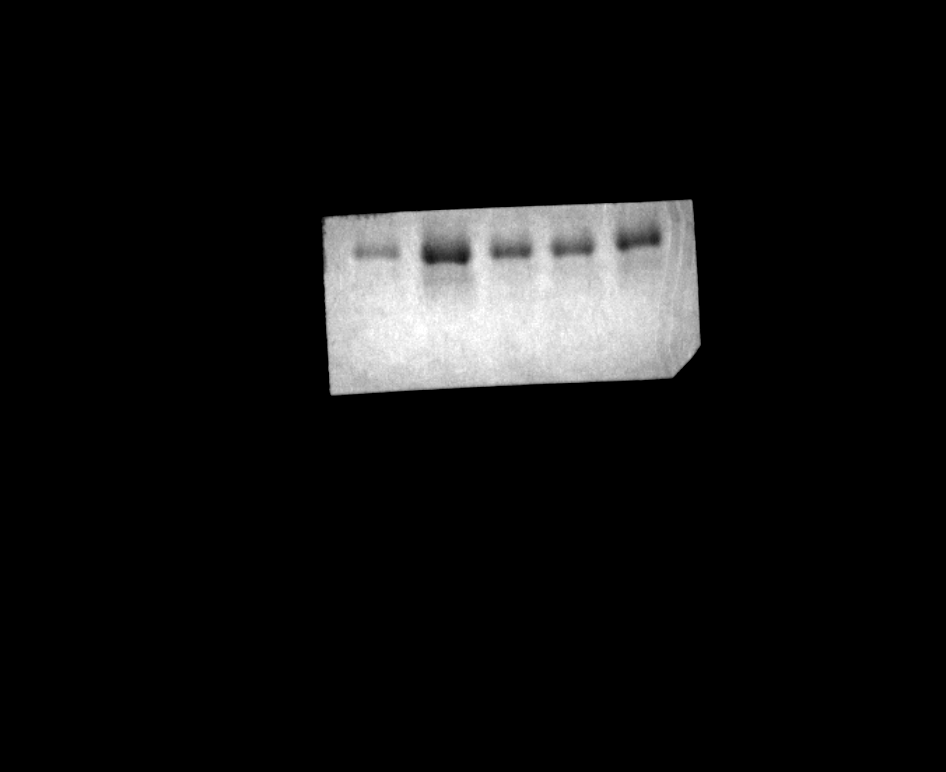

Supplement: Supplementary Figure 1 — MALAT1 was upregulated during osteogenic differentiation. **P<0.01. [file DataSheet_1.zip › MALAT1/WB/Fig.5/BAX.tif]

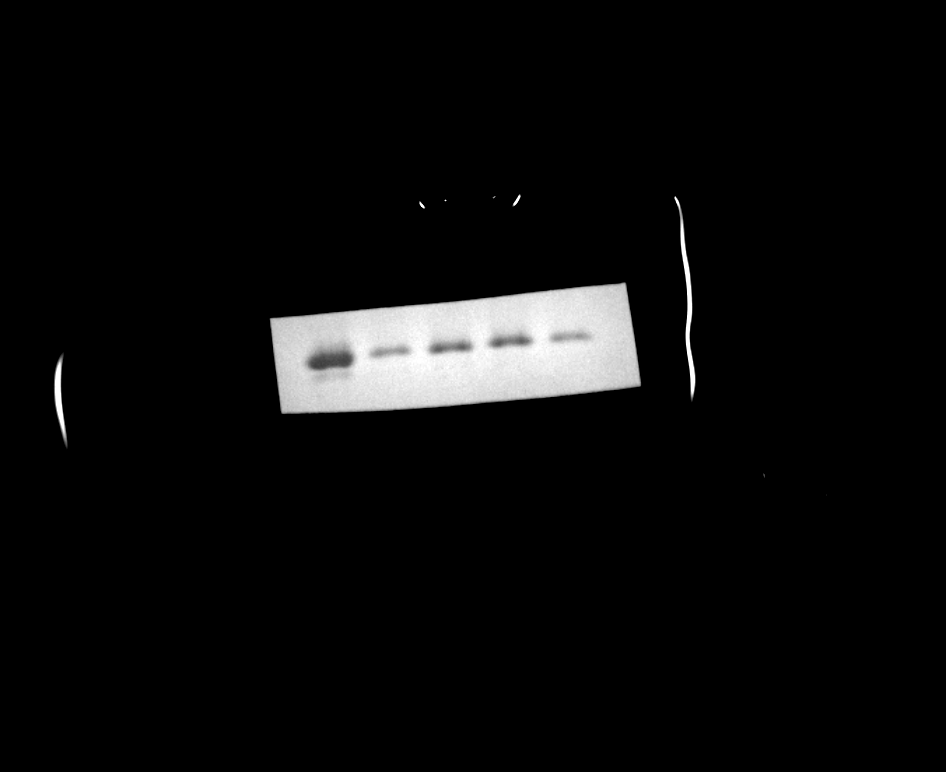

Supplement: Supplementary Figure 1 — MALAT1 was upregulated during osteogenic differentiation. **P<0.01. [file DataSheet_1.zip › MALAT1/WB/Fig.5/Bcl-2.tif]

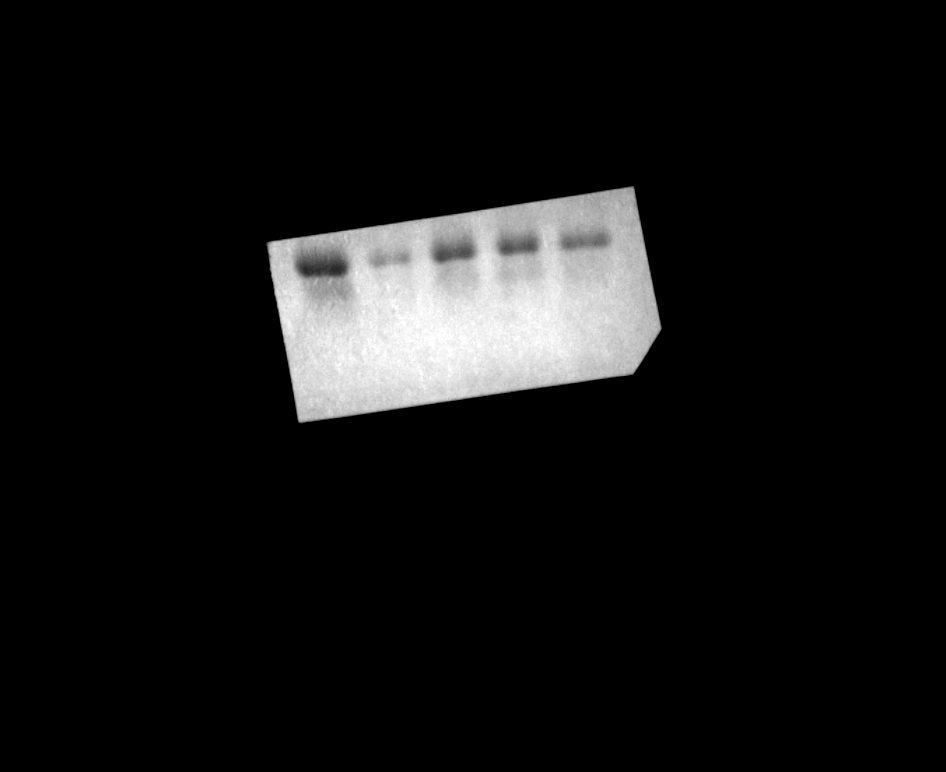

Supplement: Supplementary Figure 1 — MALAT1 was upregulated during osteogenic differentiation. **P<0.01. [file DataSheet_1.zip › MALAT1/WB/Fig.5/Bglap.tif]

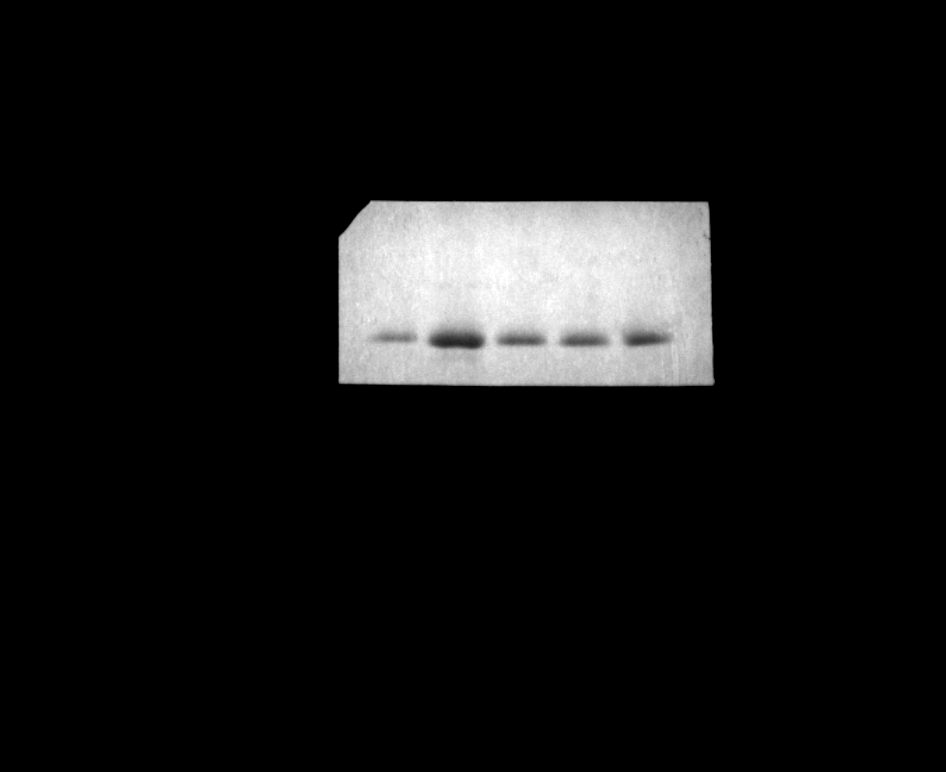

Supplement: Supplementary Figure 1 — MALAT1 was upregulated during osteogenic differentiation. **P<0.01. [file DataSheet_1.zip › MALAT1/WB/Fig.5/Caspase-3.tif]

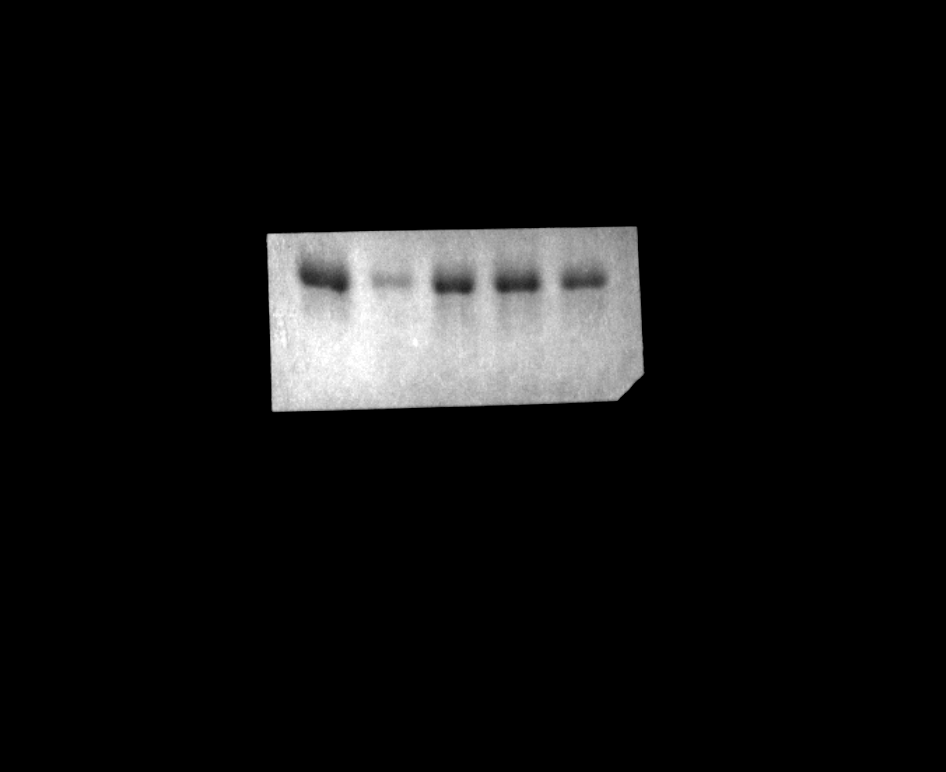

Supplement: Supplementary Figure 1 — MALAT1 was upregulated during osteogenic differentiation. **P<0.01. [file DataSheet_1.zip › MALAT1/WB/Fig.5/Col1a1.tif]

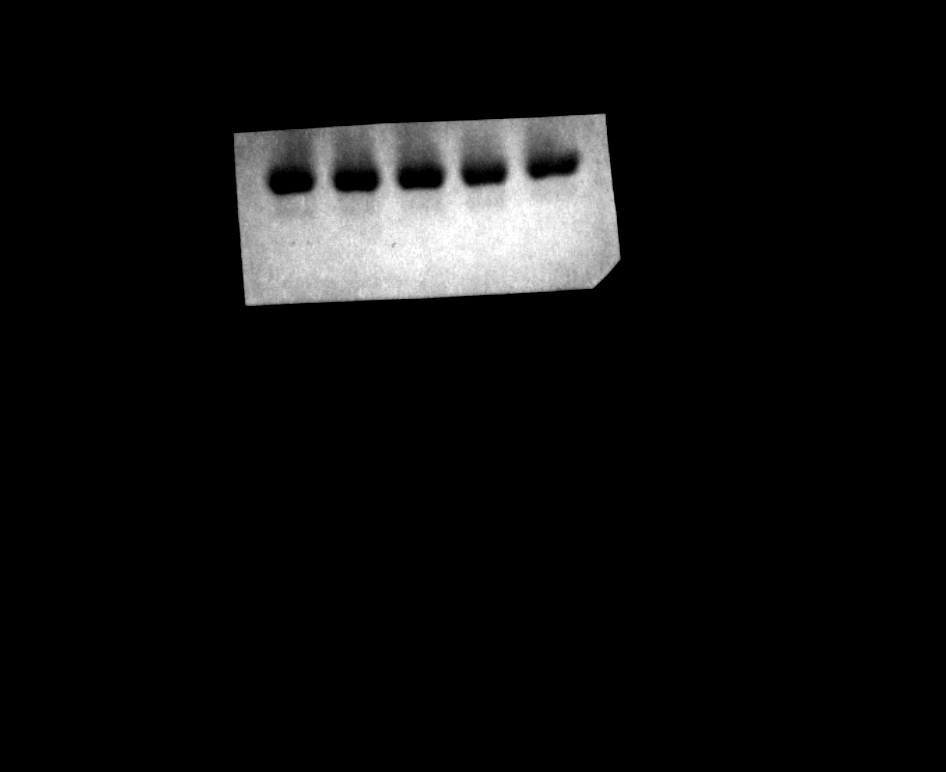

Supplement: Supplementary Figure 1 — MALAT1 was upregulated during osteogenic differentiation. **P<0.01. [file DataSheet_1.zip › MALAT1/WB/Fig.5/GAPDH (1).tif]

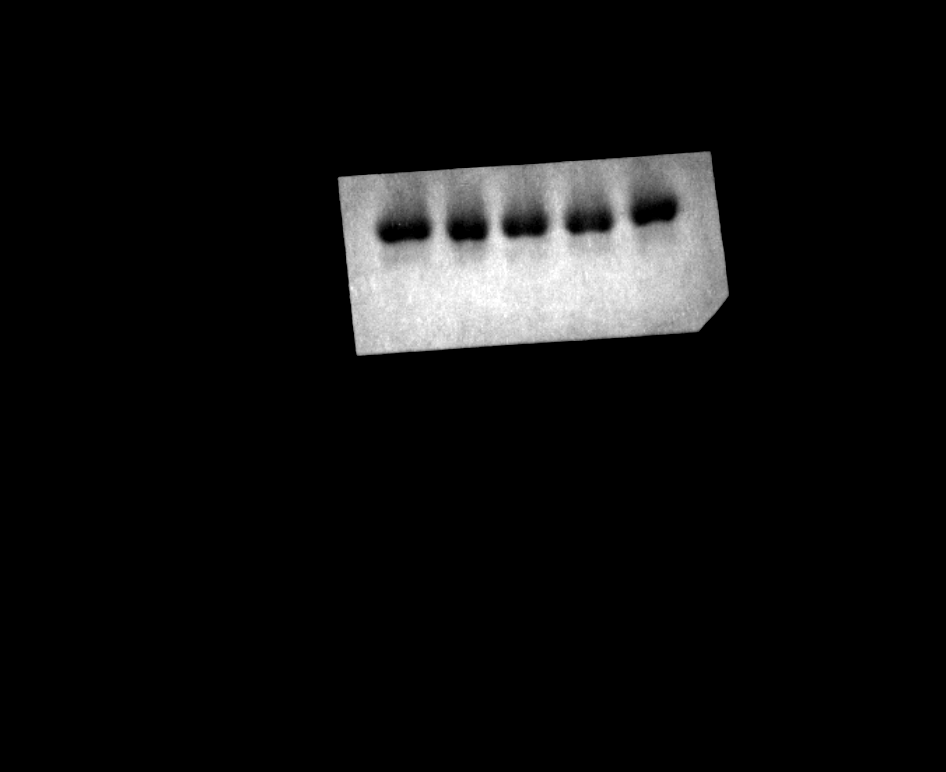

Supplement: Supplementary Figure 1 — MALAT1 was upregulated during osteogenic differentiation. **P<0.01. [file DataSheet_1.zip › MALAT1/WB/Fig.5/GAPDH (2).tif]

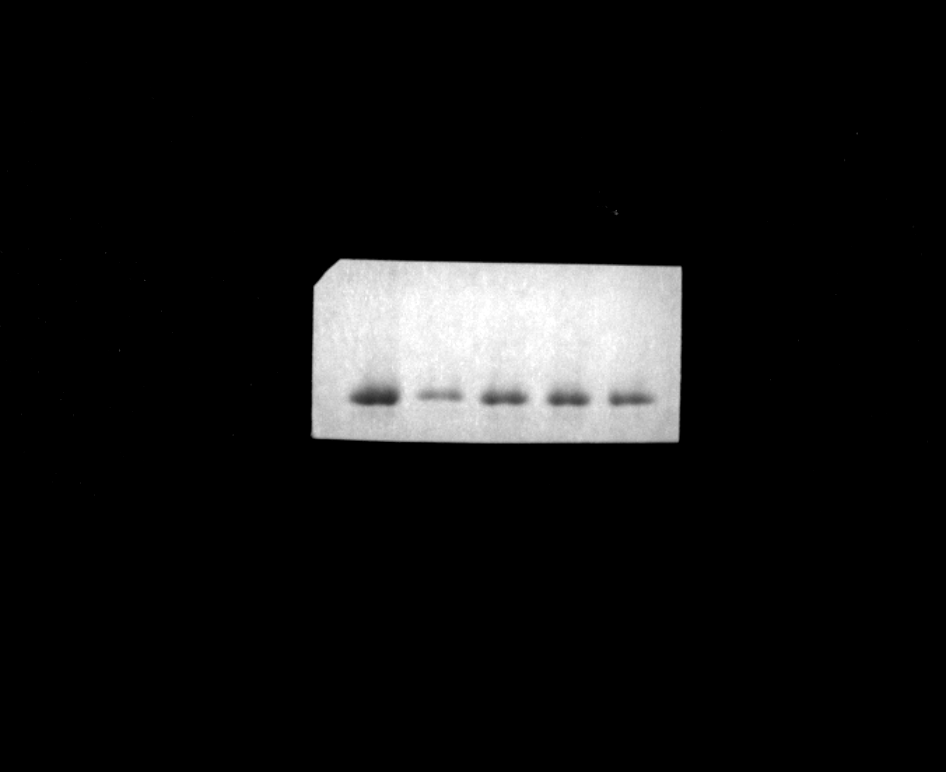

Supplement: Supplementary Figure 1 — MALAT1 was upregulated during osteogenic differentiation. **P<0.01. [file DataSheet_1.zip › MALAT1/WB/Fig.5/Runx2.tif]

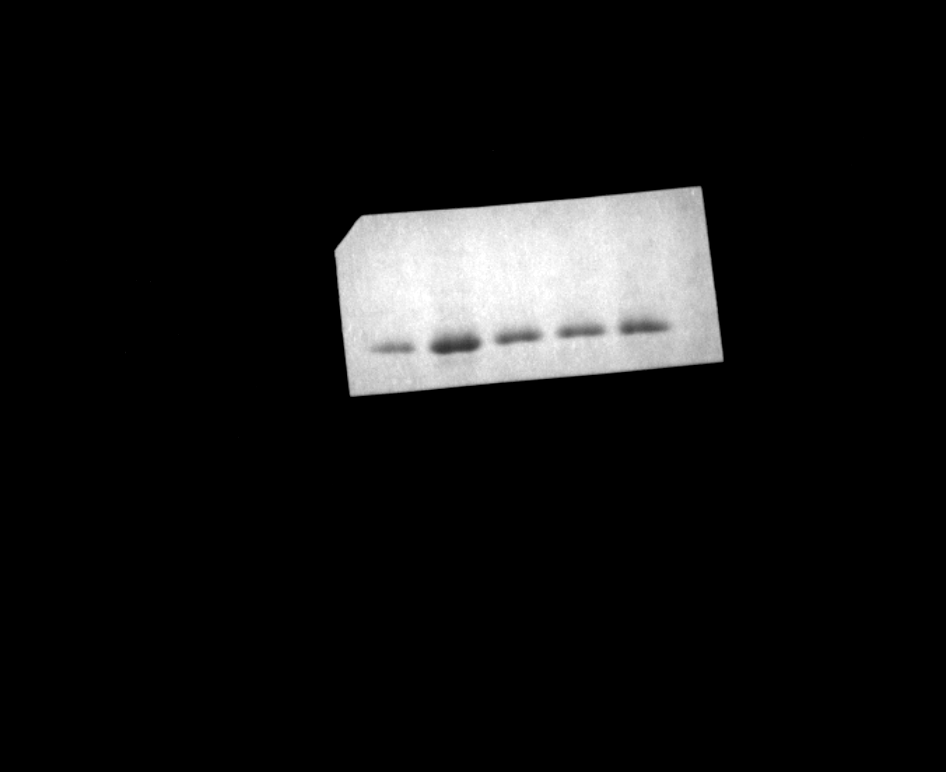

Supplement: Supplementary Figure 1 — MALAT1 was upregulated during osteogenic differentiation. **P<0.01. [file DataSheet_1.zip › MALAT1/WB/Fig.7/Bax.tif]

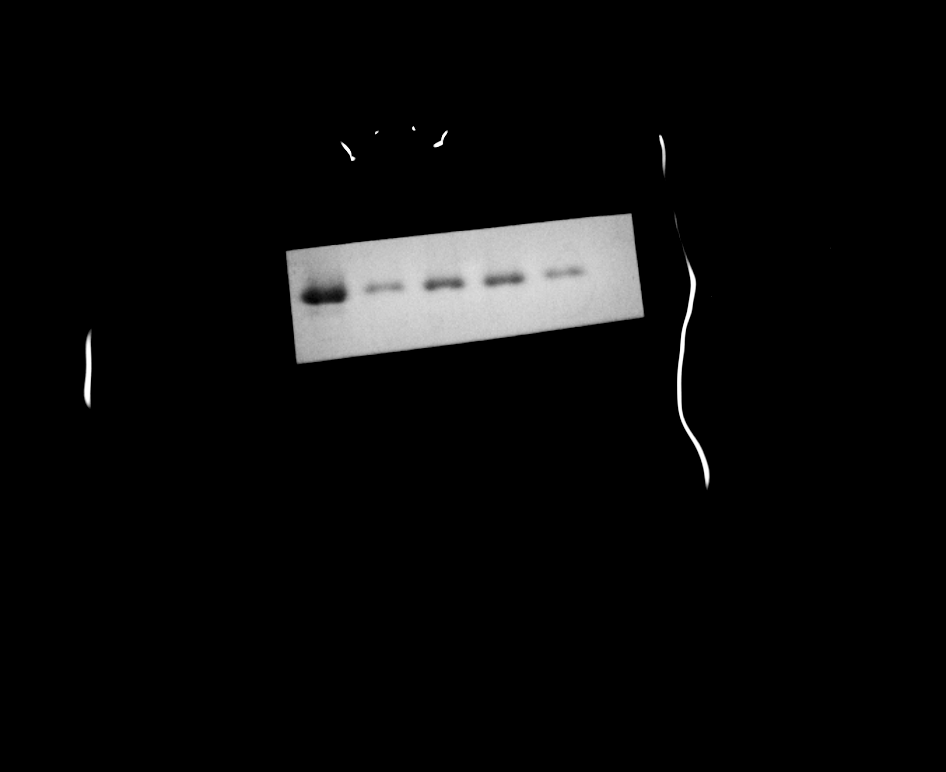

Supplement: Supplementary Figure 1 — MALAT1 was upregulated during osteogenic differentiation. **P<0.01. [file DataSheet_1.zip › MALAT1/WB/Fig.7/Bcl-2.tif]

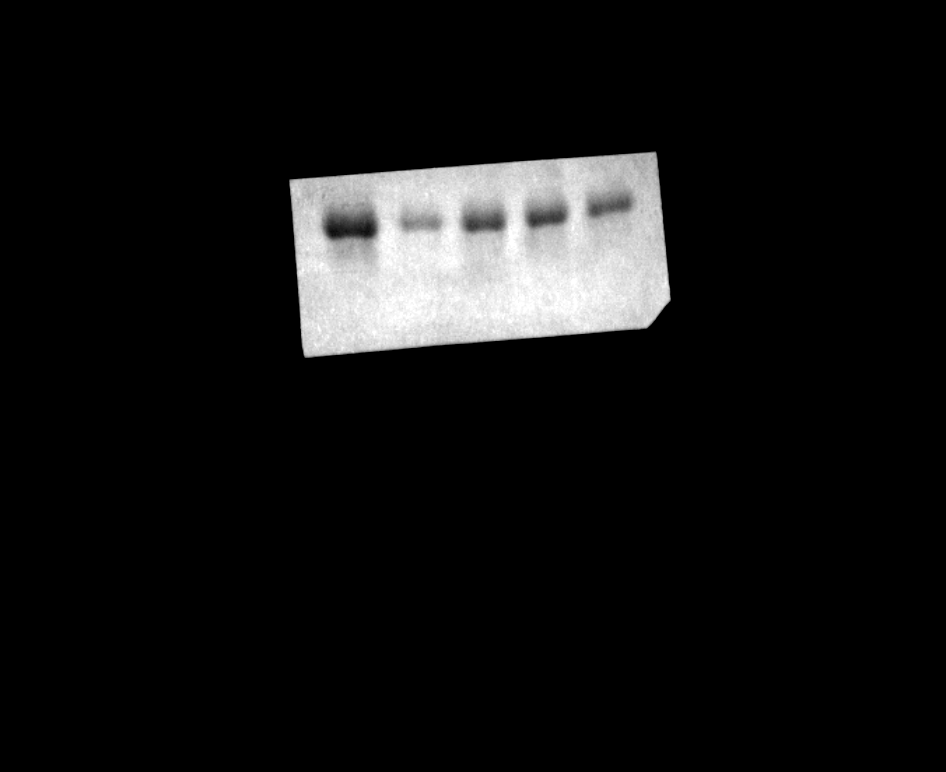

Supplement: Supplementary Figure 1 — MALAT1 was upregulated during osteogenic differentiation. **P<0.01. [file DataSheet_1.zip › MALAT1/WB/Fig.7/Bglap.tif]

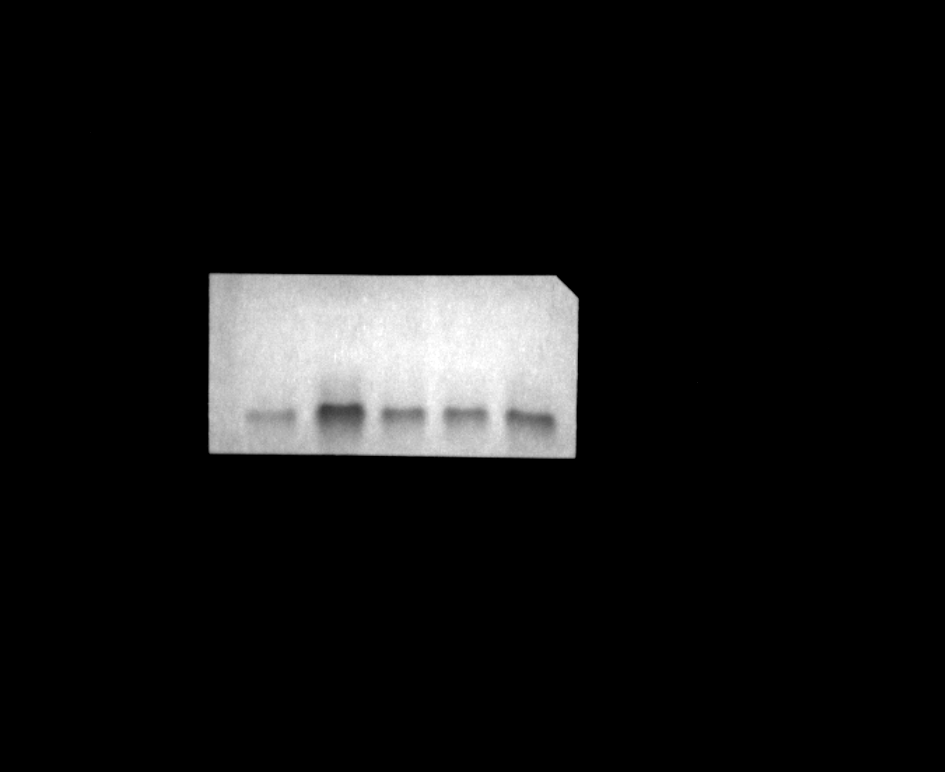

Supplement: Supplementary Figure 1 — MALAT1 was upregulated during osteogenic differentiation. **P<0.01. [file DataSheet_1.zip › MALAT1/WB/Fig.7/Caspase-3.tif]

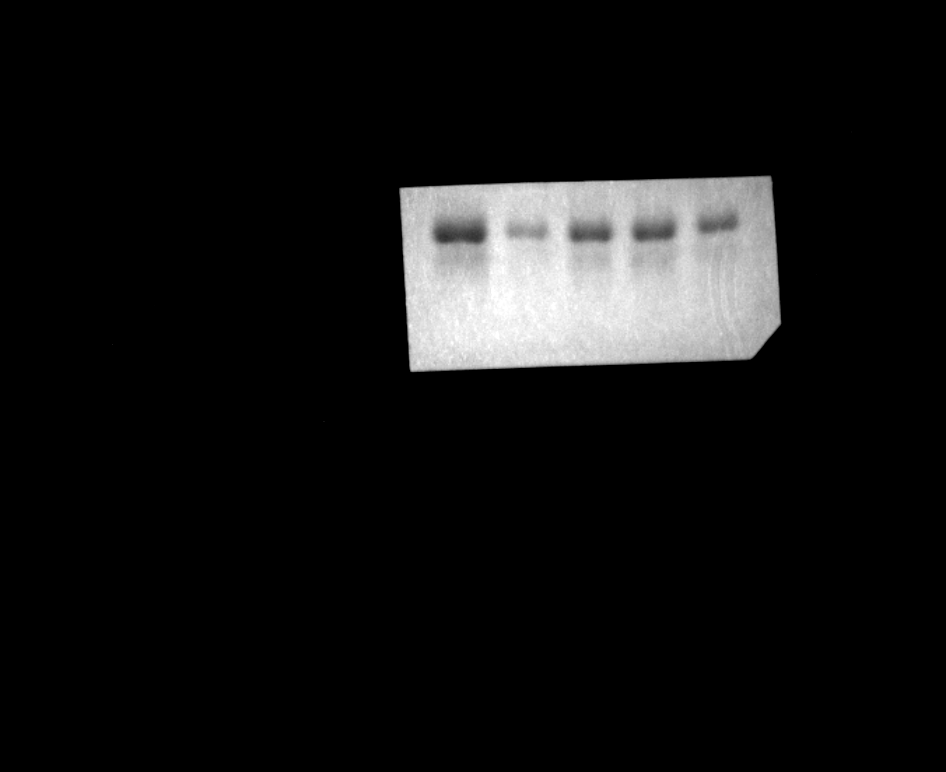

Supplement: Supplementary Figure 1 — MALAT1 was upregulated during osteogenic differentiation. **P<0.01. [file DataSheet_1.zip › MALAT1/WB/Fig.7/Col1a1.tif]

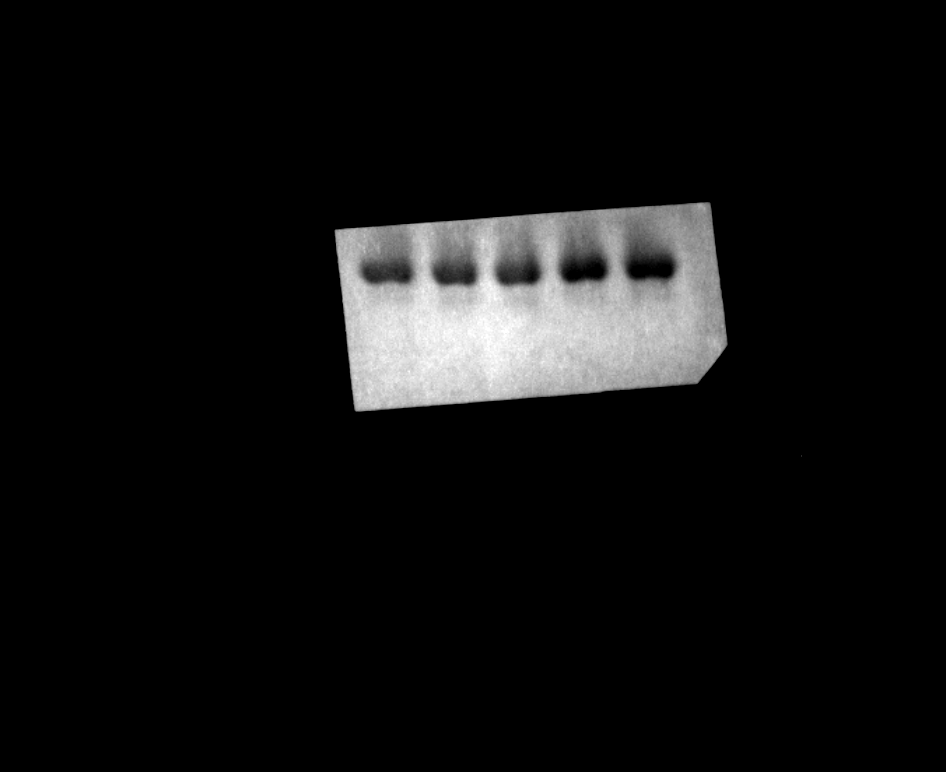

Supplement: Supplementary Figure 1 — MALAT1 was upregulated during osteogenic differentiation. **P<0.01. [file DataSheet_1.zip › MALAT1/WB/Fig.7/GAPDH (1).tif]

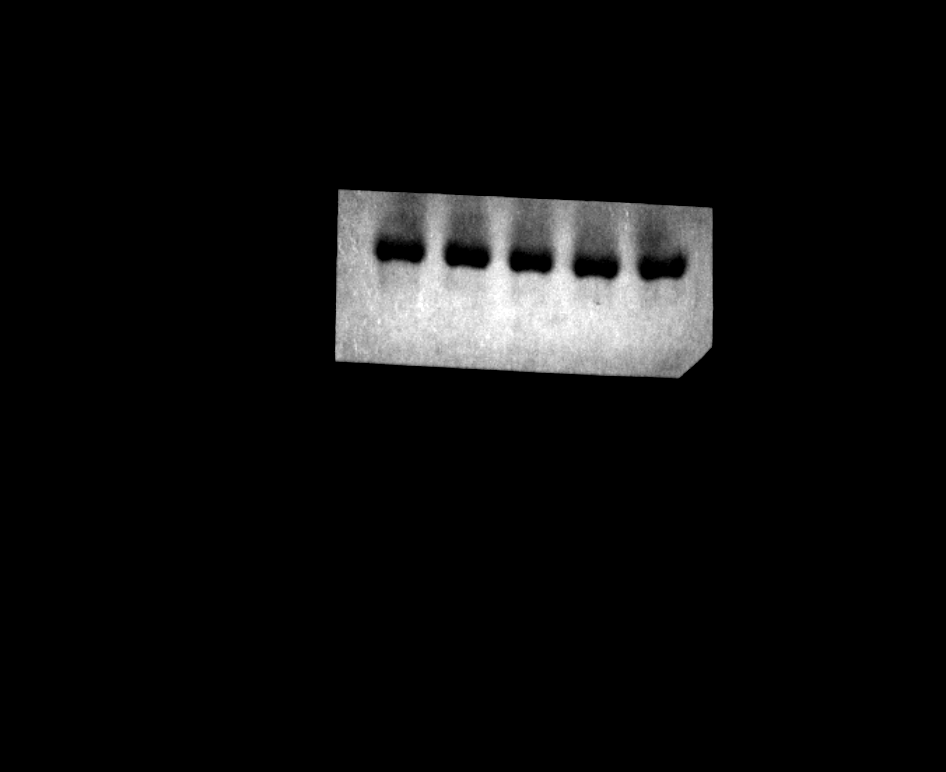

Supplement: Supplementary Figure 1 — MALAT1 was upregulated during osteogenic differentiation. **P<0.01. [file DataSheet_1.zip › MALAT1/WB/Fig.7/GAPDH (2).tif]

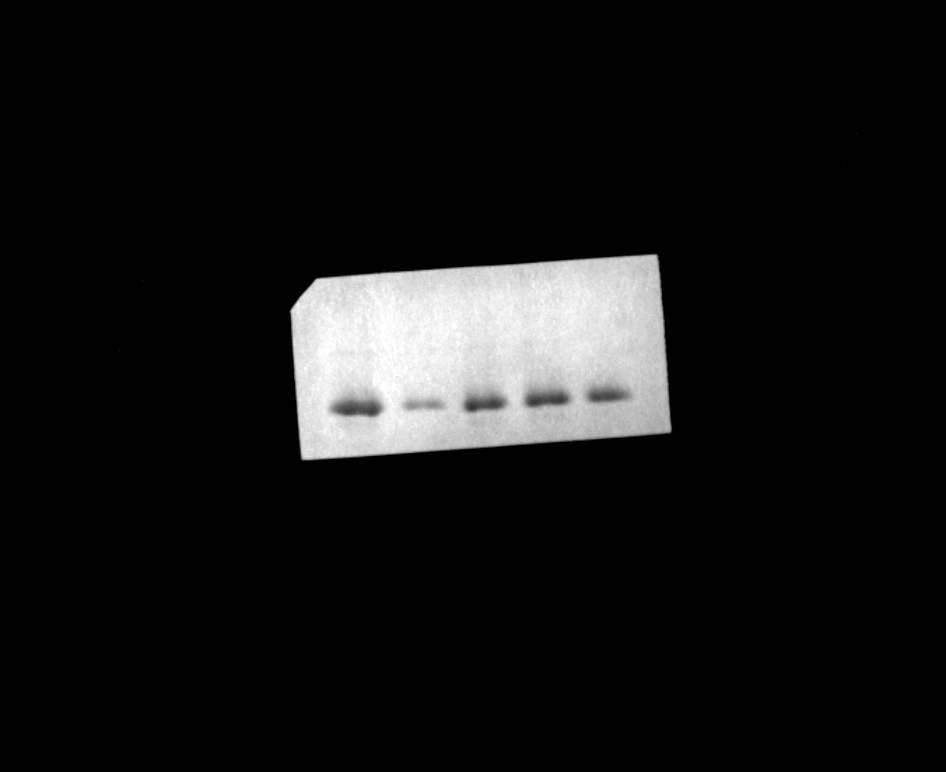

Supplement: Supplementary Figure 1 — MALAT1 was upregulated during osteogenic differentiation. **P<0.01. [file DataSheet_1.zip › MALAT1/WB/Fig.7/Runx2.tif]

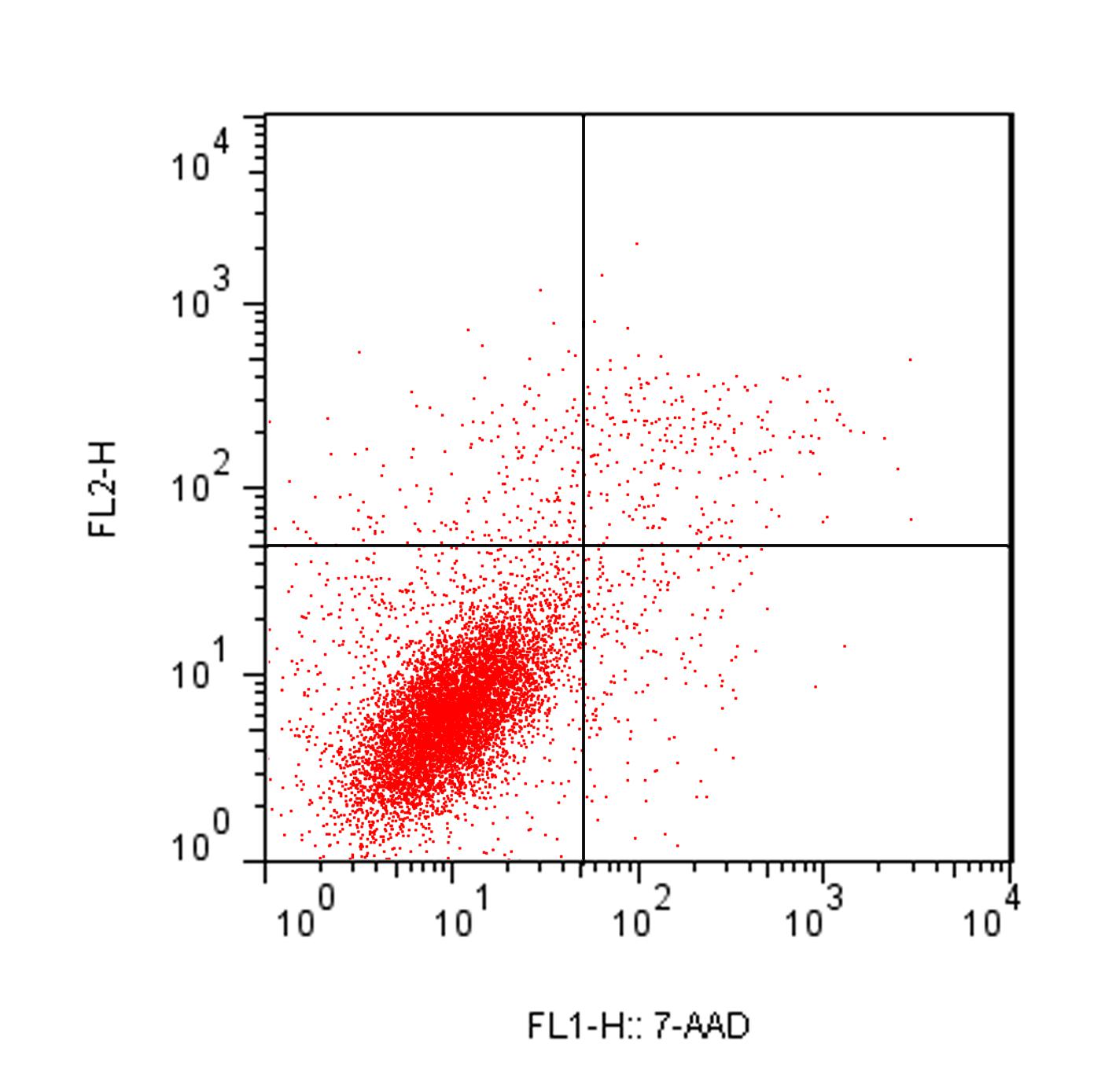

Supplement: Supplementary Figure 1 — MALAT1 was upregulated during osteogenic differentiation. **P<0.01. [file DataSheet_1.zip › MALAT1/流式/Fig.2/control.jpg]

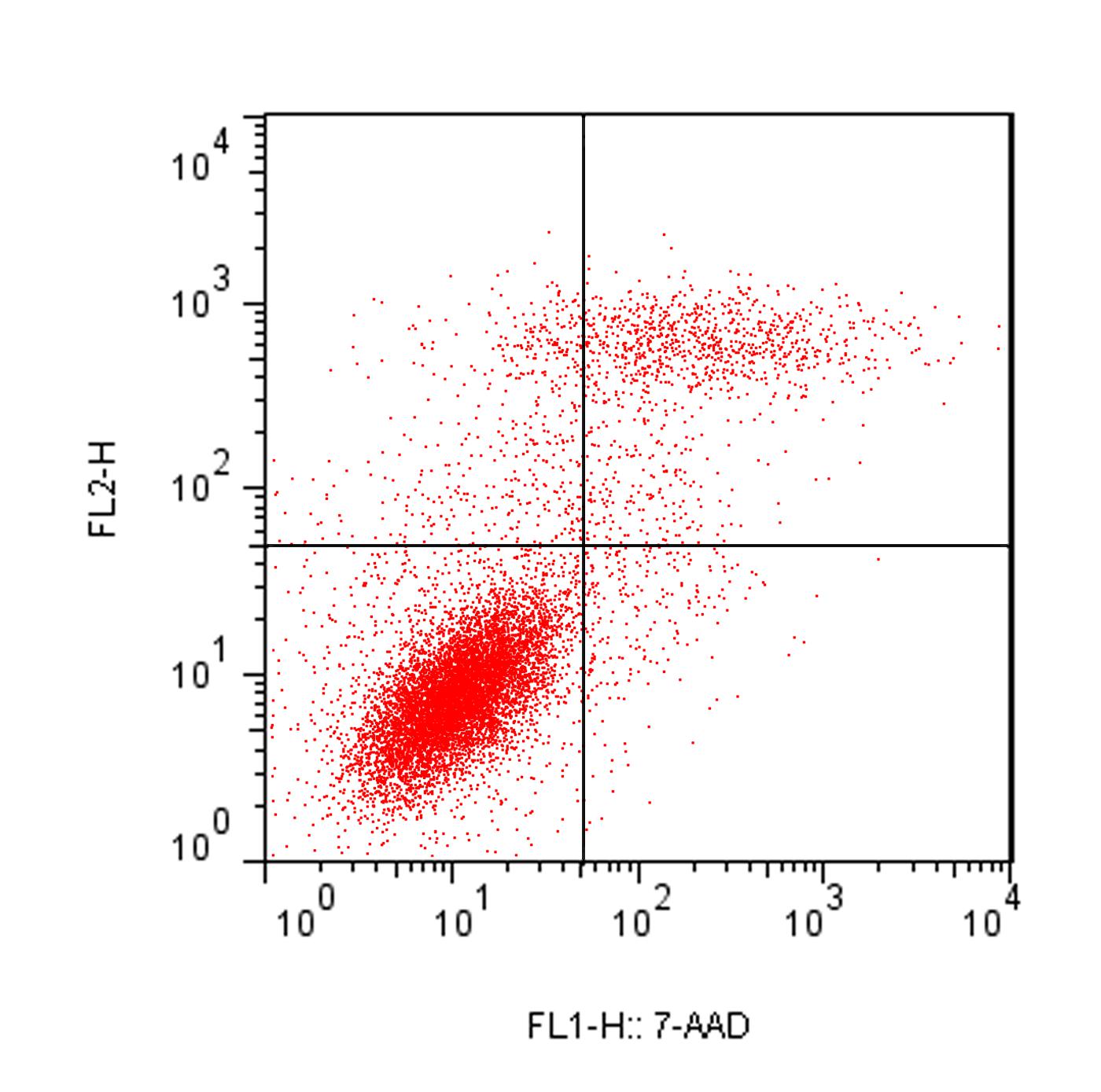

Supplement: Supplementary Figure 1 — MALAT1 was upregulated during osteogenic differentiation. **P<0.01. [file DataSheet_1.zip › MALAT1/流式/Fig.2/MG+MALAT1.jpg]

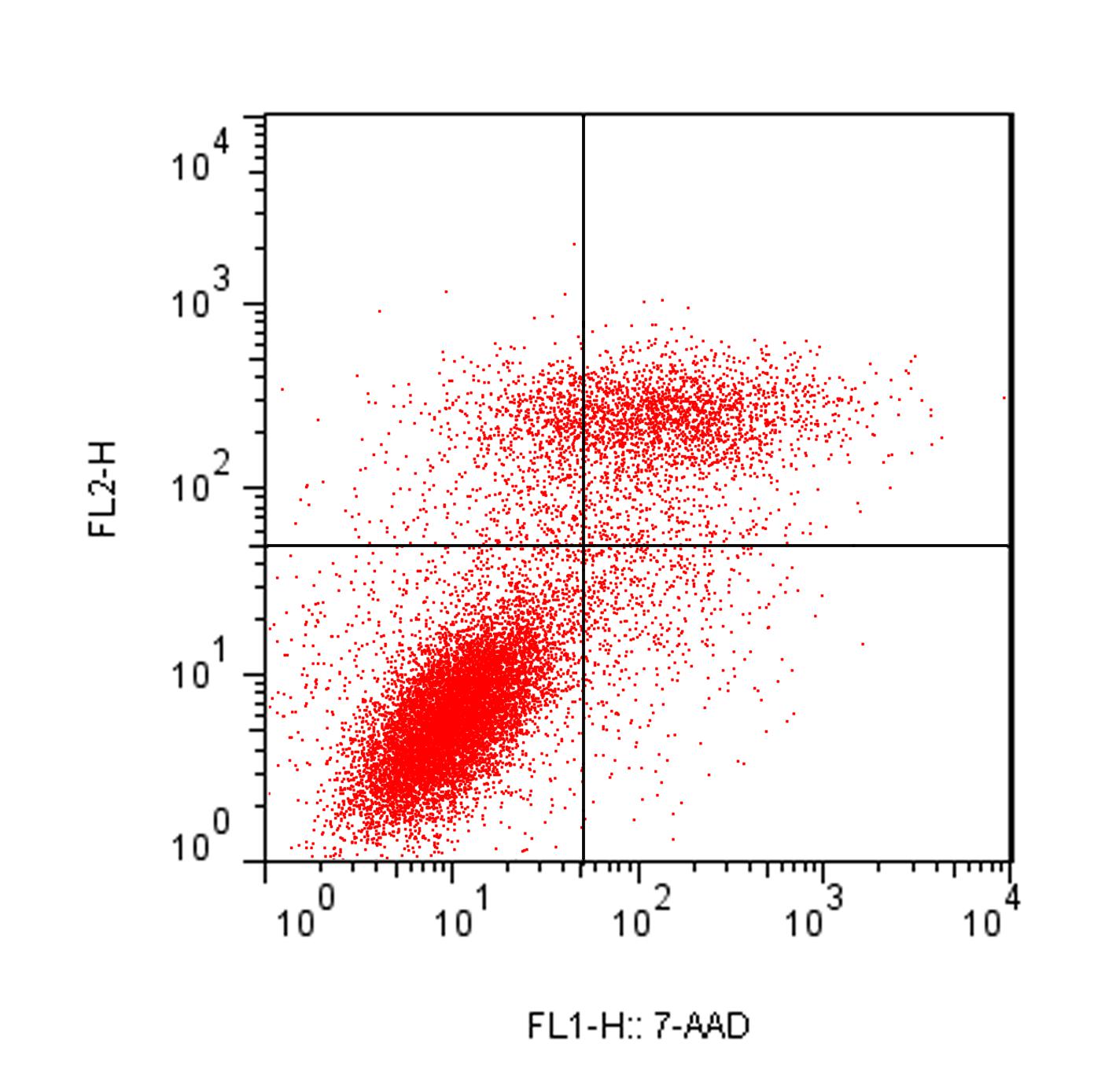

Supplement: Supplementary Figure 1 — MALAT1 was upregulated during osteogenic differentiation. **P<0.01. [file DataSheet_1.zip › MALAT1/流式/Fig.2/MG+vector.jpg]

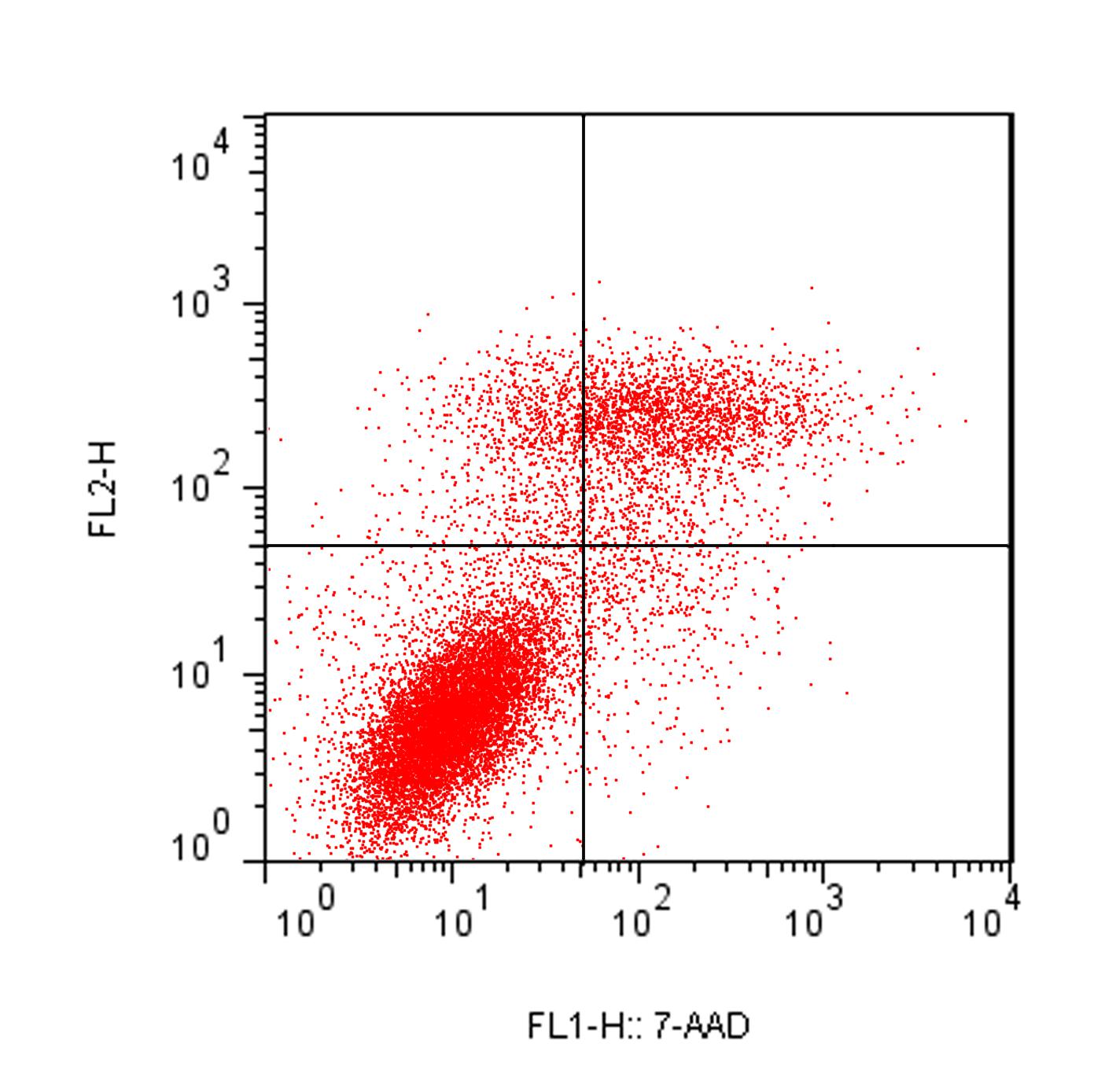

Supplement: Supplementary Figure 1 — MALAT1 was upregulated during osteogenic differentiation. **P<0.01. [file DataSheet_1.zip › MALAT1/流式/Fig.2/MG.jpg]

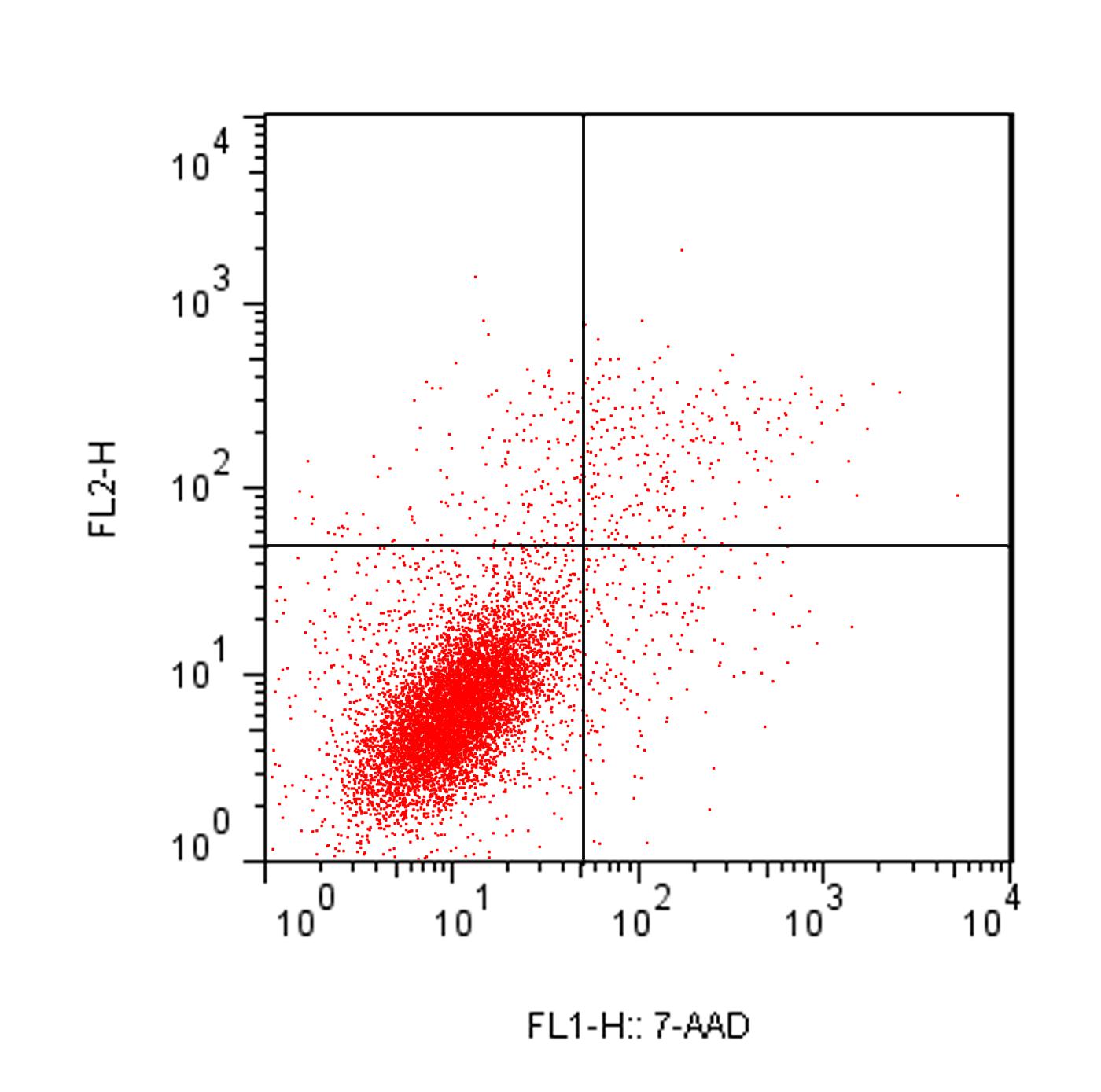

Supplement: Supplementary Figure 1 — MALAT1 was upregulated during osteogenic differentiation. **P<0.01. [file DataSheet_1.zip › MALAT1/流式/Fig.5/control.jpg]

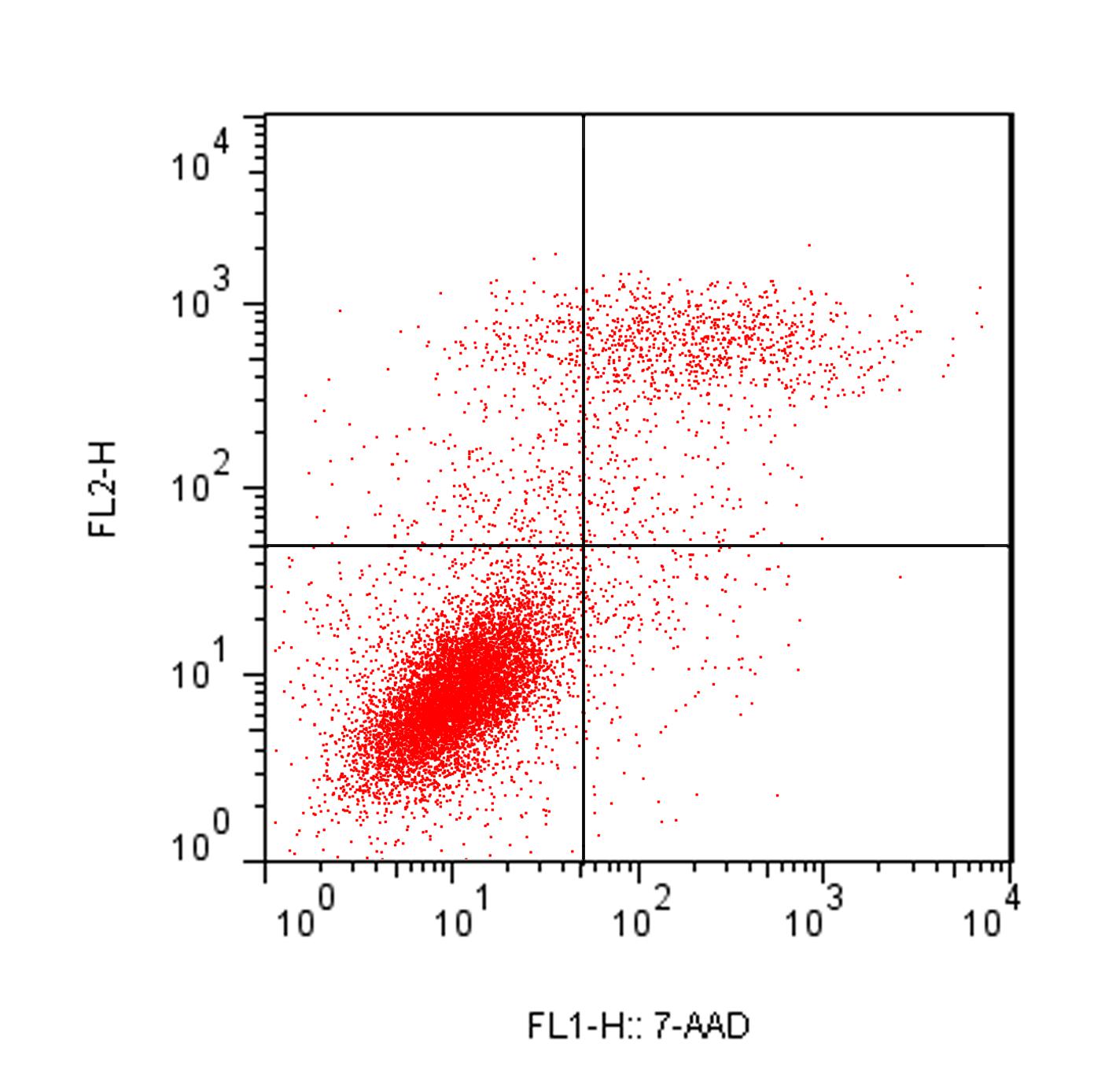

Supplement: Supplementary Figure 1 — MALAT1 was upregulated during osteogenic differentiation. **P<0.01. [file DataSheet_1.zip › MALAT1/流式/Fig.5/MG+MALAT1+mimic NC.jpg]

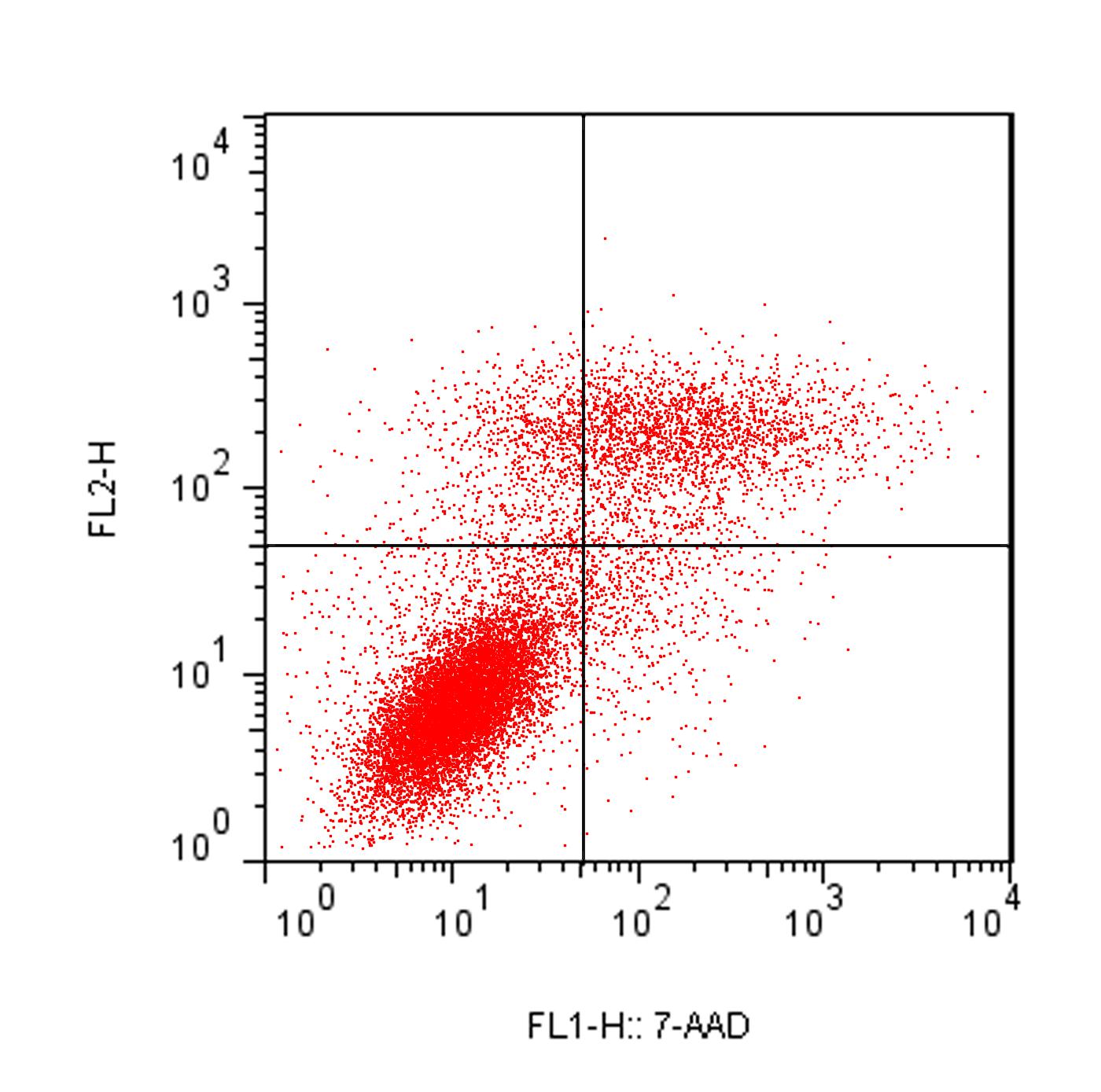

Supplement: Supplementary Figure 1 — MALAT1 was upregulated during osteogenic differentiation. **P<0.01. [file DataSheet_1.zip › MALAT1/流式/Fig.5/MG+MALAT1+mimic.jpg]

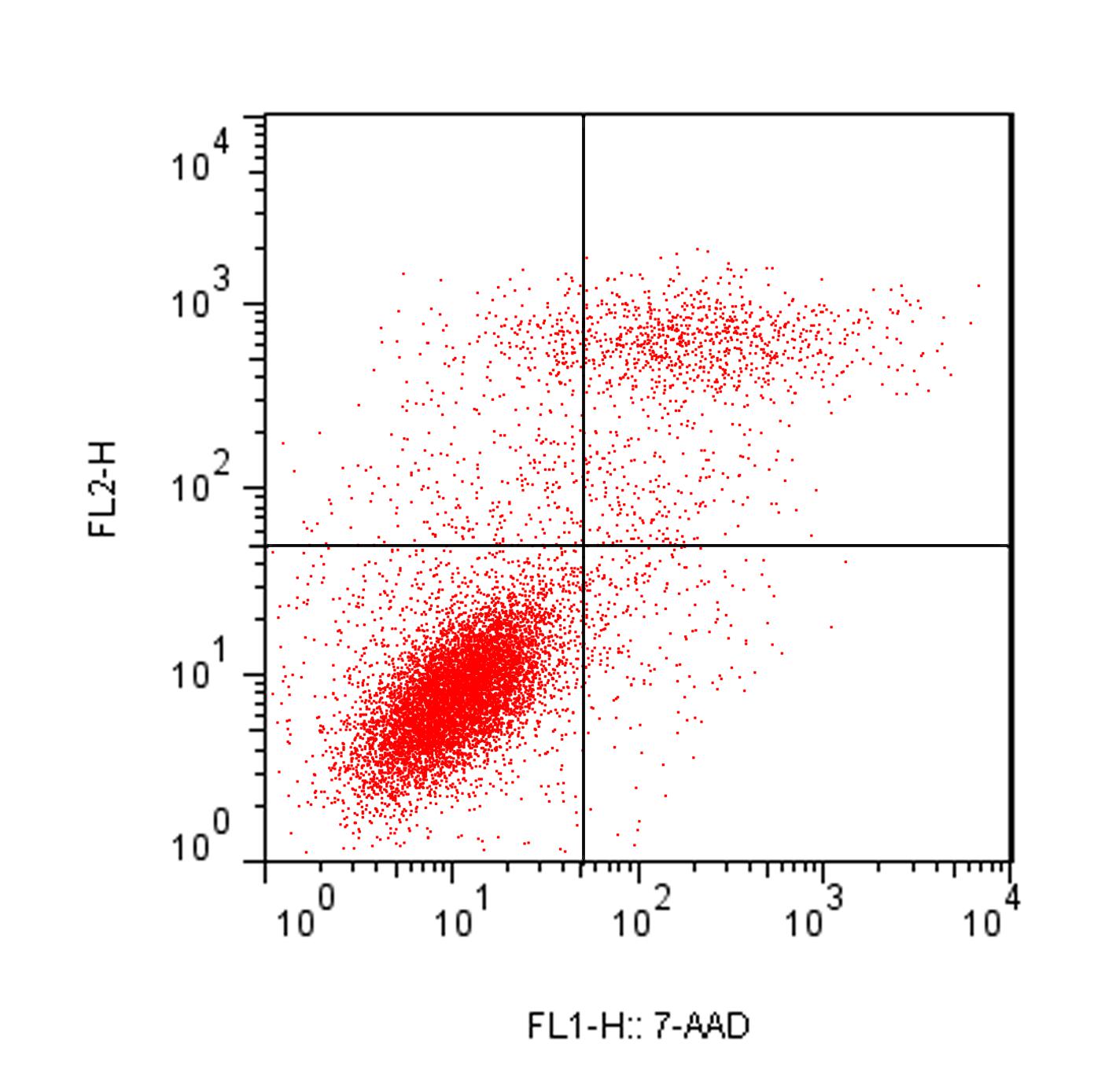

Supplement: Supplementary Figure 1 — MALAT1 was upregulated during osteogenic differentiation. **P<0.01. [file DataSheet_1.zip › MALAT1/流式/Fig.5/MG+MALAT1.jpg]

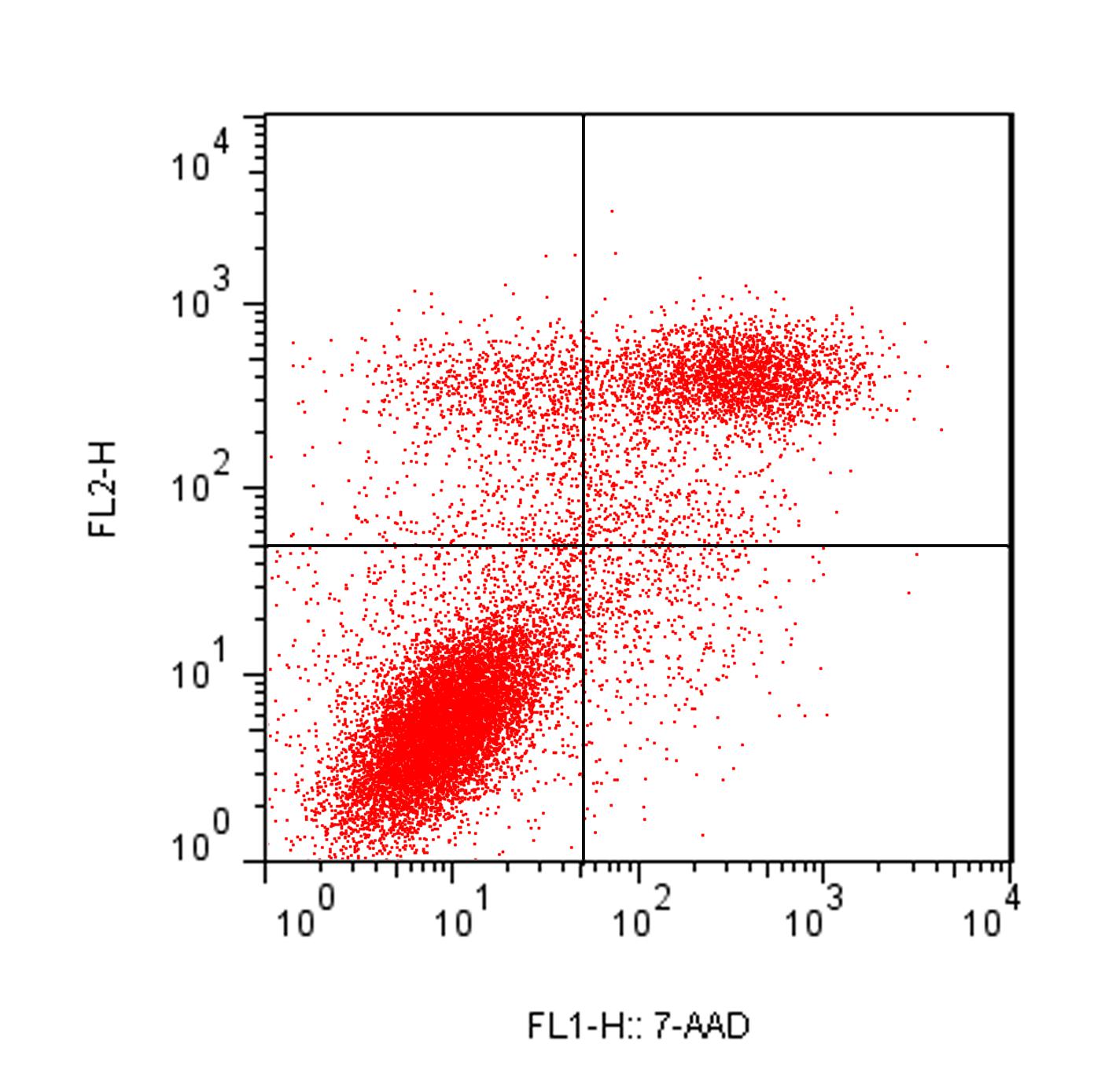

Supplement: Supplementary Figure 1 — MALAT1 was upregulated during osteogenic differentiation. **P<0.01. [file DataSheet_1.zip › MALAT1/流式/Fig.5/MG+vector.jpg]

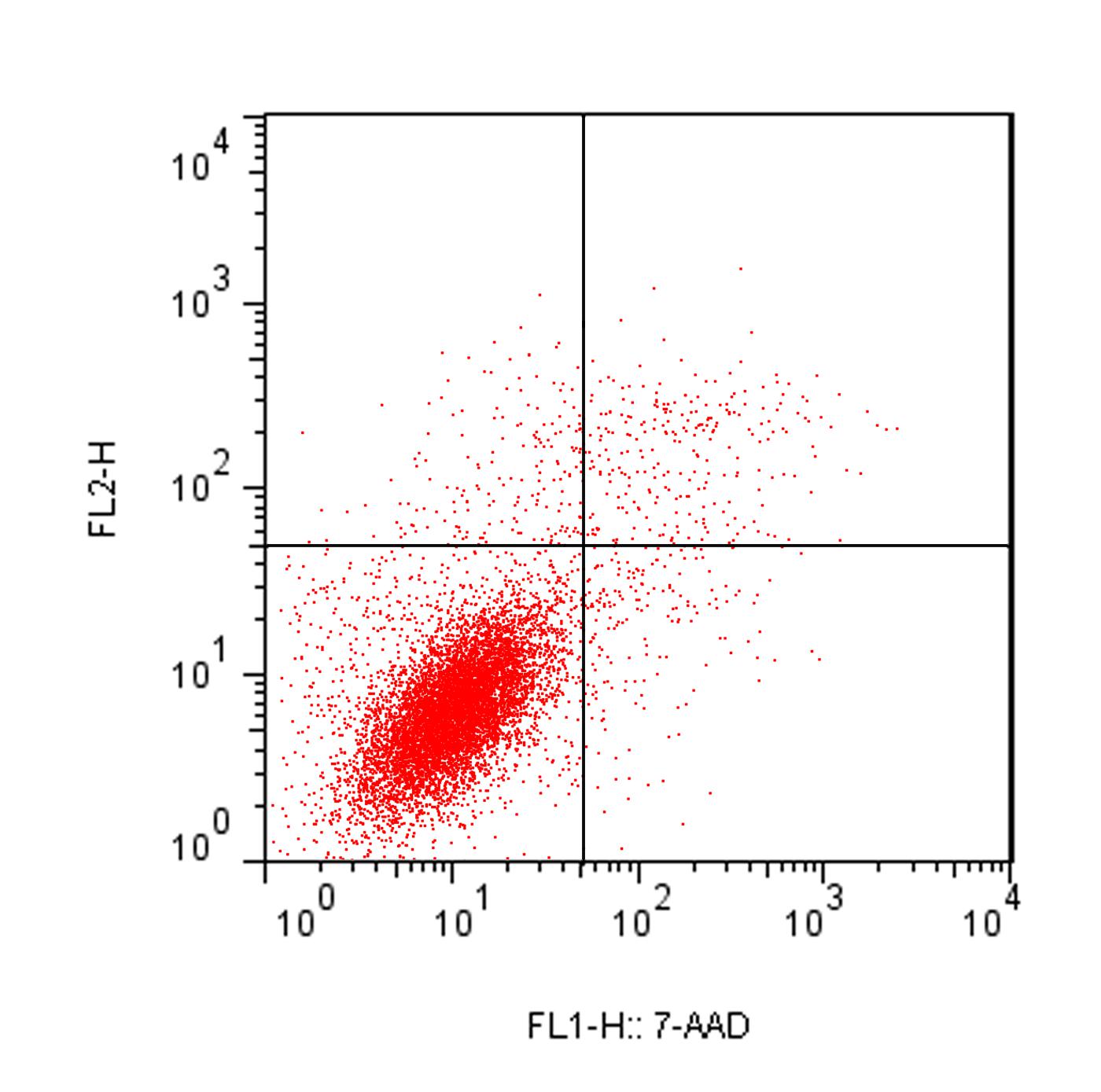

Supplement: Supplementary Figure 1 — MALAT1 was upregulated during osteogenic differentiation. **P<0.01. [file DataSheet_1.zip › MALAT1/流式/Fig.7/control.jpg]

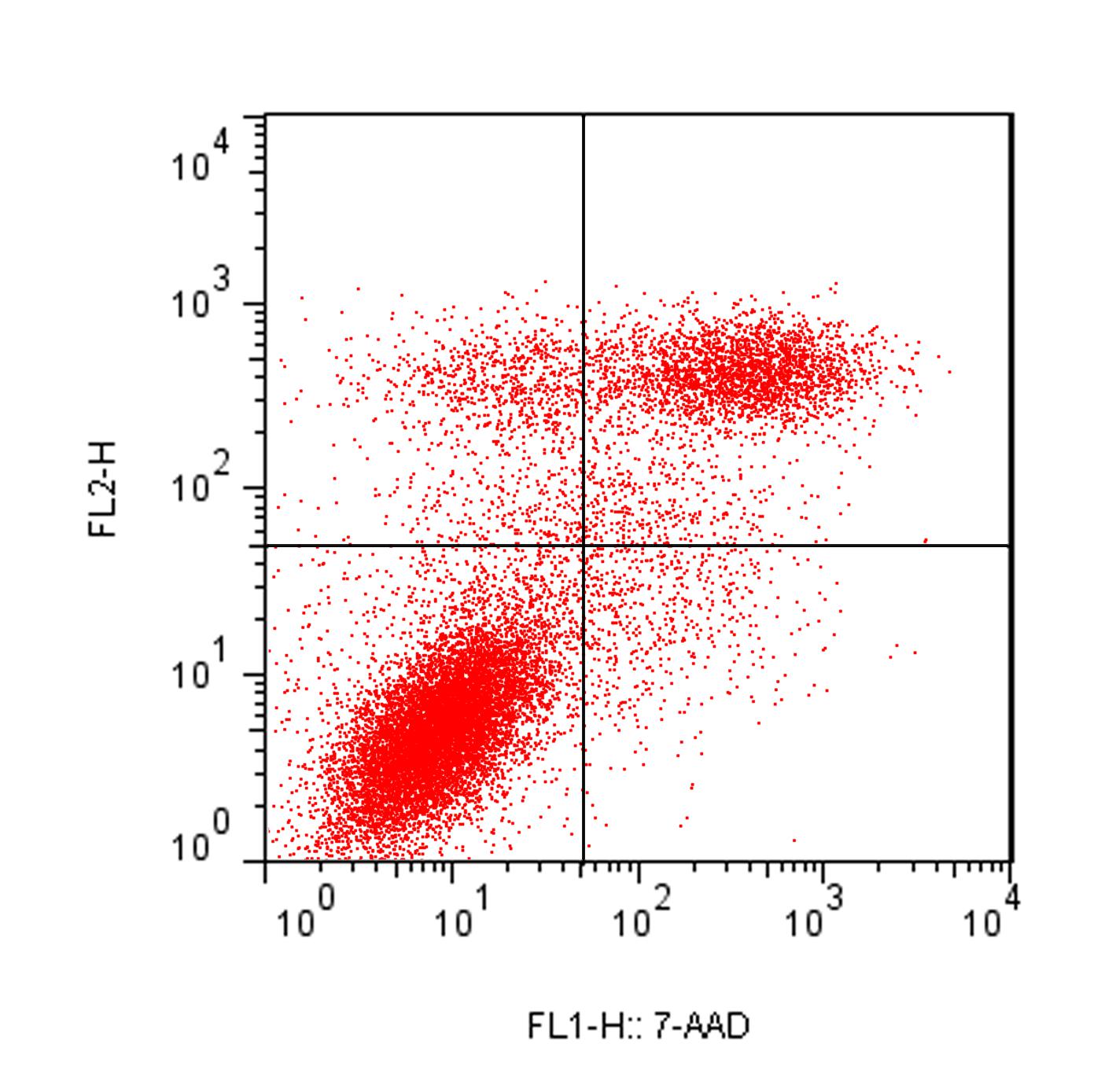

Supplement: Supplementary Figure 1 — MALAT1 was upregulated during osteogenic differentiation. **P<0.01. [file DataSheet_1.zip › MALAT1/流式/Fig.7/MG+inhibitor NC.jpg]

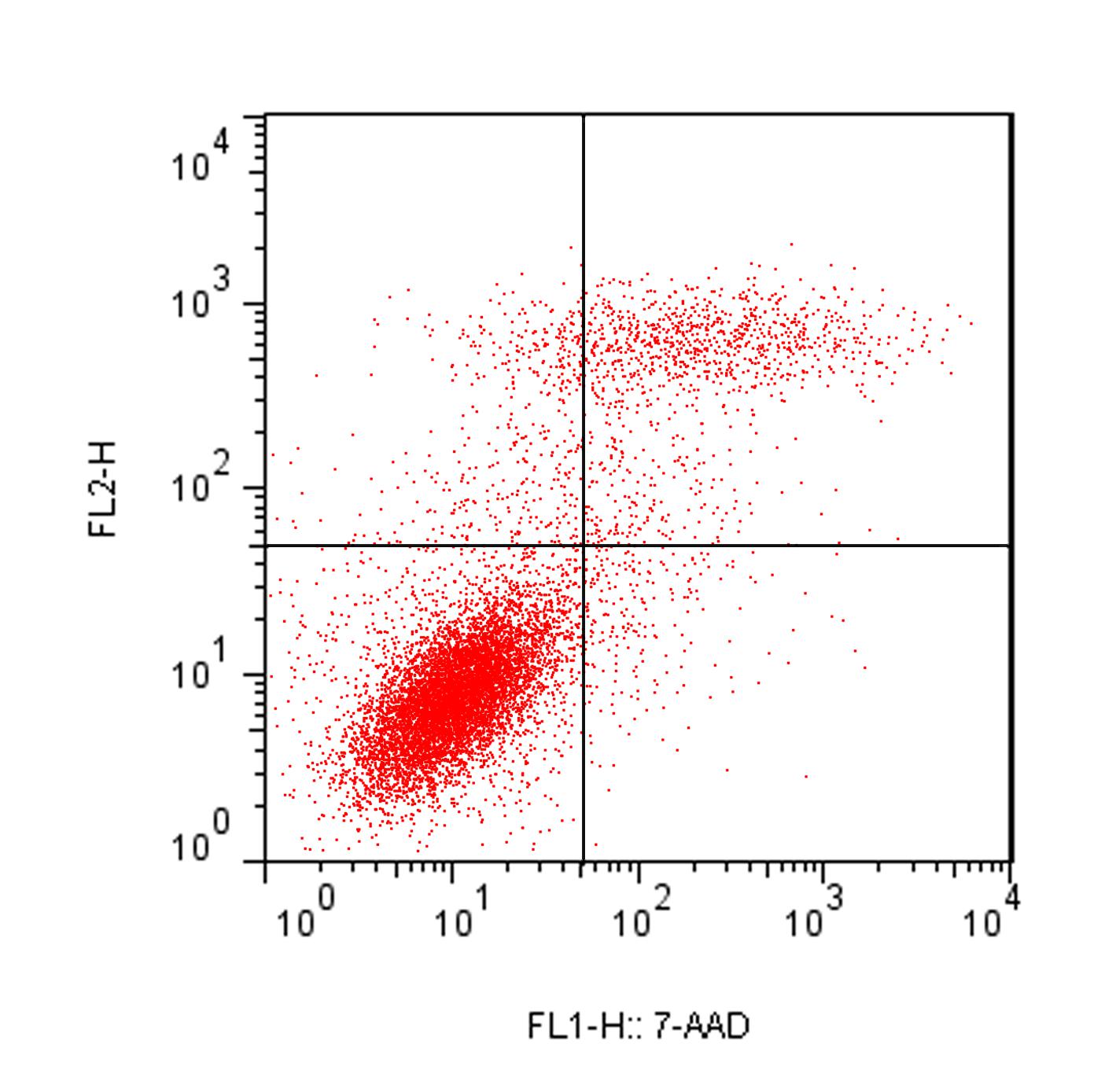

Supplement: Supplementary Figure 1 — MALAT1 was upregulated during osteogenic differentiation. **P<0.01. [file DataSheet_1.zip › MALAT1/流式/Fig.7/MG+inhibitor+si-NC.jpg]

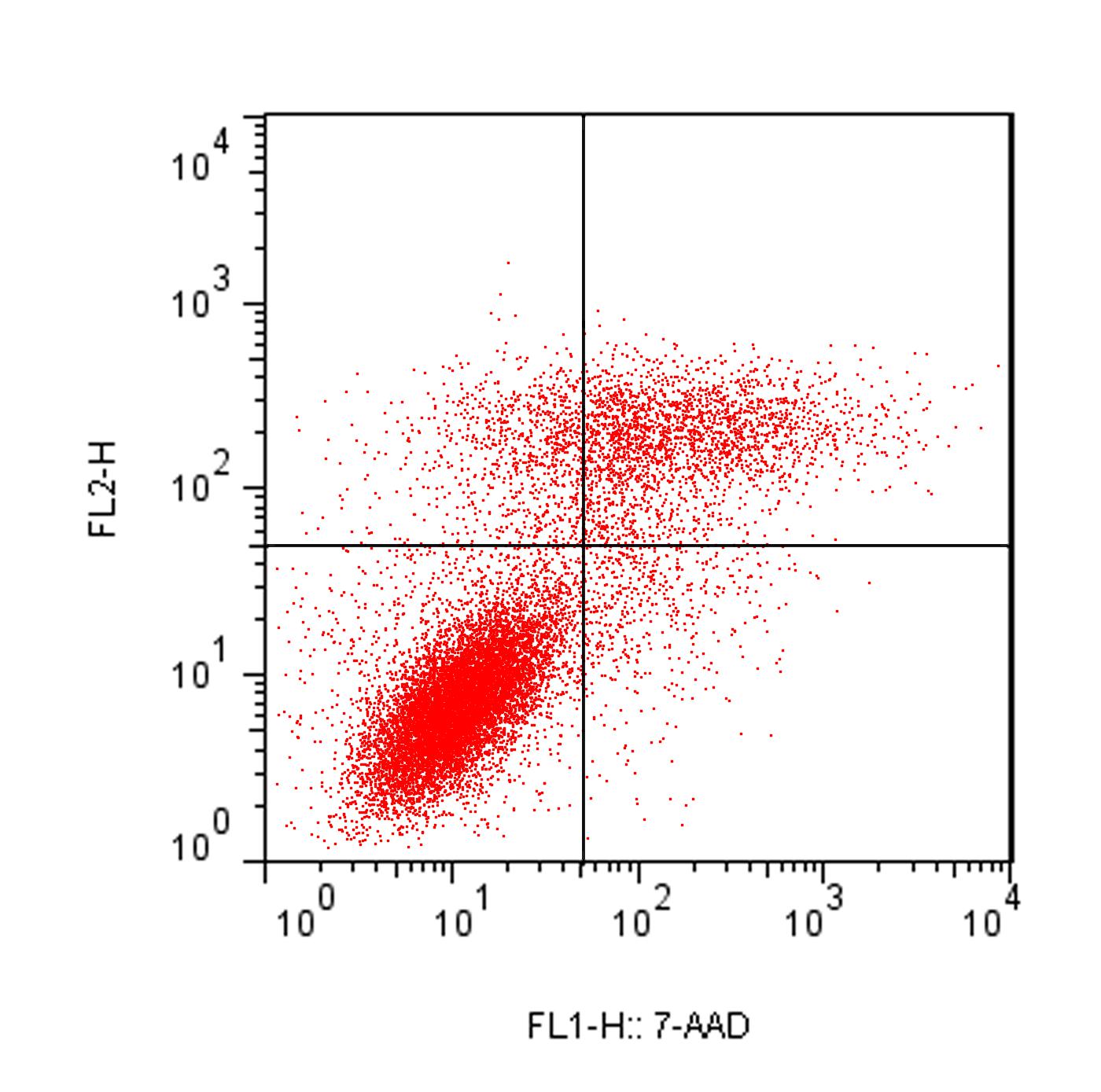

Supplement: Supplementary Figure 1 — MALAT1 was upregulated during osteogenic differentiation. **P<0.01. [file DataSheet_1.zip › MALAT1/流式/Fig.7/MG+inhibitor+si-WNT7B.jpg]

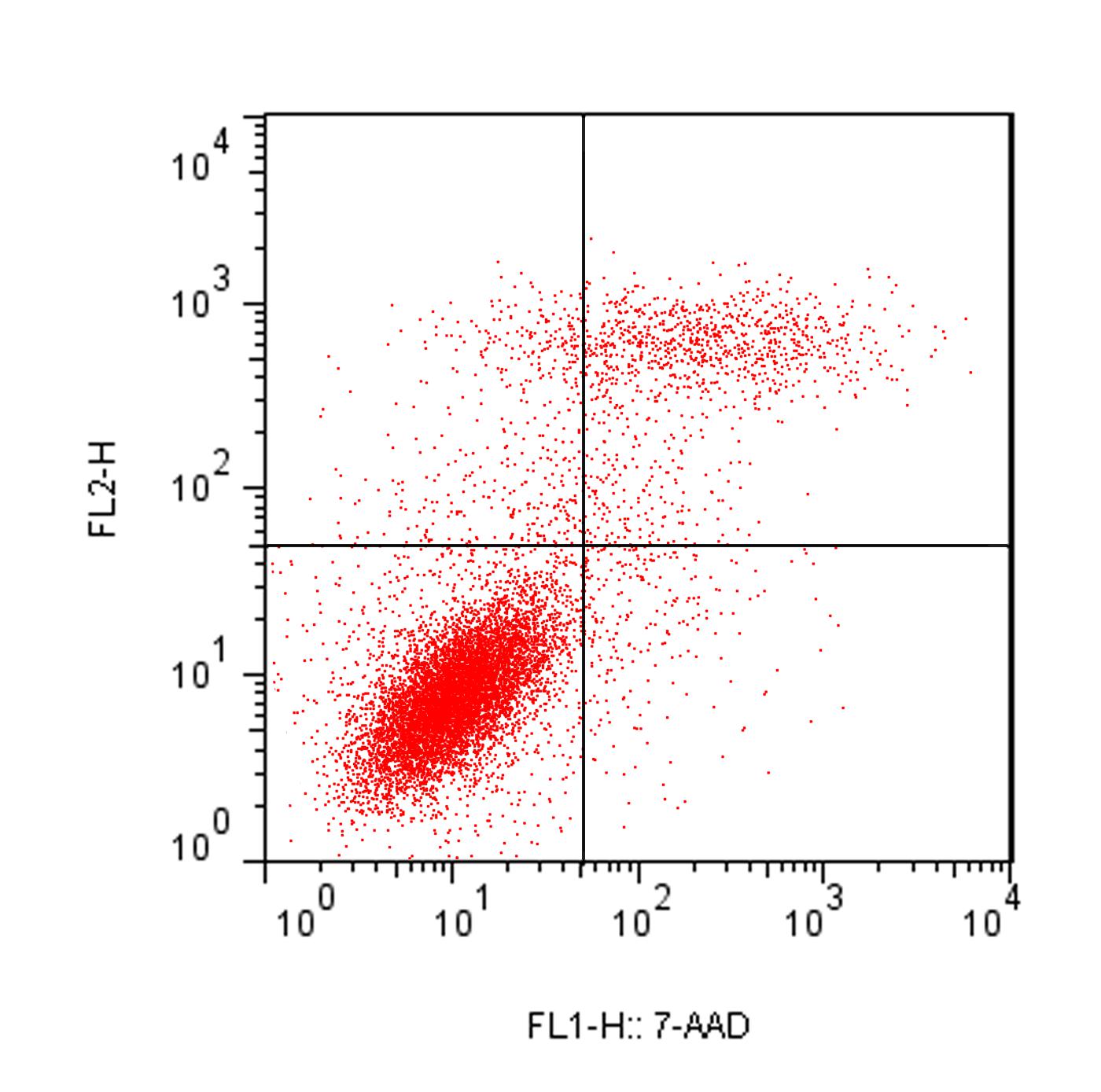

Supplement: Supplementary Figure 1 — MALAT1 was upregulated during osteogenic differentiation. **P<0.01. [file DataSheet_1.zip › MALAT1/流式/Fig.7/MG+inhibitor.jpg]

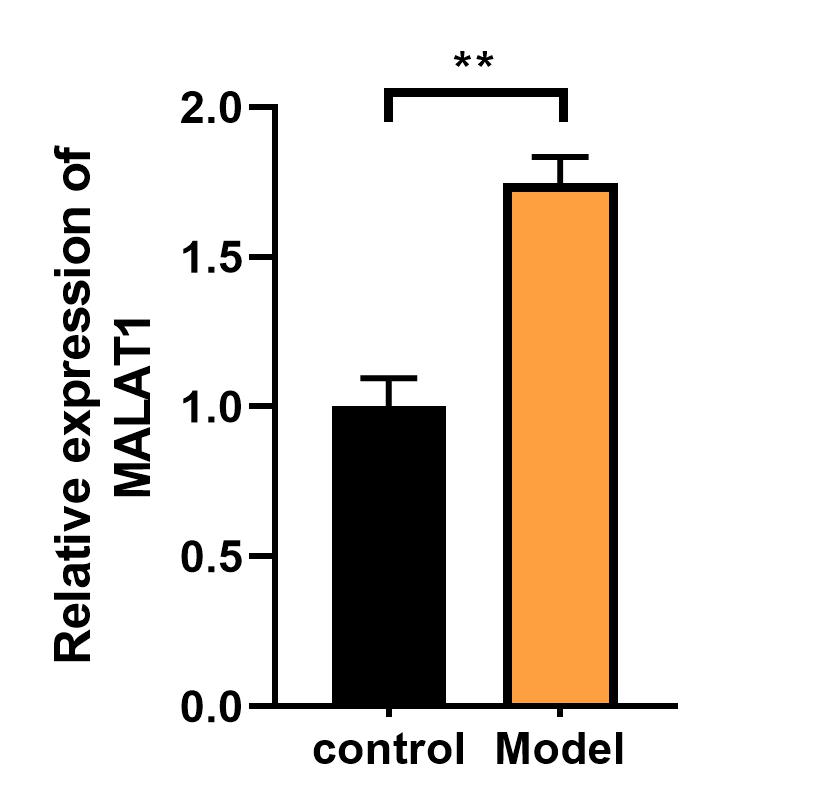

Supplement: Supplementary file 2 [file DataSheet_2.zip › supplementary figures/supplementary figure 1.jpg]

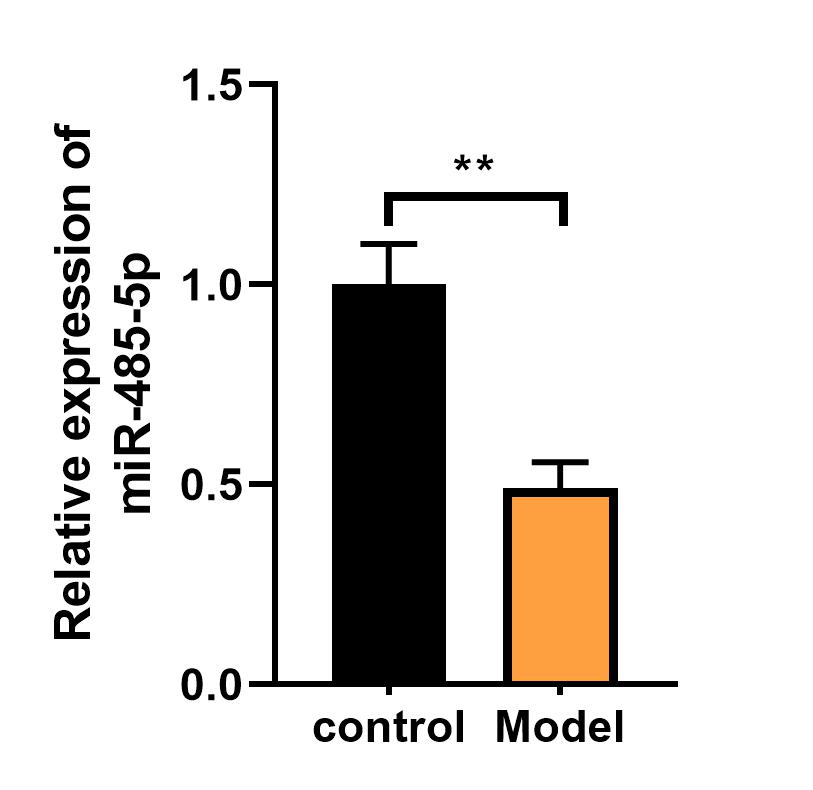

Supplement: Supplementary file 2 [file DataSheet_2.zip › supplementary figures/supplementary figure 2.jpg]

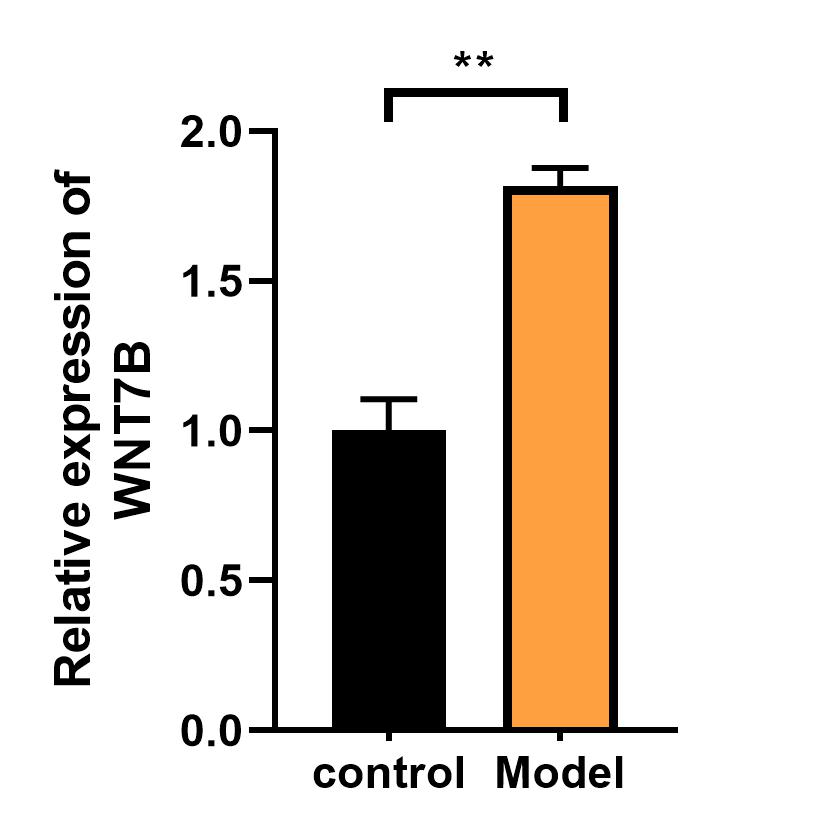

Supplement: Supplementary file 2 [file DataSheet_2.zip › supplementary figures/supplementary figure 3.jpg]

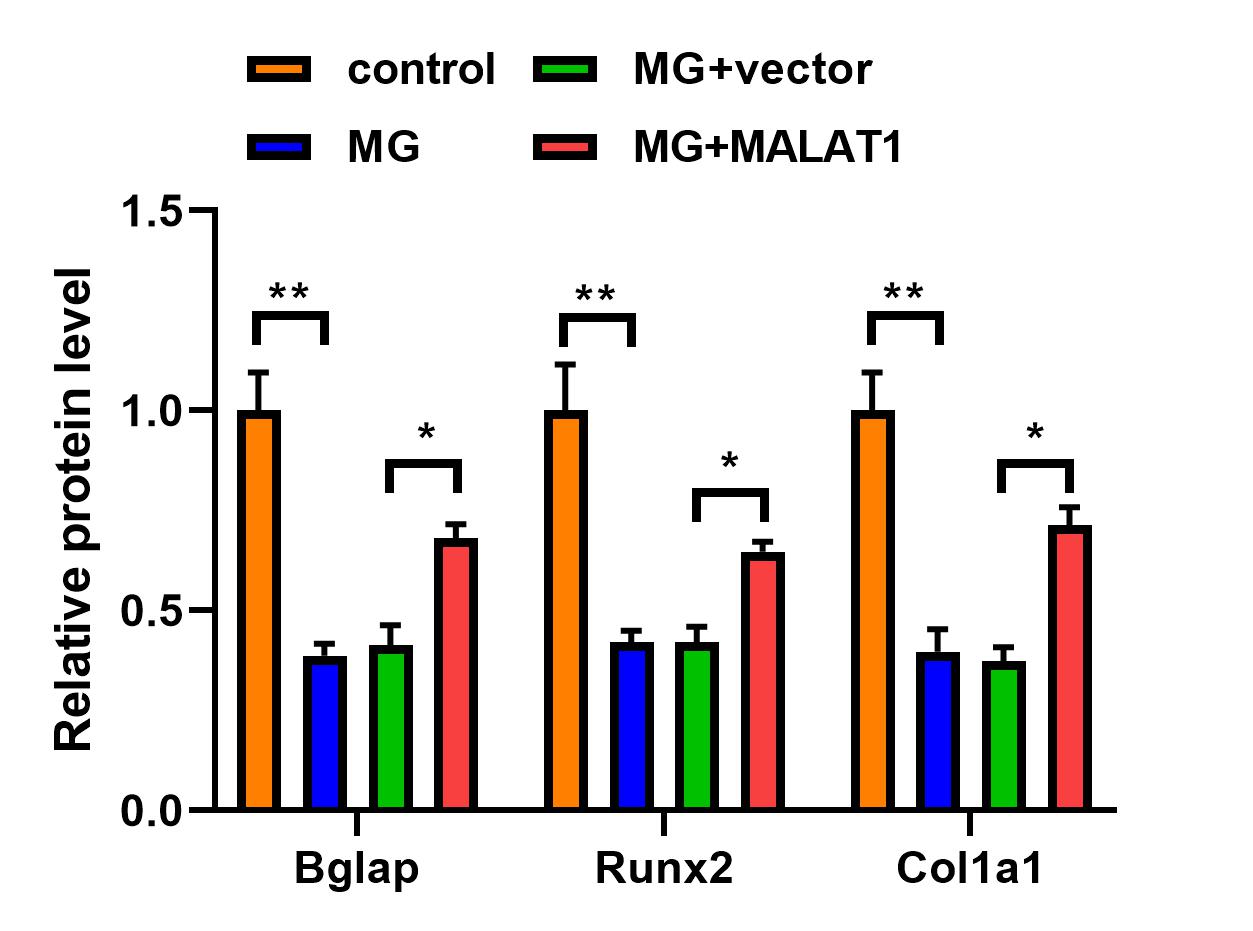

Supplement: Supplementary file 2 [file DataSheet_2.zip › supplementary figures/supplementary figure 4.jpg]

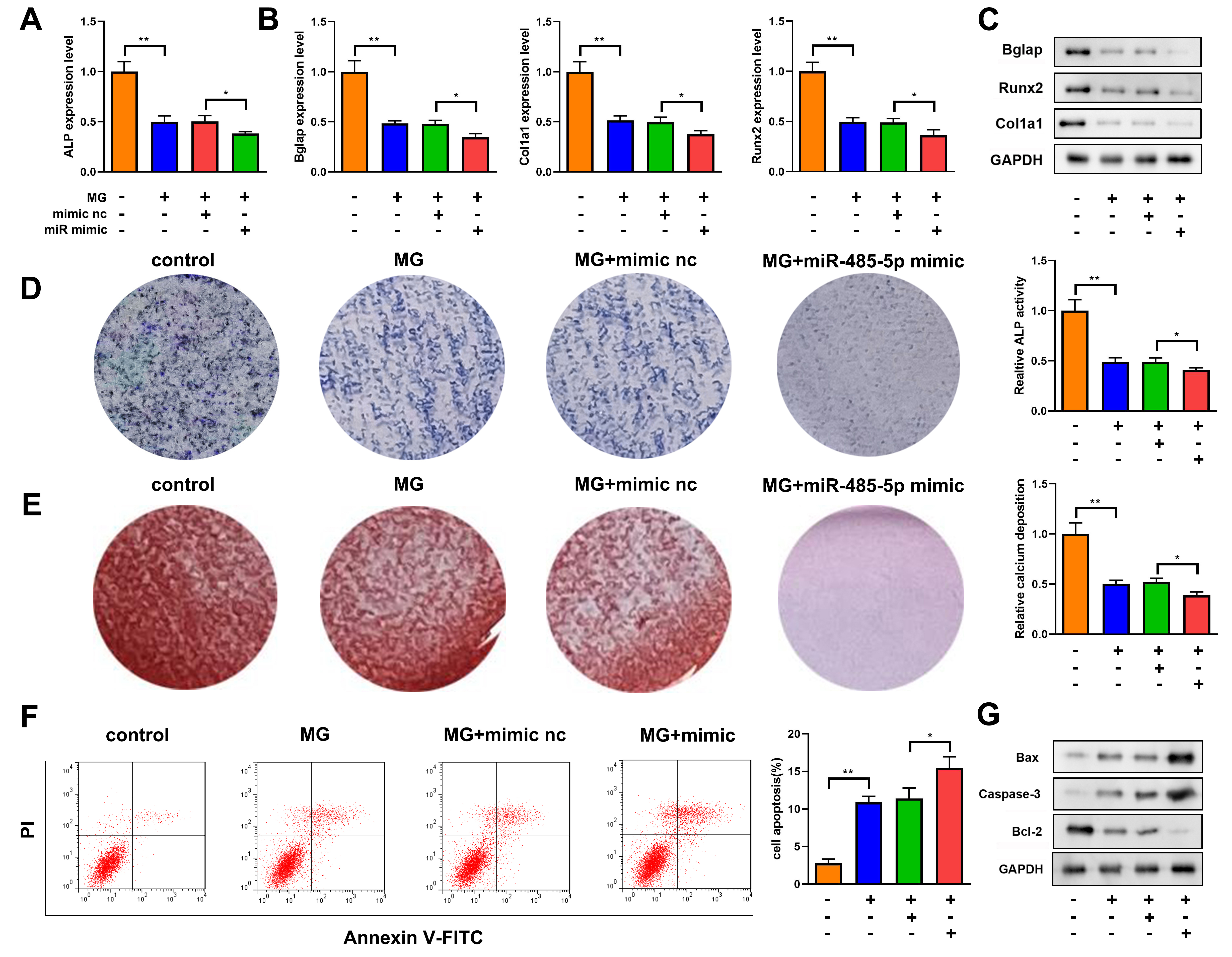

Supplement: Supplementary file 2 [file DataSheet_2.zip › supplementary figures/supplementary figure 5.jpg]

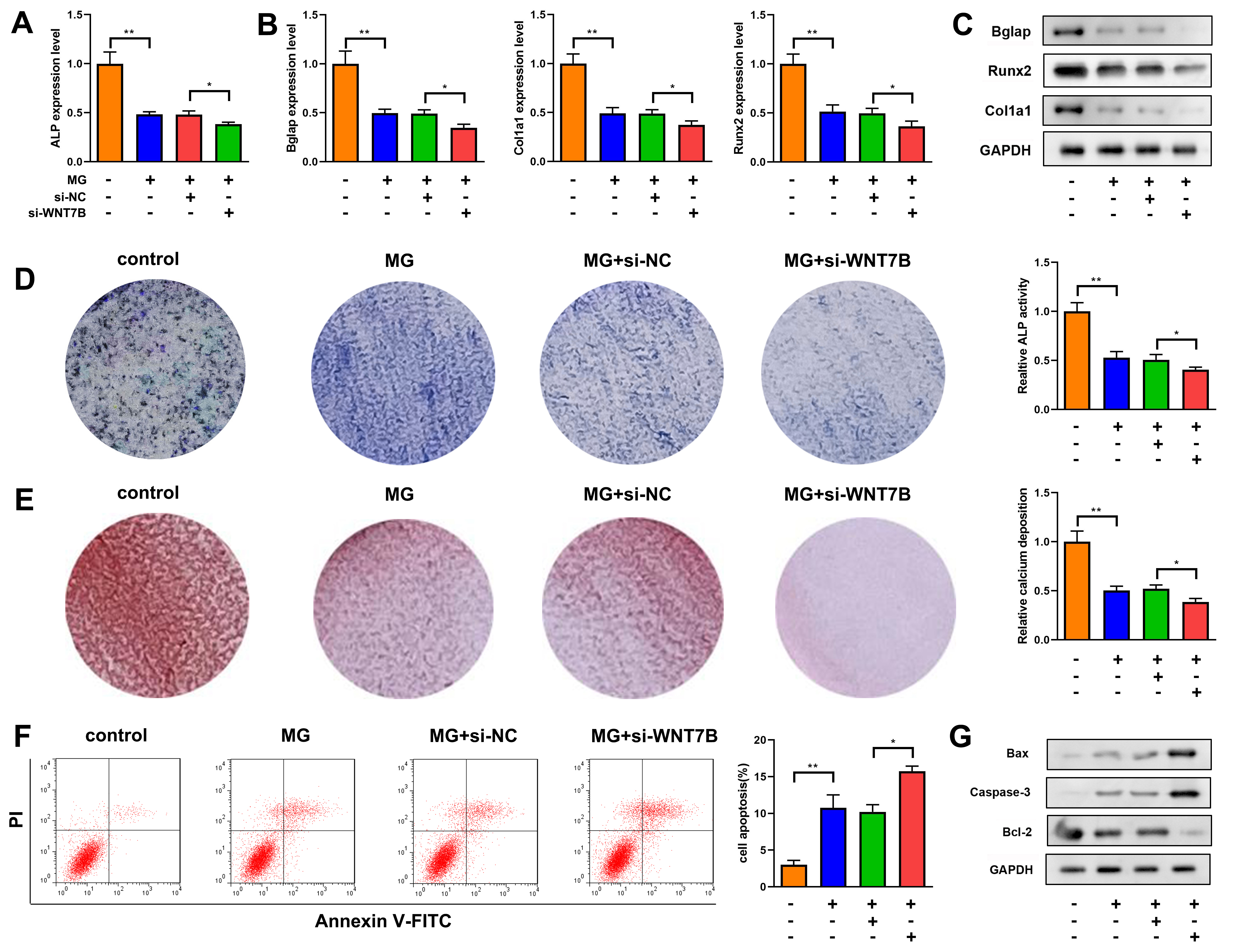

Supplement: Supplementary file 2 [file DataSheet_2.zip › supplementary figures/supplementary figure 6.jpg]

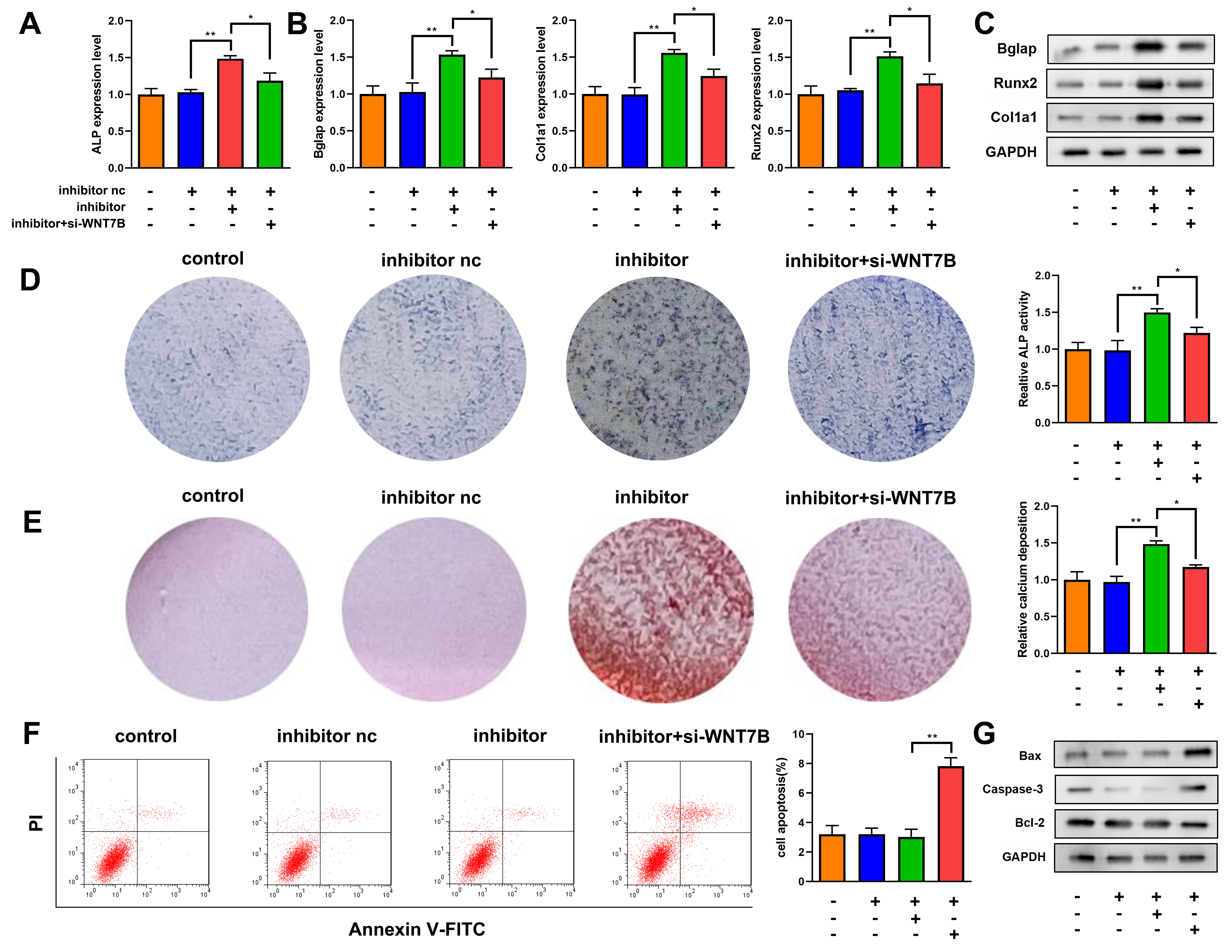

Supplement: Supplementary file 2 [file DataSheet_2.zip › supplementary figures/supplementary figure 7.jpg]
